# Reductive Coupling of Acrylates with Ketones and Ketimines by a Nickel‐Catalyzed Transfer‐Hydrogenative Strategy

**DOI:** 10.1002/anie.201707531

**Published:** 2017-10-04

**Authors:** Craig S. Buxton, David C. Blakemore, John F. Bower

**Affiliations:** ^1^ School of Chemistry University of Bristol Bristol BS8 1TS UK; ^2^ Medicine Design Pfizer Inc. Eastern Point Road Groton CT 06340 USA

**Keywords:** alcohols, lactones, nickel, spiro compounds, transfer hydrogenation

## Abstract

Nickel‐catalyzed coupling of benzyl acrylates with activated ketones and imines provides γ‐butyrolactones and lactams, respectively. The benzyl alcohol byproduct released during the lactonization/lactamization event is relayed to the next cycle where it serves as the reductant for C−C bond formation. This strategy represents a conceptually unique approach to transfer‐hydrogenative C−C bond formation, thus providing examples of reductive heterocyclizations where hydrogen embedded within an alcohol leaving group facilitates turnover.

The identification of catalytic paradigms for the direct and atom‐economical assembly of C−C bonds is a key goal of organic chemistry. Within this context, transfer‐hydrogenative C−C bond formation has emerged as a powerful platform for reaction design. For example, hydrogen borrowing allows the direct α‐alkylation of carbonyl compounds with alcohols by a catalytic dehydrogenation/condensation/reduction sequence (Scheme 1 a).^1^ The related Guerbet reaction effects the dehydrative union of two alcohols, thus providing an efficient method to upgrade bioethanol to butanol (Scheme 1 b).^2^ Krische and co‐workers have pioneered transfer‐hydrogenative alcohol C−H functionalizations as exemplified by processes where alcohol dehydrogenation drives the reductive generation of nucleophilic metal allyls in advance of carbonyl addition (Scheme 1 c).^3^ Each of these reaction classes merges redox events with C−C bond formation, thus avoiding stepwise generation of reactive functionality and enhancing substantially atom economy. As such, new transfer‐hydrogenative C−C bond‐forming strategies are likely to find wide utility in reaction design.

**Scheme 1:**
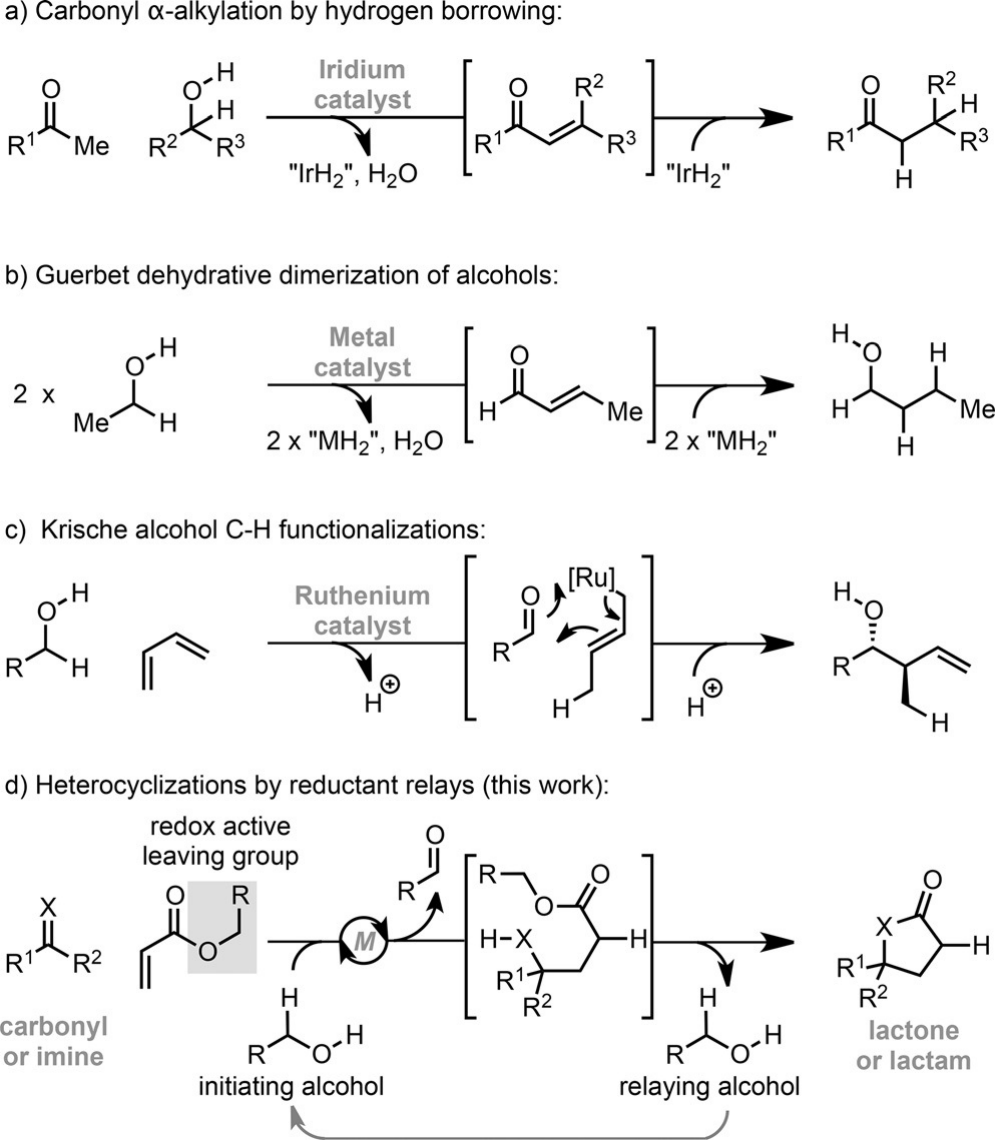
Transfer hydrogenative C−C bond‐forming strategies.

Our studies in this area were initiated by considering synthetic entries to γ‐butyrolactones and lactams,^4^, ^5^, ^6^, ^7^ which are versatile intermediates as well as core motifs in an array of natural products. An appealing, yet unrealized approach to these compounds resides in metal‐catalyzed reductive coupling of either a carbonyl or imine with an acrylate to afford a γ‐amino or γ‐hydroxy ester, which upon cyclization would provide the target (Scheme 1 d). This disconnection requires the identification of a strategy which enables reductive C−C bond formation, but avoids nonproductive reduction of the starting materials. We reasoned that these criteria might be fulfilled by coupling the release of the reductant to the formation of either the lactone or lactam, thereby minimizing nonproductive background reduction events. Such a proposition appears practically challenging, however, a simple solution is availed by harnessing the native reducing power of the alcohol released upon cyclization to drive turnover. In this way, the alcohol byproduct from one cycle is relayed to the next, where it then serves as the reductant for C−C bond formation. Herein, as proof‐of‐concept, we show that lactones and lactams can be generated by nickel‐catalyzed union of activated ketones and ketimines, respectively, with O‐benzyl acrylates. This approach provides unique examples of reductive heterocyclizations where hydrogen embedded within an alcohol leaving group facilitates catalytic turnover,^8^ thus adding a new vista to the wider area of transfer‐hydrogenative C−C bond formation.^1^, ^2^, ^3^

In early studies, we assayed a wide range of late‐transition‐metal systems for the reductive coupling of isatin **1 a** and ethyl acrylate (**2 a,** R=Et; Table 1). At 150 °C in PhMe, and with 10 mol % benzyl alcohol as the initiator (see Scheme 1 d), the combination of 7.5 mol % Ni(cod)_2_ and 15 mol % P(*o*‐OMeC_6_H_4_)_3_ provided the target lactone **3 a** in 19 % yield, with unreacted starting material accounting for the mass balance (Table 1, entry 1). Here, according to our reaction design, ethanol released during the first turnover must then function as the reductant for subsequent cycles. Based on this we considered whether more easily oxidized alcohol‐based leaving groups might provide increased efficiencies.^9^ Ultimately, this led to the reaction conditions outlined in entry 3, which use 300 mol % benzyl acrylate (**2 b**, R=Bn) as the reaction partner, and generate **3 a** in 84 % yield. Some turnover was observed in the absence of the initiating alcohol (entry 4), likely facilitated by hydrolytic release of BnOH from benzyl acrylate under the reaction conditions. This generates acrylic acid as a byproduct, a component which control experiments found to be inhibitory to the reductive lactonization process.^10^ Lower loadings of either the benzyl alcohol initiator or the nickel pre‐catalyst resulted in diminished efficiencies (entry 5), and use of stoichiometric BnOH also resulted in a lower yield (entry 6). This latter result highlights the benefits of coupling reductant release to turnover. **3 a** was generated in 58 % yield when the reaction was run with only 100 mol % **2 b** (entry 7). A nickel(0) pre‐catalyst is essential for efficient reactivity; nickel(II) systems (e.g. entry 8) or commonly employed transfer‐hydrogenation catalysts, such as [IrCp*Cl_2_]_2_ (entry 9), were completely ineffective.^11^

**Table 1:** Preliminary results and optimization studies. 
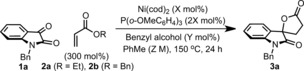

Entry	R	Pre‐catalyst	X	Y	Z	Yield [%]^[a]^
1	Et	Ni(cod)_2_	7.5	10	0.05	19
2	Bn	Ni(cod)_2_	7.5	10	0.05	76
3	Bn	Ni(cod)_2_	7.5	10	0.2	84
4	Bn	Ni(cod)_2_	7.5	0	0.05	17
5	Bn	Ni(cod)_2_	5	5	0.05	38
6	Bn	Ni(cod)_2_	7.5	100	0.2	52
7^[b]^	Bn	Ni(cod)_2_	7.5	10	0.2	58
8	Bn	NiCl_2_	7.5	10	0.2	<5
9	Bn	[IrCp*Cl_2_]_2_	3.75	10	0.2	<5

[a] Yield determined by ^1^H NMR analysis using 1,4‐dinitrobenzene as an internal standard. [b] Using 100 mol % benzyl acrylate (**2 b**). cod=1,5‐cyclooctadiene, Cp*=C_5_Me_5_.

The scope of the process with respect to the isatin component is outlined in Table 2. A variety of electronically distinct systems (**1 a**–**j**) participated to provide the target spirocyclic systems **3 a**–**j** in moderate to excellent yield. The protocol shows useful functional‐group tolerance, with both esters (**3 h**) and methoxy (**3 d**) substituents surviving, despite the established lability of these functionalities under nickel(0)‐catalyzed conditions.^12^ Processes involving disubstituted acrylates required the addition of Mg(OTf)_2_ as a Lewis‐acidic co‐catalyst.^13^ By using this modification, reductive coupling of **1 a** with α‐methyl (**2 c**) and α‐phenyl (**2 d**) benzyl acrylate provided the targets **3 k** and **3 l**, respectively, in high yield and as single diastereomers (>20:1 d.r.). The relative stereochemistries of **3 k** and **3 l** were assigned by X‐ray diffraction.^14^ Interestingly, these products possess opposite relative configurations. β‐Substituted acrylates also participate, such that targets **3 m** and **3 n** were formed in 77 and 73 % yield, respectively. In the latter case, the Lewis acid co‐catalyst was not required, likely due to the high electrophilicity of the acrylate partner, dibenzylfumarate **2 f**.

**Table 2:** Reductive coupling of benzyl acrylate with isatins.

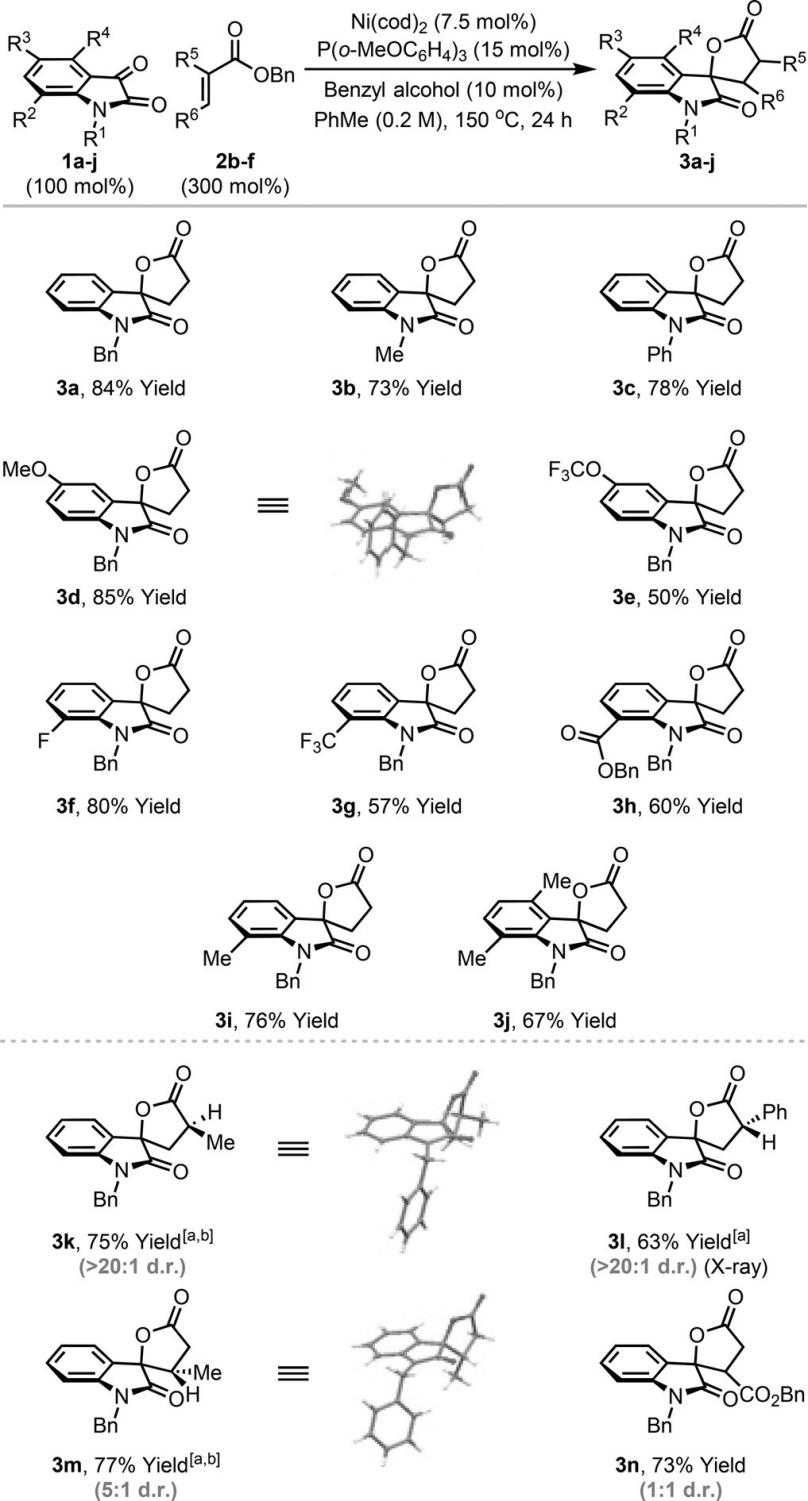

Yields are those of isolated products. [a] Mg(OTf)_2_ (10 mol %) was used as an additive. [b] 600 mol % of acrylate was used.

Our observations are that isatins are privileged substrates for this reductant relay process. Nevertheless, we have established that, in certain cases, other classes of 1,2‐dicarbonyl also participate, thus suggesting potentially wider applications of the strategy. For example, benzil systems **4 a**–**c** generated the corresponding monocyclic lactones **5 a**–**c** in modest to very good yield (Scheme 2). Cyclic system **6** was also a competent reaction partner, generating lactone **7** in 52 % yield when Mg(OTf)_2_ was used as co‐catalyst. As far as we are aware, the examples in Table 2 and Scheme 2 are the first catalytic reductive lactonizations which harness carbonyls and unfunctionalized acrylate esters. Existing noncatalytic protocols use exogenous stoichiometric reductants,^5^ whereas catalytic approaches require alcohols as the starting material, and, in turn, mandate prior reduction of the carbonyl partner.^4^

**Scheme 2:**
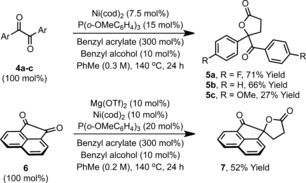
Reductive coupling of benzyl acrylate with activated ketones.

To probe the mechanism of the process a series of experiments was undertaken. When *deuterio*‐**2 b**, which incorporates deuterium at the benzylic positions, was exposed to the optimized reaction conditions, 40 % deuterium transfer to C2 of *deuterio*‐**3 a** was observed (Scheme 3 a). Significant deuterium incorporation was also found at C2′, thus indicating that the nickel(0) system can also activate the N‐benzylic position.^15^ For **1 a** to **3 a** (84 % Yield), GCMS analysis of the crude reaction mixture revealed the concomitant formation of benzaldehyde in 78 % yield. These observations show that the benzyloxy unit of the acrylate partner (**2 b**) acts as the reductant for C−C bond formation. Under optimized reaction conditions we have confirmed that benzyl acrylates are most effective (Scheme 3 b). Other systems with either primary or secondary alcohol based leaving groups, such as methyl, ethyl, and cyclohexyl acrylate, also enabled turnover, but provided **3 a** in significantly diminished yields. Conversely, phenyl and *tert*‐butyl acrylate, which release “non‐oxidizable” phenol or *t*BuOH, did not allow turnover, with the yield of **3 a** limited to the loading of the benzyl alcohol initiator (10 mol %). Overall, these observations are consistent with the reductive formation of γ‐hydroxy ester **9**, in advance of lactonization to give **3 a** (Scheme 3 c). Intermediate **9** might arise by either a carbonyl reduction/conjugate addition pathway (Path a)^16^ or an oxidative coupling/reduction sequence (Path b).^17^, ^18^ Two key observations provide circumstantial support for Path a: 1) an adjacent acidifying group is required on the carbonyl partner^19^ and 2) products of oxidative coupling with the benzaldehyde byproduct are not formed.^20^ The beneficial effects of Mg(OTf)_2_ in certain cases would be consistent with Lewis acid activation of the acrylate for conjugate addition. Exposure of **8** (the reduced form of **1 a**) to the optimized reaction conditions, with either **2 b** or **2 c**, generated **3 a** in high yield (Scheme 3 d). Lactone formation from **8** in the absence of the nickel catalyst was feasible, but resulted in low conversion to **3 a** (15 % yield). Thus, if Path a is operative, the nickel catalyst must play an intimate role in enhancing the C−C bond‐forming event. One possibility is that oxidative addition of nickel(0) into the C3−H bond of **8** generates a nickel enolate, a process which has been suggested in other contexts.^21^ Exposure of **8** to benzaldehyde (100 mol %) under standard catalytic conditions (in the absence of acrylate) resulted in a 35 % yield of **1 a**, thus showing that reduction of **1 a** is reversible. Because of this, initial oxidation of **8** to **1 a** in advance of spirolactonization by Path b cannot be ruled out. As already discussed, either nickel(II) systems or commonly employed ruthenium‐ and iridium‐based transfer‐hydrogenation catalysts do not promote the reaction, thus supporting a role for the nickel(0) system beyond simply effecting transfer hydrogenation of **1 a**.

**Scheme 3:**
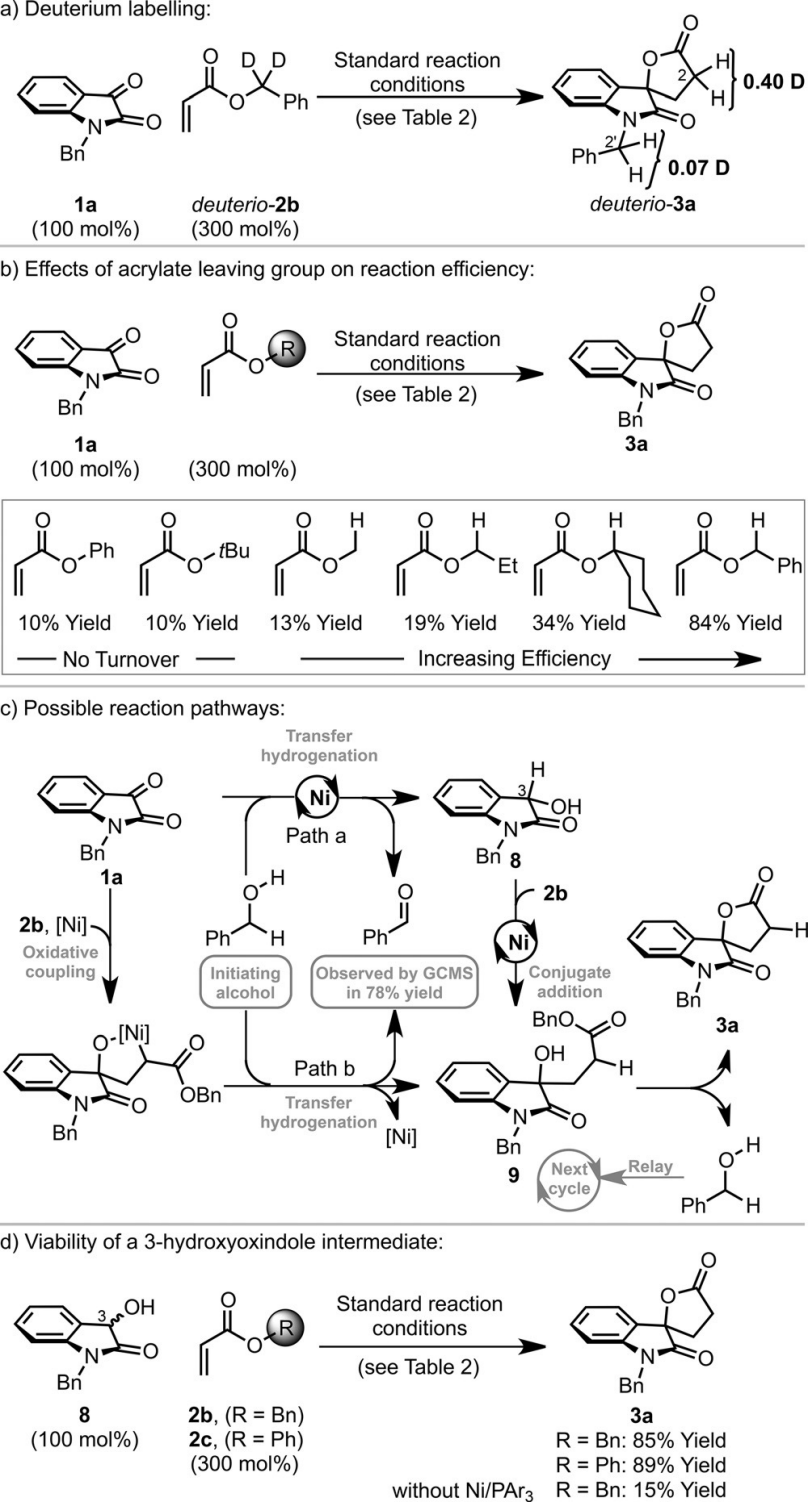
Preliminary mechanistic studies.

According to the mechanistic blueprint outlined in Scheme 1 d, other classes of process might be achievable using a reductant relay approach. Although further expansion of the strategy will require the identification of new catalysts and/or fragment coupling steps, we were keen to uncover additional processes which might be achieved using the nickel(0) system presented here. Specifically, we envisaged that α‐oxo imines might couple with acrylates to provide lactams. This proposition was appealing because only sparse reports document the use of stoichiometric metallic reductants to achieve this seemingly simple process, and no catalytic approaches are available.^7^ Pleasingly, when the *N‐p*‐methoxyphenyl imine **10 a** was exposed to the reaction conditions optimized for lactonization, spirocyclic lactam **11 a** was generated in 68 % yield (Table 3). Further evaluation revealed that this lactamization process has similar scope to the lactonization methodology. Indeed, electronically diverse isatin‐based imines (**10 b**–**e**) all engaged in smooth reductive coupling to provide lactam targets **11 b**–**e** in good to excellent yield. Extension of the protocol to the imine derived from benzil **4 b** provided monocyclic system **12** in 68 % yield; the alternate lactone product was not observed. We also investigated a one‐pot imine formation/lactamization sequence (Scheme 4). Exposure of **1 a** to *p*‐methoxyaniline under acidic conditions generated imine **10 a**. Removal of the volatile components was followed by direct addition of the reagents required for reductive lactamization, allowing a telescoped synthesis of **11 a** in 50 % yield over the one‐pot, three‐component process.

**Scheme 4:**
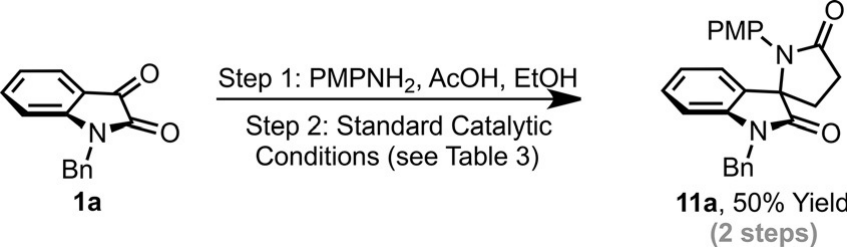
One‐pot imine formation/lactamization sequence.

**Table 3:** Reductive coupling of benzyl acrylate with ketimines.

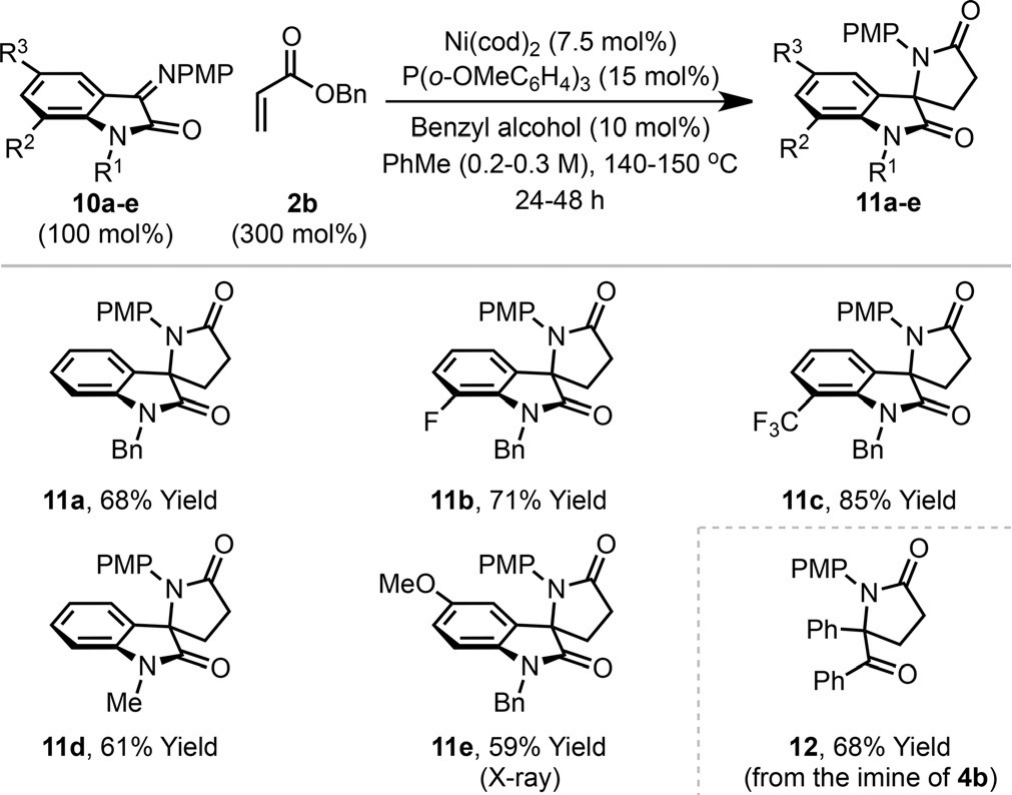

Yields are those of isolated products.

In summary, we demonstrate a unique approach to transfer‐hydrogenative C−C bond formation, wherein the native reducing power of an alcohol released upon either lactonization or lactamization is used to drive catalytic turnover. This approach provides an interesting example of an atom‐economical methodology, highlighting how an otherwise wasted byproduct can be used productively. The studies described herein encompass the first catalytic methods for accessing lactones and lactams by the direct reductive coupling of carbonyls and imines, respectively, with unfunctionalized acrylates. Future studies will seek to identify other catalyst systems which can promote the stereocontrolled coupling of a wider range of reaction partners.

## Conflict of interest

D. C. B. is an employee of and stakeholder in Pfizer Inc.

## Supporting information

As a service to our authors and readers, this journal provides supporting information supplied by the authors. Such materials are peer reviewed and may be re‐organized for online delivery, but are not copy‐edited or typeset. Technical support issues arising from supporting information (other than missing files) should be addressed to the authors.

Supplementary
