# Supplementary material for: Reductive Coupling of Acrylates with Ketones and Ketimines by a Nickel‐Catalyzed Transfer‐Hydrogenative Strategy
Source: Angew Chem Int Ed Engl. 2017 Oct 4;56(44):13824–8. doi: 10.1002/anie.201707531 (PMC5656909; doi:10.1002/anie.201707531)
Supplement: Supplementary file 1 — Supplementary [file ANIE-56-13824-s001.pdf]

Angewandte Chemie
Eine Zeitschrift der Gesellschaft Deutscher Chemiker

Supporting Information

Reductive Coupling of Acrylates with Ketones and Ketimines by a Nickel-Catalyzed Transfer-Hydrogenative Strategy

*Craig S. Buxton, David C. Blakemore, and John F. Bower**

anie_201707531_sm_miscellaneous_information.pdf

Contents

General Experimental Details 1

General Procedures 2

Synthesis of Isatin Substrates 3

Synthesis of Acrylates 9

Spirolactonization of Isatins 11

Lactonization of Non-Isatin derived 1,2-Dicarbonyls 24

Mechanistic Studies 26

Synthesis of Ketimines 29

Spirolactamization of Ketimines 34

One-Pot Imine Formation-Lactamization 40

NMR Spectra for Novel Compounds 41

References 64

General Experimental Details

All reactions involving air-sensitive reagents were performed under a dry nitrogen atmosphere. Anhydrous toluene was obtained by passage through a column of anhydrous alumina using equipment from Anhydrous Engineering, based on the Grubbs' design. $Ni(cod)_2$ was purchased from Sigma Aldrich, stored in a glovebox and used as supplied. Removal of solvents *in vacuo* was achieved using a Büchi rotary evaporator with bath temperatures up to 55 °C. Flash column chromatography (FCC) was carried out using Fluorochem 60 silica: 230-400 mesh (40-63 μm). Reactions were monitored by thin layer chromatography (TLC) analysis using Merck Kiseselgel $60F_{254}$ aluminium backed plates. Spots were visualized by UV light (λ_{max} = 254 nm) and were stained with $KMnO_4$.

^1H, ^{13}C and ^{19}F NMR spectra were recorded on a 400 MHz JEOL Eclipse 400, Varian 400-MR spectrometer, Varian VNMR S500a, Varian VNMR S500b and Bruker cryo500. NMR spectra were referenced to residual solvent signal. Chemical shifts are quoted in parts per million (ppm) and coupling constants (*J*) are quoted to the nearest 0.5 Hz. Multiplicities are reported as singlet (*s*), doublet (*d*), triplet (*t*), quartet (*q*), pentet (*p*) and board singlet (*br. s*). Assignments were based upon multiplicities, COSY, nOe, HSQC and HMBC experiments. *In situ* calculations were performed with 1,4-dinitrobenzene as internal standard.

Infrared spectroscopy was performed on a Perkin Elmer Spectrum Two FTIR spectrometer recorded in the range 4000-600 cm^{-1} as thin films or solids on a diamond plate.

Mass spectra were recorded using electrospray ionization (ESI^+), performed on a Bruker Daltonics microTOF II, chemical ionization (CI^+) and electronic ionization (EI^+) were performed on a VG Autospec at 70 eV. Methane was used as the reagent gas for CI ionization.

General Procedures

General Procedure 1 – Benzylation of Isatin Derivatives

To an MeCN solution (0.10 M) of isatin (1.00 equiv.) was added K_2CO_3 (3.00 equiv.) and benzyl bromide (1.50 equiv.) at room temperature. The mixture was heated at reflux overnight. The mixture was cooled, filtered and concentrated *in vacuo*. The residue was purified by recrystallization or FCC, under the conditions noted, to afford the title compound.

General Procedure 2 – Formation of benzyl acrylates from acryloyl chlorides

To acryloyl chloride (1.00 equiv.) in CH_2Cl_2 (0.50 M) was added benzyl alcohol (1.00 equiv.) and Et_3N (2.00 equiv.) at 0 °C. The reaction was stirred overnight, and then quenched with aqueous 1 M HCl. The phases were separated and the aqueous phase was extracted with CH_2Cl_2 (× 2). The combined organic extracts were dried over Na_2SO_4 and concentrated *in vacuo*. The resultant residue was purified by FCC, under the conditions noted, to afford the product.

General Procedure 3 – Formation of benzyl acrylates from acrylic acids

To a suspension of the acrylic acid (1.05 equiv.) and K_2CO_3 (0.58 equiv.) in DMF (1.65 M) was added benzyl bromide (1.00 equiv.) and the reaction was stirred at room temperature overnight. The reaction was filtered and concentrated *in vacuo* (under high vacuum to remove DMF). The residue was dissolved in EtOAc and washed with saturated aqueous $NaHCO_3$ (× 3). The organic extracts were dried over Na_2SO_4, filtered, concentrated *in vacuo*. The residue was dissolved in EtOAc and filtered through a plug of silica. Concentration of the filtrate afforded product, which was used in the next step without further purification.

General Procedure 4 – Ni-catalyzed spirocyclization of 1,2-dicarbonyls

A re-sealable tube fitted with a Young's tap was charged with the appropriate 1,2-dicarbonyl (100 mol%), P(*o*-OMeC_6H_4)$_3$ (15.0 mol%) and Mg(OTf)$_2$ (10 mol%, *if stated*). The vessel was transferred into a glovebox where Ni(cod)$_2$ (7.5 mol%) was added. The vessel was sealed and transferred to a Schlenk line where benzyl alcohol (10 mol%) and PhMe (0.20 M for isatins, 0.30 M for benzils, freeze–pump thawed) were added. The mixture was stirred for 1 minute and then freshly distilled benzyl acrylate (3 equiv.) was added. The mixture was heated at 150 °C overnight and then cooled to room temperature. The mixture was

concentrated *in vacuo* and the residue was purified by FCC, under the conditions noted, to afford the product.

General Procedure 5 – Formation of imine substrates

To the appropriate 1,2-dicarbonyl (1.00 equiv.) in ethanol (0.20 M) was added *p*-anisidine (1.20 equiv.) and acetic acid (0.05 mL). The reaction mixture was then heated at reflux for 2 hours. The reaction was cooled to room temperature and the mixture was concentrated *in vacuo*. The resultant solid was purified by FCC, under the conditions noted, to afford the title compound.

Synthesis of Isatin Substrates

1-Benzylindoline-2,3-dione (1a)

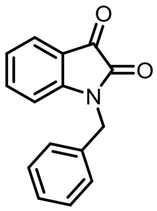

General Procedure 1 – Isatin (3.01 g, 20.4 mmol), K_2CO_3 (8.53 g, 61.7 mmol) and benzyl bromide (3.40 mL, 28.6 mmol) were used. Purification by recrystallization (PhMe) afforded the title compound **1a** (4.77 g, 99%) as an orange crystalline solid.

^{1}H NMR (400 MHz, $CDCl_3$): 4.93 (*s*, 2H), 6.78 (*d*, 1H, *J* = 8.0 Hz), 7.09 (*dd*, 1H, *J* = 7.5, 7.5 Hz), 7.28-7.38 (*m*, 5H), 7.48 (*dd*, 1H, *J* = 8.0, 7.5 Hz), 7.61 (*d*, 1H, *J* = 7.5 Hz).

^{13}C NMR (100 MHz, $CDCl_3$): 44.2, 111.1, 117.9, 124.0, 125.6, 127.6, 128.3, 129.2, 134.7 138.4, 150.9, 158.4, 183.4.

M.P.: 129-131 °C (EtOH). Lit. 129-130 °C (EtOH).[1]

The data are consistent with the literature values.[2]

1-Benzyl-5-methoxyindoline-2,3-dione (1d)

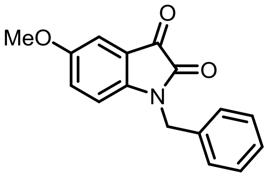

General Procedure 1 – 5-Methoxy isatin (0.51 g, 2.88 mmol), K_2CO_3 (1.19 g, 8.61 mmol) and benzyl bromide (0.50 mL, 4.20 mmol) were used. Purification by recrystallization (PhMe) afforded the title compound **1d** (0.64 g, 84%) as a burgundy solid.

^{1}H NMR (400 MHz, $CDCl_3$): 3.74 (*s*, 3H), 4.87 (*s*, 2H), 6.66 (*d*, 1H, *J* = 8.5 Hz), 7.00 (*dd*, 1H, *J* = 8.5, 2.5 Hz), 7.11 (*d*, 1H, *J* = 2.5 Hz), 7.24-7.34 (*m*, 5H).

^{13}C NMR (126 MHz, $CDCl_3$): 44.1, 56.0, 109.7, 112.1, 118.2, 124.7, 127.5, 128.2, 129.1, 134.7, 144.64, 156.6, 158.4, 183.7.

M.P.: 120-121 °C (PhMe). Lit. 122-124 °C (*no solvent quoted*).[3]

The data are consistent with the literature values.[4]

1-Benzyl-5-(trifluoromethoxy)indoline-2,3-dione (1e)

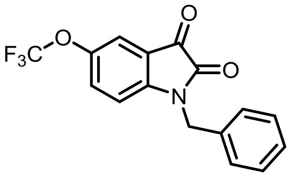

General Procedure 1 – 5-Trifluoromethoxy isatin (199 mg, 0.86 mmol), K_2CO_3 (368 mg, 2.66 mmol) and benzyl bromide (0.16 mL, 1.35 mmol) were used. Purification by recrystallization (PhMe/hexane) afforded the title compound **1e** (168 mg, 61%) as an orange solid.

^{1}H NMR (500 MHz, $CDCl_3$): 4.95 (*s*, 2H), 6.80 (*d*, 1H, *J* = 8.5 Hz), 7.31-7.39 (*m*, 6H), 7.48-7.49 (*dd*, 1H, *J* = 2.5, 1.0 Hz).

^{13}C NMR (126 MHz, $CDCl_3$): 44.4, 112.2, 118.3, 118.7, 120.5 (*q*, 1C, $J_{C\text{-}F}$ = 285.5 Hz), 127.6, 128.6, 129.4, 131.1, 134.1, 145.5 (*q*, 1C, $J_{C\text{-}F}$ = 2.0 Hz), 149.2, 158.1, 182.4.

^{19}F NMR (377 MHz, $CDCl_3$): -58.4 (*s*, 3F)

M.P.: 106-107 °C (PhMe/Hexane). No melting point has been reported in the literature.

The data are consistent with the literature values.[5]

1-Benzyl-7-fluoroinoline-2,3-dione (1f)

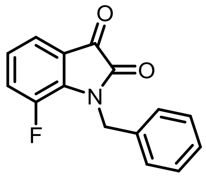

General Procedure 1 – 7-Fluoro isatin (201 mg, 1.22 mmol), K_2CO_3 (503 mg, 3.64 mmol) and benzyl bromide (0.22 mL, 1.85 mmol) were used. Purification by recrystallization (PhMe/hexane) afforded the title compound **1f** (266 mg, 85%) as an orange solid.

^{1}H NMR (500 MHz, $CDCl_3$): 5.06 (*s*, 2H), 7.06 (*ddd*, 1H, J = 8.5, 7.5, 4.0 Hz), 7.26-7.38 (*m*, 6H), 7.44 (*dd*, 1H, J = 7.5, 1.0 Hz).

^{13}C NMR (126 MHz, $CDCl_3$): 46.1 (*d*, 1C, $J_{C\text{-}F}$ = 4.5 Hz), 120.5 (*d*, 1C, $J_{C\text{-}F}$ = 2.5 Hz), 121.6 (*d*, 1C, $J_{C\text{-}F}$ = 3.5 Hz), 124.9 (*d*, 1C, $J_{C\text{-}F}$ = 6.0 Hz), 126.7 (*d*, 1C, $J_{C\text{-}F}$ = 20.0 Hz), 127.9 (*d*, 1C, $J_{C\text{-}F}$ = 2.0 Hz), 128.3, 129.0, 135.8, 136.8 (*d*, 1C, $J_{C\text{-}F}$ = 9.0 Hz), 148.0 (*d*, 1C, J = 248.6 Hz), 158.2, 182.5 (*d*, 1C, $J_{C\text{-}F}$ = 3.5 Hz).

^{19}F NMR (377 MHz, $CDCl_3$): -130.7 (*dd*, 1F, $J_{F\text{-}H}$ = 11.0, 4.0 Hz).

M.P.: 154-157 °C (EtOH). Lit. 155-156 °C (EtOH).[6]

The data are consistent with the literature values.[6]

1-Benzyl-7-(trifluoromethyl)inoline-2,3-dione (1g)

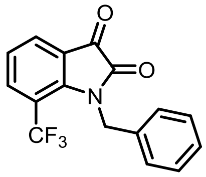

General Procedure 1 – 7-Trifluoromethyl isatin (1.01 g, 4.67 mmol), K_2CO_3 (1.99 g, 14.4 mmol) and benzyl bromide (0.83 mL, 6.98 mmol) were used. Purification by recrystallization (PhMe/hexane) afforded the title compound **1g** (1.20 g, 84%) as an orange solid.

^{1}H NMR (500 MHz, $CDCl_3$): 5.16 (*s*, 2H), 7.09-7.11 (*m*, 2H), 7.16-7.26 (*m*, 4H), 7.79-7.82 (*m*, 2H).

^{13}C NMR (126 MHz, $CDCl_3$): 46.3, 115.0 (*q*, 1C, *J* = 34.0 Hz), 120.3, 122.6 (*q*, 1C, *J* = 272.5 Hz), 123.8, 125.9, 127.5, 128.7, 129.1, 135.0 (*q*, 1C, *J* = 1.0 Hz), 136.0 (*q*, 1C, *J* = 6.0 Hz), 148.8 (*q*, 1C, *J* = 2.0 Hz), 159.4, 181.6.

^{19}F NMR (377 MHz, $CDCl_3$): -55.5 (*s*, 3F).

M.P.: 91-93 °C (PhMe/Hexane). Lit. 86-88 °C (*no solvent quoted*).[7]

The data are consistent with the literature values.[8]

Benzyl 1-benzyl-2,3-dioxoindoline-7-carboxylate (1h)

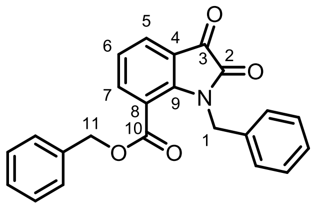

General Procedure 1 – 5-Trifluoromethoxy isatin (199 mg, 0.86 mmol), K_2CO_3 (368 mg, 2.66 mmol) and benzyl bromide (0.16 mL, 1.35 mmol) were used. Purification by FCC (hexane:EtOAc, 4:1) afforded the title compound **1h** (277 mg, 56%) as an orange solid.

^{1}H NMR (500 MHz, $CDCl_3$): 5.05 (*s*, 2H, **H11**), 5.32 (*s*, 2H, **H1**), 7.06-7.11 (*m*, 3H, **H6** and **ArH**), 7.23-7.26 (*m*, 5H, **ArH**), 7.36-7.38 (*m*, 3H, **ArH**), 7.68 (*dd*, 1H, *J* = 8.0, 1.5 Hz, **H7**), 7.76 (*dd*, 1H, *J* = 7.5, 1.5 Hz, **H5**).

^{13}C NMR (126 MHz, $CDCl_3$): 45.5 (**C1**), 68.0 (**C11**), 119.1 (**C8**), 119.7 (**C4**), 123.4 (**C6**), 127.4 (**ArC**), 127.9 (**ArC**), 128.1 (**C5**), 128.7 (**ArC**), 128.8 (2×C, **ArC**), 129.0 (**ArC**), 134.4 (**ArC**), 134.8 (**ArC**), 139.0 (**C7**), 148.8 (**C9**), 159.3 (**C2**), 165.1 (**C10**), 182.4 (**3**).

ν_{max} / cm^{-1} (neat): 3035, 2940, 1744, 1707, 1594, 1442, 1141.

m/z (ESI^+): $C_{23}H_{17}NO_4Na$ $[M+Na]^+$ requires 394.1050, found 394.1044.

M.P.: 147-149 °C (CH_2Cl_2).

1-Benzyl-7-methylinoline-2,3-dione (1i)

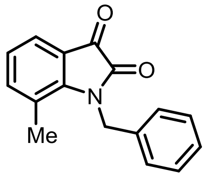

General Procedure 1 – 7-Methyl isatin (201 mg, 1.25 mmol), K_2CO_3 (530 mg, 3.84 mmol) and benzyl bromide (0.22 mL, 1.85 mmol) were used. Purification by FCC (PhMe:EtOAc, 20:1) afforded the title compound **1i** (172 mg, 55%) as an orange solid.

^{1}H NMR (400 MHz, $CDCl_3$): 2.25 (*s*, 3H), 5.19 (*s*, 2H), 7.01 (*dd*, 1H, *J* = 7.5, 7.5 Hz), 7.19-7.21 (*m*, 2H), 7.24-7.29 (*m*, 2H), 7.31-7.35 (*m*, 2H), 7.51 (*dd*, 1H, *J* = 7.5, 1.5 Hz).

^{13}C NMR (100 MHz, $CDCl_3$): 18.7, 45.4, 118.9, 122.1, 123.6, 124.1, 125.8, 127.7, 129.2, 136.4, 142.6, 148.7, 159.7, 183.7.

M.P.: 173-176 °C (CH_2Cl_2). Lit. 174-175 °C (ethanol).[9]

The data are consistent with the literature values.[9]

Synthesis of isatin 1j

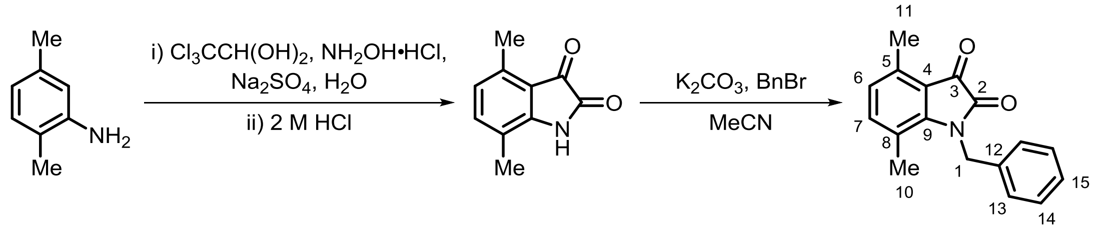

1j

4,7-Dimethylindoline-2,3-dione

Chloral hydrate (7.33 g, 44.3 mmol) was added to a mixture of hydroxylamine hydrogen chloride (10.2 g, 147 mmol), Na_2SO_4 (45.3 g, 319 mmol) and 2,5-dimethyl aniline (5.00 mL, 40.1 mmol) in H_2O (260 mL). To this mixture was added aqueous 2 M HCl (13.4 mL), the mixture was heated at 55 °C for 18 hours. After this time, the reaction was cooled to room temperature and extracted in EtOAc (150 mL × 3), washed with brine (200 mL), dried over Na_2SO_4, filtered and concentrated *in vacuo*. The residue was added portion wise to a solution of concentrated H_2SO_4 (16.0 mL) and H_2O (3.40 mL) at 0 °C. Care was taken to ensure the temperature did not exceed 60 °C during the addition. When all the solids were dissolved the

reaction was heated at 80 °C and stirred for 1 hour. The reaction was cooled to room temperature, poured onto ice and filtered. The crude material was dissolved in DMF (200 mL) and to this solution was added excess K_2CO_3. The solution was then stirred at 60 °C for 30 minutes. The solution was then filtered hot and concentrated *in vacuo* to afford isatin (4.58 g, 65%) as an orange solid. *The product was used in the next step without further purification*.

^{1}H NMR (400 MHz, DMF-d_7): 2.24 (*s*, 3H), 2.45 (*s*, 3H), 6.82 (*d*, 1H, *J* = 8.0 Hz), 7.33 (*d*, 1H, *J* = 8.0 Hz), 10.98 (*br. s*, 1H).

^{13}C NMR (101 MHz, DMF-d_7): 15.07, 17.09, 116.3, 119.2, 125.0, 137.5, 139.5, 149.8, 160.1, 185.7.

M.P.: >210 °C (MeCN). Lit. 274-276 °C (*no solvent quoted*).[10]

The data are consistent with the literature values.[10]

1-Benzyl-4,7-dimethylindoline-2,3-dione (1j)

General Procedure 1 – 4,7-Dimethylindoline-2,3-dione (1.00 g, 5.69 mmol), K_2CO_3 (2.37 g, 17.2 mmol) and benzyl bromide (1.02 mL, 8.58 mmol) were used. Purification by FCC (hexane:EtOAc, 5:1) afforded the benzylated isatin **1j** (265 mg, 18%) as an orange solid.

^{1}H NMR (500 MHz, $CDCl_3$): 2.22 (*s*, 3H, **H10**), 2.58 (*s*, 3H, **H11**), 5.20 (*s*, 2H, **H1**), 6.80 (*d*, 1H, *J* = 8.0 Hz, **H6**), 7.11 (*d*, 1H, *J* = 8.0 Hz, **H7**), 7.20-7.22 (*m*, 2H, **H13**), 7.25-7.30 (*m*, 1H, **H15**), 7.32-7.36 (*m*, 2H, **H14**).

^{13}C NMR (126 MHz, $CDCl_3$): 18.1 (**C11**), 18.6 (**C10**), 45.4 (**C1**), 116.9 (**C5**), 119.1 (**C8**), 125.8 (**C13**), 126.6 (**C6**), 127.7 (**C15**), 129.1 (**C14**), 136.5 (**C12**), 139.5 (**C4**), 142.0 (**C7**), 148.6 (**C9**), 159.6 (**C2**), 182.2 (**C3**).

ν_{max} / cm^{-1} (neat): 3028, 2925, 1721, 1575, 1496, 1334.

m/z (ESI^+): $C_{17}H_{16}NO_2$ $[M+H]^+$ requires 266.1176, found 266.1187.

M.P.: 180-182 °C (CH_2Cl_2).

Synthesis of Acrylates

Benzyl methacrylate (2c)

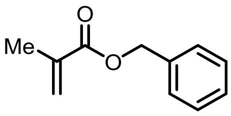

General Procedure 2 – Methacryloyl chloride (1.00 mL, 10.3 mmol), benzyl alcohol (1.07 mL, 10.3 mmol) and Et_3N (2.90 mL, 20.6 mmol) were used. Purification by FCC (hexane:EtOAc, 30:1) afforded the title product **2c** (1.13 g, 62%) as a colorless solid.

^{1}H NMR (400 MHz, $CDCl_3$): 1.98 (*dd*, 3H, *J* = 1.5, 1.0 Hz), 5.20 (*s*, 2H), 5.59 (*dq*, 1H, *J* = 1.5, 1.5 Hz), 6.16 (*dq*, 1H, 1.5, 1.0 Hz), 7.30-7.39 (*m*, 5H).

^{13}C NMR (101 MHz, $CDCl_3$): 18.5, 66.5, 125.9, 128.2, 128.3, 128.7, 136.3, 136.4, 167.4.

The data are consistent with the literature values.[11]

Benzyl 2-Phenylacrylate (2d)

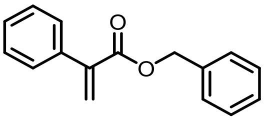

General Procedure 3 – 2-Phenylacrylic acid (1.00 g, 6.78 mmol), K_2CO_3 (0.519 g, 3.75 mmol) and benzyl bromide (0.77 mL, 6.47 mmol) were used. Filtration through silica afforded the title compound **2d** (1.56 g, quantitative) as a colorless oil.

^{1}H NMR (400 MHz, $CDCl_3$): 5.29 (*s*, 2H), 5.93 (*d*, 1H, *J* = 1.0 Hz), 6.40 (*d*, 1H, *J* = 1.0 Hz), 7.31-7.44 (*m*, 10H).

^{13}C NMR (101 MHz, $CDCl_3$): 66.9, 127.2, 128.2, 128.3 (2×C), 128.4, 128.5, 128.7, 136.1, 136.8, 141.4, 166.7.

The data are consistent with the literature values.[12]

Benzyl crotonate (2e)

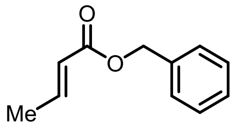

General Procedure 3 – Crotonic acid (2.02 g, 23.5 mmol), K_2CO_3 (1.81 g, 13.1 mmol) and benzyl bromide (2.63 mL, 21.86 mmol) were used. Filtration through silica afforded the title compound **2e** (3.41 g, 89%) as a colorless oil.

^{1}H NMR (400 MHz, $CDCl_3$): 1.89 (*dd*, 3H, *J* = 7.0, 1.5 Hz), 5.18 (*s*, 2H), 5.90 (*dq*, 1H, *J* = 15.5, 1.5 Hz), 7.03 (*dq*, 1H, *J* = 15.5, 7.0 Hz), 7.30-7.39 (*m*, 5H).

^{13}C NMR (101 MHz, $CDCl_3$): 18.1, 66.1, 122.6, 128.3, 128.7, 136.3, 145.3 (2×C), 166.4.

The data are consistent with the literature values.[13]

Dibenzyl fumarate (2f)

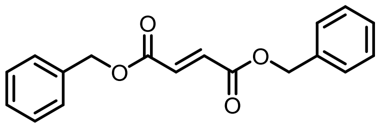

To a DMF (50.0 mL) solution of fumaric acid (2.50 g, 21.5 mmol) was added Et_3N (6.00 mL, 43.0 mmol) and benzyl bromide (4.87 mL, 40.9 mmol), and the mixture was stirred at 100 °C overnight. The mixture was then cooled to room temperature and concentrated *in vacuo*. The residue was dissolved into EtOAc (50 mL) and washed sequentially with aqueous 1 M HCl (30 mL) and saturated aqueous $NaHCO_3$ (30 mL × 2). The organic phase was dried over Na_2SO_4, filtered and concentrated *in vacuo*. The residue was purified by recrystallization (EtOAc/hex) to afford the title compound **2f** (1.67 g, 26%) as an off-white solid.

^{1}H NMR (400 MHz, $CDCl_3$): 5.26 (*s*, 4H), 6.95 (*s*, 2H), 7.35-7.43 (*m*, 10H).

^{13}C NMR (101 MHz, $CDCl_3$): 64.2, 128.5, 128.7, 128.8, 133.9, 135.3, 164.8.

M.P.: 59-61 °C (EtOAc/hex). Lit. 59-60 °C (EtOH).[14]

The data are consistent with the literature values.[14-15]

Spirolactonization of Isatins

1’-Benzyl-3,4-dihydro-5*H*-spiro[furan-2,3’-indoline]2’,5-dione (3a)

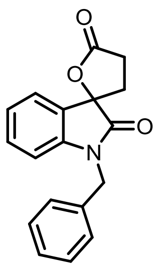

General Procedure 4 – 1-Benzylindoline-2,3-dione (39.2 mg, 0.165 mmol), P(*o*-OMeC_6H_4)$_3$ (9.10 mg, 15.0 mol%), Ni(cod)$_2$ (3.50 mg, 7.50 mol%), benzyl alcohol (1.75 µL, 10 mol%), and benzyl acrylate **2a** (78 µL, 0.508 mmol) were used. Purification by FCC (hexane:EtOAc, 3:1) afforded spirolactone **3a** (40.5 mg, 84%) as a colorless solid.

^{1}H NMR (400 MHz, $CDCl_3$): 2.47 (*ddd*, 1H, *J* = 13.5, 11.0, 9.5 Hz), 2.60 (*ddd*, 1H, *J* = 13.5, 9.5, 3.0 Hz), 2.77 (*ddd*, 1H, *J* = 17.5, 9.5, 3.0 Hz), 3.22 (*ddd*, 1H, *J* = 17.5, 11.0, 9.5 Hz), 4.85 (*s*, 2H), 6.71 (*d*, 1H, *J* = 7.5 Hz), 7.06 (*dd*, 1H, *J* = 7.5, 7.5 Hz), 7.22-7.34 (*m*, 7H).

^{13}C NMR (100 MHz, $CDCl_3$): 28.5, 31.6, 44.0, 82.4, 110.0, 123.8, 124.5, 126.5, 127.4, 128.1, 129.1, 131.3, 135.0, 143.2, 174.5, 176.2.

M.P.: 117-120 °C (EtOAc/hexane). Lit. 117-118 (EtOAc).[16]

The data are consistent with the literature values.[17]

1’-Methyl-3,4-dihydro-5*H*-spiro[furan-2,3-indoline]-2’,5-dione (3b)

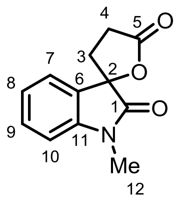

General Procedure 4 – 1-Methylindoline-2,3-dione **1b** (27.3 mg, 0.165 mmol), P(*o*-OMeC_6H_4)$_3$ (9.30 mg, 15.0 mol%), Ni(cod)$_2$ (3.50 mg, 7.50 mol%), benzyl alcohol (1.75 µL, 10 mol%), and benzyl acrylate **2b** (78 µL, 0.508 mmol) were used. Purification by FCC (PhMe:EtOAc, 10:1) afforded spirolactone **3b** (26.3 mg, 73%) as an colorless solid.

^{1}H NMR (400 MHz, $CDCl_3$): 2.45 (*ddd*, 1H, *J* = 13.5, 10.5, 9.5 Hz, **H3**), 2.57 (*ddd*, 1H, *J* = 13.5, 9.5, 3.0 Hz, **H3**), 2.76 (*ddd*, 1H, *J* = 17.5, 9.5, 3.0 Hz, **H4**), 3.14-3.27 (*m*, 1H, **H4**), 3.19 (*s*, 3H, **H12**), 6.86 (*dd*, 1H, *J* = 8.0, 1.0 Hz, **H10**), 7.13 (*ddd*, 1H, *J* = 7.5, 7.5, 1.0 Hz, **H8**), 7.35 (*dd*, 1H, *J* = 7.5, 1.0, Hz, **H7**), 7.40 (*ddd*, 1H, *J* = 8.0, 7.5, 1.0 Hz, **H9**).

^{13}C NMR (100 MHz, $CDCl_3$): 26.5 (**C12**), 28.4 (**C4**), 31.4 (**C3**), 82.4 (**C2**), 109.0 (**C10**), 123.7 (**C8**), 124.3 (**C7**), 126.5 (**C6**), 131.3 (**C9**), 144.0 (**C11**), 174.3 (**C1**), 176.2 (**C5**).

ν_{max} / cm^{-1} (neat): 3063, 2987, 1782, 1719, 1616, 1470.

m/z (ESI^+): $C_{12}H_{11}NO_3Na$ $[M+Na]^+$ requires 240.0631, found 240.0638.

M.P.: 121-124 °C (CH_2Cl_2).

1'-Phenyl-3,4-dihydro-5*H*-spiro[furan-2,3-indoline]-2',5-dione (3c)

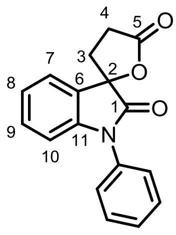

General Procedure 4 – 1-Phenylindoline-2,3-dione **1c** (37.1 mg, 0.166 mmol), $P(o\text{-}OMeC_6H_4)_3$ (9.10 mg, 15.0 mol%), $Ni(cod)_2$ (3.50 mg, 7.50 mol%), benzyl alcohol (1.75 µL, 10 mol%), and benzyl acrylate **2b** (78 µL, 0.508 mmol) were used. Purification by FCC (hexane:EtOAc, 3:1) afforded spirolactone **3c** (36.3 mg, 78%) as a peachy crystalline solid.

^{1}H NMR (400 MHz, $CDCl_3$): 2.56 (*ddd*, 1H, *J* = 13.5, 10.5, 9.5 Hz, **H3**), 2.72 (*ddd*, 1H, *J* = 13.5, 9.5, 3.0 Hz, **H3**), 2.81 (*ddd*, 1H, *J* = 17.5, 9.5, 3.0 Hz, **H4**), 3.23 (*ddd*, 1H, *J* = 17.5, 10.5, 9.5 Hz, **H4**), 6.85 (*ddd*, 1H, *J* = 8.0, 1.0, 1.0 Hz, **H10**), 7.17 (*ddd*, 1H, *J* = 7.5, 7.5, 1.0 Hz, **H8**), 7.33 (*ddd*, 1H, *J* = 8.0, 7.5, 1.5 Hz, **H9**), 7.40-7.45 (*m*, 4H, **ArH** and **H7**), 7.51-7.55 (*m*, 2H, **ArH**).

^{13}C NMR (100 MHz, $CDCl_3$): 28.4 (**C4**), 31.8 (**C3**), 82.5 (**C2**), 110.3 (**C10**), 124.2 (**C8**), 124.7 (**C7**), 126.2 (**C6**), 126.4 (**ArC**), 128.7 (**ArC**), 129.9 (**ArC**), 131.2 (**C9**), 133.5 (**ArC**), 144.1 (**C11**), 173.7 (**C1**), 176.1 (**C5**)

ν_{max} / cm^{-1} (neat): 3065, 2988, 1783, 1729, 1614, 1499.

m/z (ESI$^+$): $C_{17}H_{13}NO_3Na$ [M+Na]$^+$ requires 302.0788, found 302.0783.

M.P.: 127-129 °C (CH_2Cl_2).

1’-Benzyl-5’-methoxy-3,4-dihydro-5*H*-spiro[furan-2,3’-indoline]-2’,5-dione (3d)

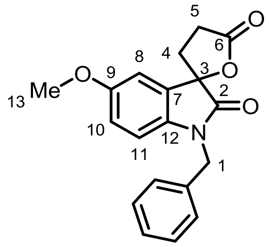

General Procedure 4 – 1-Benzyl-5-methoxyindoline-2,3-dione **1d** (47.2 mg, 0.177 mmol), P(*o*-OMeC_6H_4)$_3$ (9.10 mg, 15.0 mol%), Ni(cod)$_2$ (3.50 mg, 7.50 mol%), benzyl alcohol (1.75 µL, 10 mol%), and benzyl acrylate **2b** (78 µL, 0.508 mmol) were used. Purification by FCC (hexane:EtOAc, 3:1) afforded spirolactone **3d** (48.4 mg, 85%) as a colorless solid.

^{1}H NMR (400 MHz, $CDCl_3$): 2.48 (*ddd*, 1H, *J* = 13.0, 11.0, 9.5 Hz, **H4**), 2.63 (*ddd*, 1H, *J* = 13.0, 9.5, 3.0 Hz, **H4**), 2.79 (*ddd*, 1H, *J* = 17.5, 9.5, 3.0 Hz, **H5**), 3.24 (*ddd*, 1H, *J* = 17.5, 11.0, 9.5 Hz, **H5**), 3.75 (*s*, 3H, **H13**), 4.85 (*s*, 2H, **H1**), 6.63 (*d*, 1H, *J* = 8.5 Hz, **H11**), 6.78 (*dd*, 1H, *J* = 8.5, 2.5 Hz, **H10**), 6.97 (*d*, 1H, *J* = 2.5 Hz, **H8**), 7.26-7.34 (*m*, 5H, **ArH**).

^{13}C NMR (100 MHz, $CDCl_3$): 28.4 (**C5**), 31.7 (**C4**), 44.0 (**C1**), 56.0 (**C13**), 82.7 (**C3**), 110.6 (**C11**), 111.4 (**C8**), 115.8 (**C10**), 127.3 (**ArC**), 127.6 (**C7**), 128.0 (**ArC**), 129.0 (**ArC**), 135.1 (**ArC**), 136.2 (**C12**), 156.8 (**C9**), 174.3 (**C2**), 176.2 (**C6**).

ν_{max} / cm^{-1} (neat): 3063, 2971, 1785, 1716, 1603, 1494, 1435, 1172.

m/z (ESI$^+$): $C_{19}H_{18}NO_4$ [M+H]$^+$ requires 324.1230, found 324.1243.

M.P.: 160-162 °C ($CHCl_3$).

The structure of this compound was determined unambiguously by X-ray crystallography.

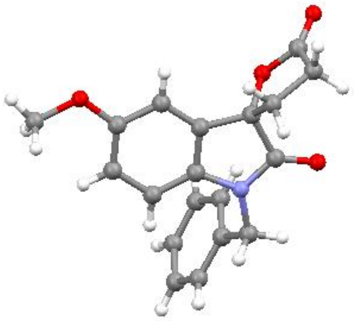

1'-Benzyl-5'-(trifluoromethoxy)-3,4-dihydro-5*H*-spiro[furan-2,3'-indoline]-2',5-dione (3e)

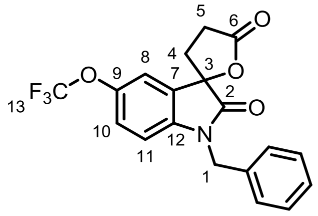

General Procedure 4 – 1-Benzyl-5-(trifluoromethoxy)indoline-2,3-dione **1e** (56.2 mg, 0.175 mmol), P(*o*-OMeC_6H_4)$_3$ (9.10 mg, 15.0 mol%), Ni(cod)$_2$ (3.50 mg, 7.50 mol%), benzyl alcohol (1.75 μL, 10 mol%), and benzyl acrylate **2b** (78 μL, 0.508 mmol) were used. Purification by FCC (hexane:EtOAc, 4:1) afforded spirolactone **3e** (33.1 mg, 50%) as a pale brown oil.

^{1}H NMR (500 MHz, $CDCl_3$): 2.50 (*ddd*, 1H, *J* = 13.5, 11.0, 9.5 Hz, **H4**), 2.66 (*ddd*, 1H, *J* = 13.5, 9.5, 2.5 Hz, **H4**), 2.81 (*ddd*, 1H, *J* = 17.5, 9.5, 2.5 Hz, **H5**), 3.26 (*ddd*, 1H, *J* = 17.5, 11.0, 9.5 Hz, **H5**), 4.88 (*s*, 2H, **H1**), 6.73 (*d*, 1H, *J* = 8.5 Hz, **H11**), 7.13-7.16 (*m*, 1H, **H10**), 7.26-7.37 (*m*, 6H, **H8** and **ArH**).

^{13}C NMR (126 MHz, $CDCl_3$): 28.2 (**C5**), 31.6 (**C4**), 44.2 (**C1**), 81.9 (**C3**), 110.8 (**C11**), 117.5 (**C8**), 120.5 (*q*, 1C, *J* = 257.5 Hz, **C13**), 124.4 (**C10**), 127.4 (**ArC**), 127.9 (**C7**), 128.3 (**ArC**), 129.2 (**ArC**), 134.5 (**ArC**), 141.8 (**C12**), 145.5 (*q*, 1C, *J* = 2.0 Hz, **C9**), 174.4 (**C2**), 175.6 (**C6**).

^{19}F NMR (377 MHz, $CDCl_3$): -58.26 (*s*, 3F).

ν_{max} / cm^{-1} (neat): 3066, 2915, 1794, 1728, 1492, 1250.

m/z (ESI$^+$): $C_{19}H_{14}NO_4F_3Na$ [M+Na]$^+$ requires 400.0767, found 400.0769.

1’-Benzyl-7’-fluoro-3,4-dihydro-5*H*-spiro[furan-2,3’-indoline]-2’,5-dione (3f)

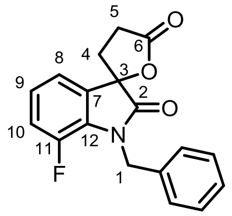

General Procedure 4 – 1-Benzyl-7-fluoroindoline-2,3-dione **1f** (42.5 mg, 0.167 mmol), P(*o*-OMeC_6H_4)$_3$ (9.20 mg, 15.0 mol%), Ni(cod)$_2$ (3.50 mg, 7.50 mol%), benzyl alcohol (1.75 μL, 10 mol%), and benzyl acrylate **2b** (78 μL, 0.508 mmol) were used. Purification by FCC (hexane:EtOAc, 3:1) afforded spirolactone **3f** (41.4 mg, 80%) as an off-white solid.

^{1}H NMR (400 MHz, $CDCl_3$): 2.46 (*ddd*, 1H, *J* = 13.5, 11.0, 9.5 Hz, **H4**), 2.62 (*ddd*, 1H, *J* = 13.5, 9.5, 3.0 Hz, **H4**), 2.79 (*ddd*, 1H, *J* = 17.5, 9.5, 3.0 Hz, **H5**), 3.22 (*ddd*, 1H, *J* = 17.5, 11.0, 9.5 Hz, **H5**), 4.98 (*dd*, 1H, *J* = 15.5, 1.0 Hz, **H1**), 5.04 (*dd*, 1H, *J* = 15.0, 1.0 Hz, **H1**), 7.03-7.08 (*m*, 2H, **H9** and **H10**), 7.13-7.18 (*m*, 1H, **H8**), 7.25-7.34 (*m*, 5H, **ArH**).

^{13}C NMR (100 MHz, $CDCl_3$): 28.2 (**C5**), 31.8 (**C4**), 45.6 (*d*, 1C, $J_{C\text{-}F}$ = 5.0 Hz, **C1**), 82.1 (*d*, 1C, $J_{C\text{-}F}$ = 2.5 Hz, **C3**), 119.4 (*d*, 1C, $J_{C\text{-}F}$ = 20.0 Hz, **C10**), 120.4 (*d*, 1C, $J_{C\text{-}F}$ = 3.5 Hz, **C8**), 124.7 (*d*, 1C, $J_{C\text{-}F}$ = 6.5 Hz, **C9**), 127.6 (*d*, 1C, $J_{C\text{-}F}$ = 2.0 Hz, **ArC**), 128.0 (**ArC**), 128.9 (**ArC**), 129.4 (*d*, 1C, $J_{C\text{-}F}$ = 3.0 Hz, **C7**), 129.7 (*d*, 1C, $J_{C\text{-}F}$ = 9.0 Hz, **C12**), 136.2 (**ArC**), 147.6 (*d*, 1C, *J* = 245.8 Hz, **C11**), 174.3 (**C2**), 175.8 (**C6**).

^{19}F NMR (377 MHz, $CDCl_3$): -132.29 - -132.35 (*m*, 1F).

ν_{max} / cm^{-1} (neat): 2987, 1789, 1727, 1633, 1475, 1350, 1167.

m/z (ESI$^+$): $C_{18}H_{15}NO_3F$ [M+H]$^+$ requires 312.1031, found 312.1036.

M.P.: 139-140 °C (CH_2Cl_2).

1'-Benzyl-7'-(trifluoromethyl)-3,4-dihydro-5*H*-spiro[furan-2,3'-indoline]-2',5-dione (3g)

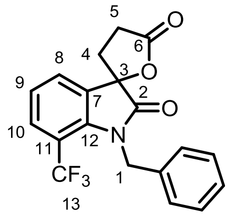

General Procedure 4 – 1-Benzyl-7-(trifluoromethyl)indoline-2,3-dione **1g** (51.2 mg, 0.168 mmol), P(*o*-OMeC_6H_4)$_3$ (9.10 mg, 15.0 mol%), Ni(cod)$_2$ (3.50 mg, 7.50 mol%), benzyl alcohol (1.75 µL, 10 mol%), and benzyl acrylate **2b** (78 µL, 0.508 mmol) were used. Purification by FCC (hexane:EtOAc, 3:1) afforded spirolactone **3g** (24.7 mg, 57%) as an off-white solid.

^{1}H NMR (400 MHz, $CDCl_3$): 2.50 (*ddd*, 1H, *J* = 13.5, 11.0, 9.5 Hz, **H5**), 2.64 (*ddd*, 1H, *J* = 13.5, 9.5, 2.5 Hz, **H5**), 2.81 (*ddd*, 1H, *J* = 17.5, 9.5, 2.5 Hz, **H4**), 3.24 (*ddd*, 1H, *J* = 17.5, 11.0, 9.5 Hz, **H4**), 5.12 (*d*, 1H, *J* = 17.0 Hz, **H1**), 5.22 (*d*, 1H, *J* = 17.0 Hz, **H1**), 7.04-7.13 (*m*, 2H, **ArH**), 7.21-7.31 (*m*, 4H, **ArH** and **H9**), 7.60 (*dd*, 1H, *J* = 7.5, 1.5 Hz, **H8**), 7.66 (*dd*, 1H, *J* = 8.0, 1.5 Hz, **H10**).

^{13}C NMR (126 MHz, $CDCl_3$): 28.3 (**C4**), 32.0 (**C5**), 45.7 (*q*, 1C, *J* = 5.0 Hz, **C1**), 80.2 (**C3**), 114.0 (*q*, 1C, *J* = 33.5 Hz, **C11**), 123.0 (*q*, 1C, *J* = 272.0 Hz, **C13**), 123.5 (**C9**), 125.7 (**ArC**), 127.4 (**ArC**), 128.3 (**C8**), 128.7 (**ArC**), 129.4 (*q*, 1C, *J* = 6.5 Hz, **C10**), 129.4 (**C7**) 135.5 (**ArC**), 141.6 (**C12**), 175.6 (**C6**), 175.9 (**C2**).

^{19}F NMR (377 MHz, $CDCl_3$): -55.1 (*s*, 3F).

ν_{max} / cm^{-1} (neat): 3032, 2935, 1796, 1737, 1602, 1512, 1336, 1161.

m/z (ESI$^+$): $C_{19}H_{14}NO_3F_3Na$ [M+Na]$^+$ requires 384.0718, found 384.0814.

M.P.: 130-131 °C (EtOAc/hex).

Benzyl 1’-benzyl-2’,5-dioxo-4,5-dihydro-3*H*-spiro[furan-2,3’-indoline]-7’carboxylate (3h)

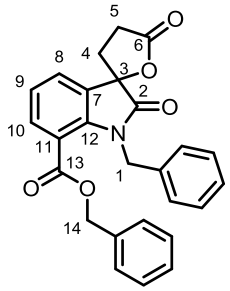

General Procedure 4 – Benzyl 1-benzyl-2,3-dioxoindoline-7-carboxylate **1h** (62.9 mg, 0.169 mmol), P(*o*-OMeC_6H_4)$_3$ (8.90 mg, 15.0 mol%), Ni(cod)$_2$ (3.50 mg, 7.50 mol%), benzyl alcohol (1.75 μL, 10 mol%), and benzyl acrylate **2b** (78 μL, 0.508 mmol) were used. Purification by FCC (hexane:EtOAc, 3:1) afforded spirolactone **3h** (43.3 mg, 60%) as a colorless solid.

^{1}H NMR (400 MHz, $CDCl_3$): 2.50 (*ddd*, 1H, *J* = 13.0, 11.0, 9.5 Hz, **H4**), 2.67 (*ddd*, 1H, *J* = 13.0, 9.5, 2.5 Hz, **H4**), 2.82 (*ddd*, 17.5, 9.5, 2.5 Hz, **H5**), 3.27 (*ddd*, 1H, *J* = 17.5, 11.0, 9.5 Hz, **H5**), 4.98 (*d*, 1H, *J* = 12.0 Hz, **H14**), 5.09 (*d*, 1H, *J* = 16.0 Hz, **H1**), 5.11 (*d*, 1H, *J* = 12.0 Hz, **H14**), 5.43 (*d*, 1H, *J* = 16.0 Hz, **H1**), 7.00-7.02 (*m*, 2H, **ArH**), 7.09 (*dd*, 1H, *J* = 7.5, 7.5 Hz, **H9**), 7.20-7.26 (*m*, 5H, **ArH**), 7.34-7.36 (*m*, 3H, **ArH**), 7.48-7.50 (*m*, 2H, **H8** and **H10**).

^{13}C NMR (100 MHz, $CDCl_3$): 28.3 (**C5**), 31.8 (**C4**), 45.2 (**C1**), 67.7 (**C14**), 81.1 (**C3**), 118.0 (**C11**), 123.1 (**C9**), 127.0 (**ArC**), 127.2 (**C8**), 127.6 (**ArC**), 128.5 (**ArC**), 128.6 (**C7**), 128.7 (2×C, **ArC**), 128.8 (**ArC**), 132.2 (**C10**), 134.6 (**ArC**), 135.0 (**ArC**), 141.3 (**C12**), 165.7 (**C13**), 175.8 (**C2**), 175.9 (**C6**).

*Differentiation of **C8** and **C10** in the ^{13}C NMR was made by analogy to other similar compounds.*

ν_{max} / cm^{-1} (neat): 3032, 2950, 1790, 1730, 1603, 1454, 1255, 1134.

m/z (ESI^+): $C_{26}H_{21}NO_5Na$ $[M+Na]^+$ requires 450.1312, found 450.1327.

M.P.: 143-145 °C (CH_2Cl_2).

1’-Benzyl-7’-methyl-3,4-dihydro-5*H*-spiro[furan-2,3’-indoline]-2’,5-dione (3i)

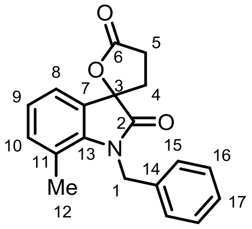

General Procedure 4 – 1-Benzyl-7-methylindoline-2,3-dione **1i** (43.5 mg, 0.173 mmol), P(*o*-OMe$C_6H_4)_3$ (9.00 mg, 15.0 mol%), Ni(cod)$_2$ (3.50 mg, 7.50 mol%), benzyl alcohol (1.75 μL, 10 mol%), and benzyl acrylate **2b** (78 μL, 0.508 mmol) were used. Purification by FCC (hexane:EtOAc, 3:1) afforded spirolactone **3i** (40.3 mg, 76%) as an orange solid.

^{1}H NMR (400 MHz, $CDCl_3$): 2.26 (*s*, 3H, **H12**), 2.51 (*ddd*, 1H, *J* = 13.0, 11.0, 9.5 Hz, **H4**), 2.64 (*ddd*, 1H, *J* = 13.0, 9.5, 3.0 Hz, **H4**), 2.80 (*ddd*, 1H, *J* = 17.5, 9.5, 3.0 Hz, **H5**), 3.24 (*ddd*, 1H, *J* = 17.5, 11.0, 9.5 Hz, **H5**), 5.10 (*d*, 1H, *J* = 17.0 Hz, **H1**), 5.20 (*d*, 1H, *J* = 17.0 Hz, **H1**), 7.01-7.06 (*m*, 2H, **H10** and **H8**), 7.14-7.16 (*m*, 2H, **H15**), 7.24-7.28 (*m*, 2H, **H17** and **H9**), 7.31-7.35 (*m*, 2H, **H16**).

^{13}C NMR (100 MHz, $CDCl_3$): 18.7 (**C12**), 28.6 (**C5**), 32.0 (**C4**), 45.2 (**C1**), 82.0 (**C3**), 120.9 (**C11**), 122.4 (**C9**), 123.9 (**C8**), 125.7 (**C15**), 127.2 (**C7**), 127.6 (**C17**), 129.1 (**C16**), 135.1 (**C10**), 136.7 (**C14**), 141.1 (**C13**), 175.7 (**C2**), 176.3 (**C6**).

N_{max} / cm^{-1} (neat): 3032, 2975, 1785, 1719, 1605, 1445, 1355, 1166.

m/z (ESI^+): $C_{19}H_{18}NO_3$ $[M+H]^+$ requires 308.1281, found 308.1276.

M.P.: 111-113 °C (CH_2Cl_2).

1’-Benzyl-4’,7’-dimethyl-3,4-dihydro-5*H*-spiro[furan-2,3’-indoline]-2’,5-dione (3j)

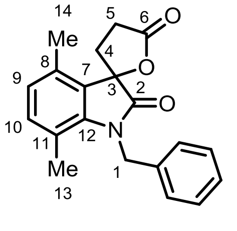

General Procedure 4 – 1-Benzyl-4,7-dimethylindolin-2,3-dione **1j** (57.1 mg, 0.167 mmol), P(*o*-OMe$C_6H_4)_3$ (9.20 mg, 15.0 mol%), Ni(cod)$_2$ (3.50 mg, 7.50 mol%), benzyl alcohol

(1.75 μL, 10 mol%), and benzyl acrylate **2b** (78 μL, 0.508 mmol) were used. Purification by FCC (hexane:EtOAc, 3:1) afforded spirolactone **3j** (45.1 mg, 68%) as a colorless solid.

^{1}H NMR (400 MHz, $CDCl_3$): 2.22 (*s*, 3H, **H13**), 2.34 (*s*, 3H, **H14**), 2.55 (*ddd*, 1H, *J* = 13.0, 10.5, 2.0 Hz, **H4**), 2.69 (*ddd*, 1H, *J* = 13.0, 10.5, 10.0 Hz, **H4**), 2.78 (*ddd*, 1H, *J* = 17.0, 10.0, 2.0 Hz, **H5**), 3.27 (*ddd*, 1H, *J* = 17.0, 10.5, 10.5 Hz, **H5**), 5.10 (*d*, 1H, *J* = 17.0 Hz, **H1**), 5.17 (*d*, 1H, *J* = 17.0 Hz, **H1**), 6.79 (*d*, 1H, *J* = 8.0 Hz, **H9**), 6.93 (*d*, 1H, *J* = 8.0 Hz, **H10**), 7.12-7.14 (*m*, 2H, **ArC**), 7.23-7.34 (*m*, 3H, **ArC**).

^{13}C NMR (100 MHz, $CDCl_3$): 17.4 (**C14**), 18.6 (**C13**), 28.1 (**C5**), 29.4 (**C4**), 45.2 (**C1**), 82.7 (**C3**), 118.3 (**C11**), 124.3 (**C8**), 125.7 (**ArC**), 126.2 (**C9**), 127.6 (**ArC**), 129.1 (**ArC**), 134.0 (**C7**), 134.9 (**C10**), 136.8 (**ArC**), 141.1 (**C12**), 176.0 (**C2**), 176.5 (**C6**).

N_{max} / cm^{-1} (neat): 2969, 1784, 1720, 1592, 1452, 1407, 1348.

m/z (ESI^+): $C_{20}H_{20}NO_3$ $[M+H]^+$ requires 322.1438, found 322.1425.

M.P.: 162-164 °C (CH_2Cl_2).

1’-Benzyl-4-methyl-3,4-dihydro-5*H*-spiro[furan-2,3’-indoline]-2’,5-dione (3k)

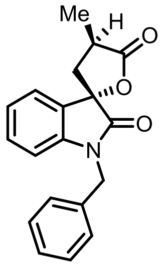

General Procedure 4 – 1-Benzylindoline-2,3-dione **1a** (39.9 mg, 0.168 mmol), P(*o*-$OMeC_6H_4)_3$ (9.10 mg, 15.0 mol%), $Mg(OTf)_2$ (5.50 mg, 10 mol%), $Ni(cod)_2$ (3.50 mg, 7.50 mol%), benzyl alcohol (1.75 μL, 10 mol%), and benzyl 2-methylacrylate **2c** (186 mg, 1.06 mmol) were used. Purification by FCC (hexane:EtOAc, 5:1) afforded spirolactone **3k** (38.8 mg, 75%, >20:1 d.r.) as a colorless solid.

^{1}H NMR (400 MHz, $CDCl_3$): 1.54 (*d*, 3H, *J* = 7.0 Hz), 2.46 (*dd*, 1H, *J* = 13.0, 9.5 Hz), 2.63 (*dd*, 1H, *J* = 13.0, 9.5 Hz), 3.15 (*ddq*, 1H, *J* = 9.5, 9.5, 7.0 Hz), 4.87 (*d*, 1H, *J* = 15.5 Hz), 4.94 (*d*, 1H, *J* = 15.5 Hz), 6.75 (*d*, 1H, *J* = 8.0 Hz), 7.06 (*dd*, 1H, *J* = 7.5, 7.5 Hz), 7.24-7.35 (*m*, 7H).

^{13}C NMR (100 MHz, $CDCl_3$): 16.6, 34.9, 38.6, 44.3, 80.8, 110.2, 123.7, 123.8, 127.4, 127.9, 128.1, 129.1, 131.0, 135.0, 142.7, 174.0, 178.6.

M.P.: 127-128 °C (CH_2Cl_2/hex). No melting point has been reported in the literature.

The structure and relative stereochemistry of this compound were determined unambiguously by X-ray crystallography.

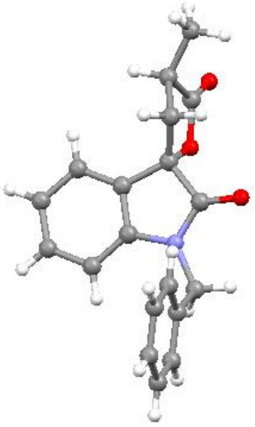

The data are consistent with the literature values.[17]

1'-Benzyl-4-phenyl-3,4-dihydro-5*H*-spiro[furan-2,3'-indoline]-2',5-dione (3l)

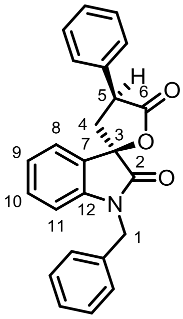

General Procedure 4 – 1-Benzylindoline-2,3-dione **1a** (39.9 mg, 0.168 mmol), P(*o*-OMeC_6H_4)$_3$ (9.10 mg, 15.0 mol%), Mg(OTf)$_2$ (5.50 mg, 10 mol%), Ni(cod)$_2$ (3.50 mg, 7.50 mol%), benzyl alcohol (1.75 μL, 10 mol%), and benzyl 2-phenylacrylate **2d** (126 mg, 0.529 mmol) were used. Purification by FCC (hexane:EtOAc, 10:1) afforded spirolactone **3l** (38.8 mg, 63%, >20:1 d.r.) as a colorless solid.

^{1}H NMR (400 MHz, $CDCl_3$): 2.71 (*dd*, 1H, *J* = 13.5, 12.5 Hz, **H4**), 3.03 (*dd*, 1H, *J* = 13.5, 9.0 Hz, **H4**), 4.79 (*dd*, 1H, *J* = 12.5, 9.0 Hz, **H5**), 4.89 (*s*, 1H, *J* = 15.5 Hz, **H1**), 4.94 (*d*, 1H, *J*

= 15.5 Hz, **H1**), 6.78 (*d*, 1H, *J* = 8.0 Hz, **H11**), 7.13 (*dd*, 1H, *J* = 7.5, 7.5 Hz, **H9**), 7.28-7.45 (*m*, 12H, **ArH**, **H8** and **H10**).

^{13}C NMR (100 MHz, $CDCl_3$): 40.7 (**C4**), 44.1 (**C1**), 45.5 (**C5**), 80.4 (**C3**), 110.1 (**C11**), 123.9 (**C9**), 124.6 (**C8**), 125.9 (**C7**), 127.4 (**ArC**), 128.1 (2×C, **ArC**), 128.3 (**ArC**), 129.1 (**ArC**), 129.2 (**ArC**), 131.4 (**C10**), 135.0 (**ArC**), 136.4 (**ArC**), 143.4 (**C12**), 174.5 (**C2**), 176.2 (**C6**).

ν_{max} / cm^{-1} (neat): 3031, 2926, 1785, 1721, 1616, 1490, 1468, 1370.

m/z (ESI$^+$): $C_{24}H_{19}NO_3$ [M+H]$^+$ requires 370.1438, found 370.1435.

M.P.: 156-158 °C (CH_2Cl_2).

The structure and relative stereochemistry of this compound were determined unambiguously by X-ray crystallography.

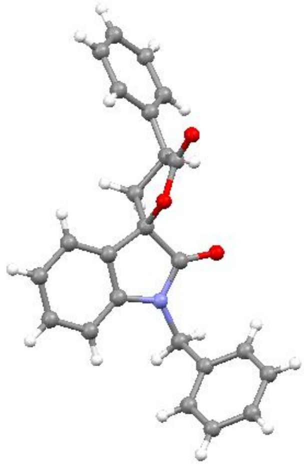

1’-Benzyl-3-methyl-3,4-dihydro-5*H*-spiro[furan-2,3’-indoline]-2’,5-dione (3m and 3m’)

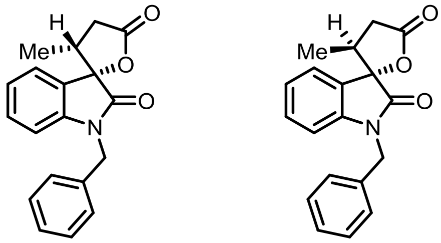

3m (**A**) *major diastereomer*

3m' (**B**) *minor diastereomer*

General Procedure 4 – 1-Benzylindoline-2,3-dione **1a** (40.5 mg, 0.171 mmol), P(*o*-OMeC_6H_4)$_3$ (8.90 mg, 15.0 mol%), $Mg(OTf)_2$ (5.50 mg, 10 mol%), $Ni(cod)_2$ (3.50 mg,

7.50 mol%), benzyl alcohol (1.75 μL, 10 mol%), and benzyl 3-methylacrylate **3e** (184 mg, 1.04 mmol) were used. Purification by FCC (hexane:EtOAc, 4:1) afforded spirolactones **3m** and **3m'** (40.4 mg, 77%, as a 0.83:0.17 mixture of diastereomers **3m**(**A**): **3m'**(**B**)) as a colorless solid.

^{1}H NMR (400 MHz, $CDCl_3$): 1.01 (*d*, 2.49H, *J* = 7.0 Hz, **A**), 1.03 (*d*, 0.51H, *J* = 7.0 Hz, **B**), 2.50 (*dd*, 0.83H, *J* = 17.5, 7.5 Hz, **A**), 2.73 (*dd*, 0.17H, *J* = 16.0, 7.5 Hz, **B**), 2.96-3.05 (*m*, 1H, **A** and **B**), 3.00-3.08 (*m*, 0.17H, **B**), 3.24 (*dd*, 0.83H, *J* = 17.5, 8.5 Hz, **A**), 4.72 (*d*, 0.17H, *J* = 15.5 Hz, **B**), 4.79 (*d*, 0.83H, *J* = 15.5 Hz, **A**), 4.95 (*d*, 0.83H, *J* = 15.5 Hz, **A**), 4.98 (*d*, 0.17H, *J* = 15.5 Hz, **B**), 6.71 (*d*, 0.17H, *J* = 8.0 Hz, **B**), 6.74 (*d*, 0.83H, *J* = 8.0 Hz, **A**), 7.04 (*dd*, 0.83H, *J* = 7.5, 7.5 Hz, **A**), 7.07 (*dd*, 0.17H, *J* = 7.5, 7.5 Hz, **B**), 7.23-7.32 (*m*, 7H, **A** and **B**).

^{13}C NMR (100 MHz, $CDCl_3$): 13.0 (**B**), 16.3 (**A**), 35.2 (**B**), 36.0 (**A**), 37.4 (**A**), 40.5 (**B**), 44.2 (**B**), 44.2 (**A**), 85.9 (**A**), 86.2 (**B**), 109.8 (**B**), 110.2 (**A**), 123.2 (**A**), 123.7 (**B**), 124.1 (**A**), 124.5 (**B**), 124.9 (**B**), 125.8 (**A**), 127.3 (**A**), 127.4 (**B**), 128.0 (**B**), 128.1 (**A**), 129.0 (**B**), 129.1 (**A**), 131.0 (**A**), 131.2 (**B**), 135.1 (**A**), 135.2 (**B**), 143.2 (**A**), 143.9 (**B**), 173.3 (**B**), 174.4 (**A**), 175.5 (**A**), 175.6 (**B**).

M.P.: *Diastereomer A* - 146-148 °C (CH_2Cl_2/hex). Lit. 146-147 °C (*no solvent quoted*).[17]

*The structure and relative stereochemistry of **3m(A)** were determined unambiguously by X-ray crystallography*.

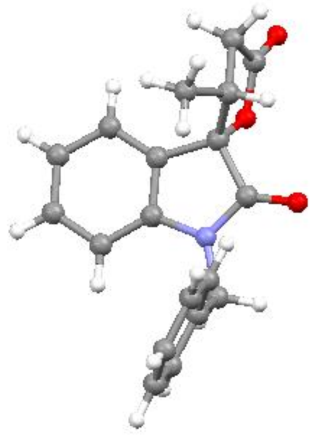

Diastereomer A - The data are consistent with the literature values.[17]

Diastereomer B - The data are consistent with the literature values.[18]

Benzyl 1'-Benzyl-2',5-dioxo-4,5-dihydro-3*H*-spiro[furan-2,3'-indoline]-3-carboxylate (3n)

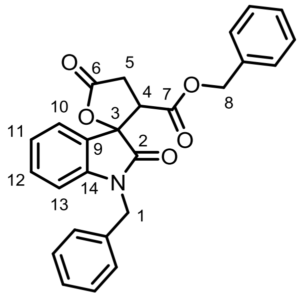

General Procedure 4 – 1-Benzylindoline-2,3-dione **1a** (39.8 mg, 0.168 mmol), P(*o*-OMeC_6H_4)$_3$ (9.00 mg, 15.0 mol%), Ni(cod)$_2$ (3.50 mg, 7.50 mol%), benzyl alcohol (1.75 μL, 10 mol%), and dibenzyl fumarate **2f** (151 mg, 0.510 mmol) were used. Purification by FCC (hexane:EtOAc, 3:1) afforded spirolactone **3n** (52.0 mg, 73% as a 1:1 mixture of diastereomers **A**:**B**) as a pale brown oil.

^{1}H NMR (400 MHz, $CDCl_3$): 3.03 (*dd*, 1H, *J* = 17.5, 9.5 Hz, **A-H5**), 3.13 (*dd*, 1H, *J* = 18.0, 9.5 Hz, **B-H5**), 3.37 (*dd*, 1H, *J* = 18.0, 9.5 Hz, **B-H5**), 3.65 (*dd*, 1H, *J* = 17.5, 12.0 Hz, **A-H5**), 3.91 (*dd*, 1H, *J* = 12.0, 9.5 Hz, **A-H4**), 3.98 (*dd*, 1H, *J* = 9.5, 9.5 Hz, **B-H4**), 4.41 (*d*, 1H, *J* = 16.0 Hz, **A-H1**), 4.64 (*d*, 1H, *J* = 16.0 Hz, **B-H1**), 4.68 (*d*, 1H, *J* = 12.0 Hz, **B-H8**), 4.74 (*d*, 1H, *J* = 16.0 Hz, **A-H1**), 4.79 (*d*, 1H, *J* = 12.0 Hz, **B-H8**), 4.83 (*d*, 1H, *J* = 16.0 Hz, **B-H1**), 4.98 (*s*, 2H, **A-H8**), 6.61 (*d*, 1H, *J* = 8.0 Hz, **B-H13**), 6.63 (*d*, 1H, *J* = 8.0 Hz, **A-H13**), 6.87-6.90 (*m*, 2H, **ArH**), 6.95 (*dd*, 1H, *J* = 7.5, 7.5 Hz, **B-H11**), 7.05-7.06 (*m*, 2H, **ArH**), 7.10 (*dd*, 1H, *J* = 7.5, 7.5 Hz, **A-H11**), 7.16-7.38 (*m*, 18H, **ArH**, **A-H10**, **B-H10**, **A-H12**, **B-H12**).

^{13}C NMR (100 MHz, $CDCl_3$): 30.6 (**B-C5**), 31.2 (**A-C5**), 43.8 (**A-C1**), 44.3 (**B-C1**), 47.2 (**B-C4**), 48.3 (**A-C4**), 67.5 (**A-C8**), 67.6 (**B-C8**), 82.3 (2×C, **A-C3** and **B-C3**), 110.2 (2×C, **A-C13** and **B-C13**), 123.5 (2×C, **A-C11** and **B-C11**), 123.7 (**B-C9**), 124.4 (**A-C10**), 124.5 (**B-C10**), 124.9 (**A-C9**), 127.3 (**B-ArC**), 127.4 (**A-ArC**), 127.9 (**ArC**), 128.0 (**ArC**), 128.3 (**ArC**), 128.6 (4×C, **ArC**), 128.7 (**ArC**), 128.9 (**ArC**), 129.0 (**ArC**), 131.5 (2×C, **A-C12** and **B-C12**), 134.4 (**ArC**), 134.6 (**ArC**), 134.7 (**ArC**), 134.9 (**ArC**), 143.1 (**B-C14**), 144.2 (**A-**

C14), 167.7 (**A-C7**), 168.4 (**B-C7**), 172.7 (**B-C2**), 172.8 (**A-C2**), 173.3 (**A-C6**), 173.5 (**B-C6**).

ν_{max} / cm^{-1} (neat): 3032, 2928, 1800, 1728, 1615, 1468, 1370, 1180.

m/z (ESI^+): $C_{26}H_{22}NO_5$ $[M+H]^+$ requires 428.1493, found 428.1474.

Lactonization of Non-Isatin derived 1,2-Dicarbonyls

5-(4-Fluorobenzoyl)-5-(4-fluorophenyl)dihydrofuran-2(3*H*)-one (5a)

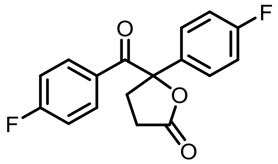

General Procedure 4 – 4,4’-Difluorobenzil **4a** (41.5 mg, 0.169 mmol), P(*o*-OMeC_6H_4)$_3$ (9.30 mg, 15.0 mol%), Ni(cod)$_2$ (3.50 mg, 7.50 mol%), benzyl alcohol (1.75 μL, 10 mol%), and benzyl acrylate **2b** (78 μL, 0.508 mmol) were used and heated at 140 °C. Purification by FCC (PhMe) afforded lactone **5a** (36.4 mg, 71%) as a colorless oil.

^{1}H NMR (400 MHz, $CDCl_3$): 2.29 (*ddd*, 1H, *J* = 13.0, 8.5, 8.5 Hz), 2.51-2.64 (*m*, 2H), 3.42 (*ddd*, 1H, 13.0, 8.5, 7.0 Hz), 6.99-7.05 (*m*, 2H), 7.06-7.12 (*m*, 2H), 7.41-7.46 (*m*, 2H), 7.97-8.02 (*m*, 2H).

^{13}C NMR (100 MHz, $CDCl_3$): 28.1, 34.5 (*d*, 1C, *J* = 1.0 Hz), 91.8, 115.8 (*d*, 1C, *J* = 22.0 Hz), 116.6 (*d*, 1C, *J* = 22.0 Hz), 125.8 (*d*, 1C, *J* = 8.0 Hz), 129.8 (*d*, 1C, *J* = 3.0 Hz), 133.7 (*d*, 1C, *J* = 9.5 Hz), 135.2 (*d*, 1C, *J* = 3.0 Hz), 162.9 (*d*, 1C, *J* = 248.5 Hz), 166.0 (*d*, 1C, *J* = 257.0 Hz), 175.3, 193.7.

^{19}F NMR (377 MHz, $CDCl_3$): -103.2 - -103.3 (*m*, 1F), -112.5 - -112.6 (*m*, 1F).

The data are consistent with the literature values.[19]

5-Benzoyl-5-phenyldihydrofuran-2(3*H*)-one (5b)

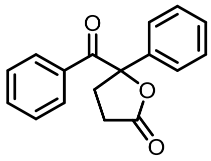

General Procedure 4 – Benzil **4b** (35.6 mg, 0.169 mmol), P(*o*-OMeC_6H_4)$_3$ (9.20 mg, 15.0 mol%), Ni(cod)$_2$ (3.50 mg, 7.50 mol%), benzyl alcohol (1.75 µL, 10 mol%), and benzyl acrylate **2b** (78 µL, 0.508 mmol) were used and heated at 140 °C. Purification by FCC (hexane:EtOAc, 7:1) afforded lactone **5b** (29.8 mg, 66%) as a pale yellow oil.

^{1}H NMR (400 MHz, $CDCl_3$): 2.33 (*ddd*, 1H, *J* = 13.0, 8.5, 8.5 Hz), 2.48-2.66 (*m*, 2H), 3.43 (*ddd*, 1H, *J* = 13.0, 8.5, 7.0 Hz), 7.30-7.35 (*m*, 3H), 7.38-7.41 (*m*, 2H), 7.44-7.49 (*m*, 3H), 7.94-7.96 (*m*, 2H).

^{13}C NMR (100 MHz, $CDCl_3$): 28.2, 34.5, 92.3, 123.9, 128.4, 128.7, 129.4, 130.9, 133.6, 133.7, 139.5, 175.7, 195.4.

The data are consistent with the literature values.[17]

5-(4-Methoxybenzoyl)-5-(4-methoxyphenyl)dihydrofuran-2(3*H*)-one (5c)

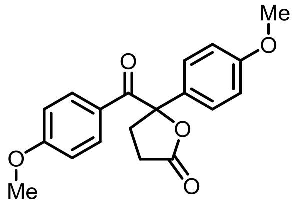

General Procedure 4 – 4,4’-Dimethoxybenzil **4c** (45.0 mg, 0.167 mmol), P(*o*-OMeC_6H_4)$_3$ (8.90 mg, 15.0 mol%), Ni(cod)$_2$ (3.50 mg, 7.50 mol%), benzyl alcohol (1.75 µL, 10 mol%), and benzyl acrylate **2b** (78 µL, 0.508 mmol) were used and heated at 140 °C. Purification by FCC (hexane:EtOAc, 5:1) afforded lactone **5c** (14.6 mg, 27%) as a pale yellow oil.

^{1}H NMR (400 MHz, $CDCl_3$): 2.29 (*ddd*, 1H, *J* = 13.0, 8.5, 8.5 Hz), 2.48-2.61 (*m*, 2H), 3.38 (*ddd*, 1H, *J* = 13.0, 8.5, 7.0 Hz), 3.78 (*s*, 3H), 3.80 (*s*, 3H), 6.79-6.83 (*m*, 2H), 6.87-6.91 (*m*, 2H), 7.35-7.39 (*m*, 2H), 7.95-7.99 (*m*, 2H).

^{13}C NMR (100 MHz, $CDCl_3$): 28.2, 34.5, 55.4, 55.6, 92.3, 113.7, 114.7, 125.3, 126.5, 131.8, 133.4, 159.8, 163.8, 176.0, 193.9.

The data are consistent with the literature values.[19]

3’,4-Dihydro-2*H*,5’*H*-spiro[acenaphthylene-1,2’-furan]-2,5’-dione (7)

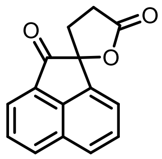

General Procedure 4 – Acenaphthylene-1,2-dione **6** (30.9 mg, 0.169 mmol), P(*o*-OMeC_6H_4)$_3$ (12.1 mg, 20.0 mol%), Mg(OTf)$_2$ (5.50 mg, 10.0 mol%), Ni(cod)$_2$ (4.6 mg, 10.0 mol%), benzyl alcohol (1.75 µL, 10.0 mol%), and benzyl acrylate **2b** (78 µL, 0.508 mmol) were used. Purification by FCC (hexane:EtOAc, 3:1) afforded the title compound **7** (21.1 mg, 52%) as a pale yellow oil.

^{1}H NMR (400 MHz, $CDCl_3$): 2.55-2.68 (*m*, 2H), 2.87 (*ddd*, 1H, *J* = 17.5, 9.0, 4.0 Hz), 3.24 (*ddd*, 1H, *J* = 17.5, 10.5, 9.5 Hz), 7.66 (*d*, 1H, *J* = 7.0 Hz), 7.73 (*dd*, 1H, *J* = 8.5, 7.0 Hz), 7.80 (*dd*, 1H, *J* = 8.0, 7.0 Hz), 7.98 (*d*, 1H, 8.5 Hz), 8.02 (*d*, 1H, *J* = 7.0 Hz), 8.19 (*d*, 1H, *J* = 8.5 Hz).

^{13}C NMR (100 MHz, $CDCl_3$): 28.7, 31.4, 86.1, 121.0, 123.2, 126.8, 128.9, 129.0, 129.7, 130.7, 132.6, 136.2, 142.2, 176.5, 200.3.

The data are consistent with the literature values.[17]

Mechanistic Studies

Benzyl acrylate-*d₂* (*deuterio*-2b)

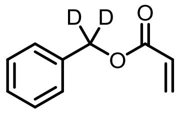

Benzyl alcohol-d_2 (1.08 g, 9.82 mmol) and Et_3N (2.10 mL, 15.1 mmol) in CH_2Cl_2 (8.00 mL) was cooled to 0°C and to it was added acryloyl chloride (0.96 mL, 12.7 mmol) drop wise. The reaction was warmed to room temperature and stirred for 3 hours. After this time, an additional portion of acryloyl chloride (0.37 mL, 4.91 mmol) was added and the mixture was stirred for 1 hour. The reaction was quenched with a saturated aqueous solution of $NaHCO_3$ (15 mL) and then concentrated. The aqueous phase was extracted with EtOAc (20 mL × 3)

and the combined organic extracts were dried over Na_2SO_4 and concentrated *in vacuo*. The residue was dried under high vacuum at room temperature. The residue was purified by distillation (high vacuum) at 110 °C using Hickman still apparatus to afford the title compound (0.84 g, 52%) as a colourless oil.

^{1}H NMR (400 MHz, $CDCl_3$): 5.85 (*dd*, 1H, *J* = 17.5, 1.5 Hz), 6.18 (*dd*¸ 1H, *J* = 17.5, 10.5 Hz), 6.46 (*dd*, 1H, *J* = 17.5, 1.5 Hz), 7.32-7.41 (*m*, 5H).

^{13}C NMR (101 MHz, $CDCl_3$): 65.9 (*p*, 1C, *J* = 22.5 Hz), 128.4 (2×C), 128.5, 128.7, 131.2, 135.9, 166.2.

^{2}H NMR (77 MHz, $CHCl_3$): 5.20 (*s*, 2D).

The data are consistent with the literature values.[20]

1'-Benzyl-3,4-dihydro-5*H*-spiro[furan-2,3'-indoline]-2',5-dione-4-*d* (*deuterio*-3a)

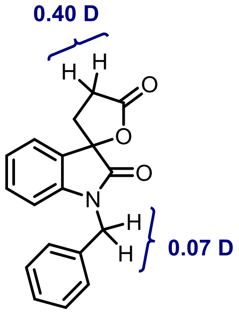

General Procedure 4 – 1-Benzylindoline-2,3-dione **1a** (43.1 mg, 0.182 mmol), P(*o*-OMeC_6H_4)$_3$ (9.00 mg, 15.0 mol%), Ni(cod)$_2$ (3.50 mg, 7.50 mol%), benzyl alcohol (1.75 µL, 10 mol%), and d_2-benzyl acrylate ***deuterio*-2b** (78 µL, 0.508 mmol) were used. Purification by FCC (hexane:EtOAc, 3:1) afforded spirolactone ***deuterio*-3a** (36.5 mg, 68%) as a pale brown oil.

^{1}H NMR (400 MHz, $CDCl_3$): 2.46-2.54 (*m*, 1H), 2.60-2.67 (*m*, 1H), 2.80 (*ddd*, 0.75H, *J* = 17.5, 9.5, 3.0 Hz), 3.20-3.30 (*m*, 0.86H), 4.88 (*s*, 1.93H), 6.75 (*d*, 1H, *J*= 7.5 Hz), 7.10 (*dd*, 1H, *J* = 7.5, 7.5 Hz), 7.25-7.37 (*m*, 7H).

^{13}C NMR (100 MHz, $CDCl_3$): 28.5, 31.6, 44.0, 82.4, 110.0, 123.8, 124.5, 126.5, 127.4, 128.1, 129.1, 131.3, 135.0, 143.2, 174.5, 176.2.

^{2}H NMR (77 MHz, $CHCl_3$): 2.81 (*s*, 0.25D), 3.26 (*s*, 0.15D), 4.95 (*s*, 0.07D)

The data are consistent with the literature values.[17]

1-Benzyl-3-hydroxyindolin-2-one (8)

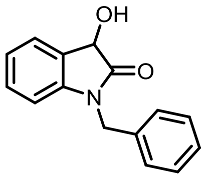

1-Benzylindoline-2,3-dione **1a** (0.51 g, 2.14 mmol) was suspended in methanol (4.30 mL) and cooled to 0 °C. To the suspension was added $NaBH_4$ (0.13 g, 3.44 mmol) portion wise. The reaction was warmed to room temperature and stirred for 30 minutes before the reaction was quenched with H_2O (10 mL) and extracted into CH_2Cl_2 (10 mL × 3). The organic extracts were combined and concentrated *in vacuo*, and the residue was purified by FCC (hexane:EtOAc, 2:1) to afford the title compound **8** (0.34 g, 66%) as a colorless solid.

^{1}H NMR (400 MHz, $CDCl_3$): 3.68 (*d*, 1H, *J* = 4.0 Hz), 4.83 (*d*, 1H, *J* = 15.5 Hz), 4.95 (*d*, 1H, *J* = 15.5 Hz), 5.18 (*d*, 1H, *J* = 4.0 Hz), 6.72 (*d*, 1H, *J* = 8.0 Hz), 7.07 (*dd*, 1H, *J* = 8.0, 7.5 Hz), 7.22 (*dd*, 1H, *J* = 7.5, 7.5 Hz), 7.25-7.34 (*m*, 5H), 7.47 (*d*, 1H, *J* = 7.5 Hz).

^{13}C NMR (100 MHz, $CDCl_3$): 44.0, 70.0, 109.7, 123.4, 125.4, 127.0, 127.5, 127.9, 129.0, 129.9, 135.4, 143.2, 177.1.

M.P.: 138-141 °C (PhMe). Lit. 135-139 °C (PhH).[21]

The data are consistent with the literature values.[22]

Synthesis of Ketimines

(*E*/*Z*)-1-Benzyl-3-((4-methoxyphenyl)imino)indolin-2-one (10a)

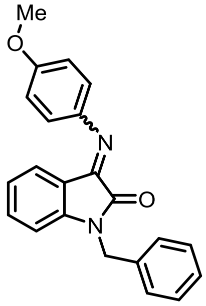

General Procedure 5 – 1-Benzylindoline-2,3-dione **1a** (514 mg, 2.17 mmol), *p*-anisidine (317 mg, 2.57 mmol) and acetic acid (0.05 mL, catalytic) were used. Purification by FCC (hexane:EtOAc, 4:1) afforded the title compound **10a** (660 mg, 87% as a 0.88:0.12 mixture of ketimine isomers **A**:**B**) as a red solid.

^{1}H NMR (500 MHz, $CDCl_3$): 3.84 (*s*, 0.36H), 3.86 (*s*, 2.64H), 4.87 (*s*, 0.24H), 5.01 (*s*, 1.76H), 6.71-6.78 (*m*, 2H), 6.92-6.99 (*m*, 3H), 7.04-7.10 (*m*, 2H), 7.23 (*dd*, 1H, *J* = 8.0, 8.0 Hz,), 7.28-7.39 (*m*, 5H).

^{13}C NMR (126 MHz, $CDCl_3$): 44.1, 55.7, 110.4, 114.7, 116.1, 120.3, 122.7, 125.8, 127.6, 128.0, 129.0, 133.9, 135.3, 143.2, 147.1, 153.8, 158.0, 163.7. *Characteristic signals for the minor isomer: 43.8, 113.8, 123.4, 127.6, 135.6, 145.3.*

The data are consistent with the literature values.[23]

(*E*/*Z*)-1-Benzyl-7-fluoro-((4-methoxyphenyl)imino)indolin-2-one (10b)

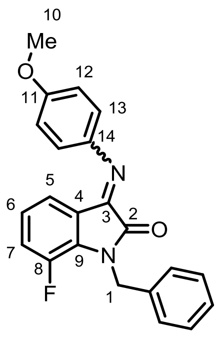

General Procedure 5 – 1-Benzyl-7-fluoroindoline-2,3-dione **1f** (185 mg, 0.723 mmol), *p*-anisidine (107 mg, 0.869 mmol) and acetic acid (0.05 mL, catalytic) were used. Purification

by FCC (hexane:EtOAc, 5:1) afforded the title compound **10b** (236 mg, 91% as a 0.78:0.22 mixture of ketimine isomers **A**:**B**) as a red solid.

^{1}H NMR (500 MHz, $CDCl_3$): 3.85 (*s*, 0.66H, **B-H10**), 3.86 (*s*, 2.34H, **A-H10**), 5.02 (*s*, 0.44H, **B-H1**), 5.16 (*s*, 1.56H, **A-H1**), 6.71 (*ddd*, 0.78H, *J* = 8.5, 7.5, 4.5 Hz, **A-H6**), 6.79 (*dd*, 0.78H, *J* = 7.5, 1.0 Hz, **A-H5**), 6.92-9.95 (*m*, 0.44H, **B-H12**), 6.96-6.99 (*m*, 1.56H, **A-H12**), 7.01-7.06 (*m*, 2.56H, **A-H7**, **A-H13** and **B-H6**), 7.11 (*ddd*, 0.22H, *J* = 11.5, 8.5, 1.0 Hz, **B-H7**), 7.27 (*m*, 3.88H, **ArH** and **B-H13**), 7.42-7.44 (*m*, 1.56H, **ArH**), 7.53 (*dd*, 0.22H, *J* = 7.5, 1.0 Hz, **B-H5**).

^{13}C NMR (126 MHz, $CDCl_3$): 45.6 (*d*, 0.22C, *J* = 4.5 Hz, **B-C1**), 45.9 (*d*, 0.78C, *J* = 5.0, **A-C1**), 55.6 (**B-C10**), 55.7 (**A-C10**), 113.8 (**B-C12**), 114.8 (**A-C12**), 118.7 (*d*, 0.22C, *J* = 3.5 Hz, **B-C5**), 118.8 (*d*, 0.78C, *J* = 3.5 Hz, **A-C4**), 120.3 (**A-C13**), 121.0 (*d*, 0.22C, *J* = 20.0 Hz, **B-C7**), 121.8 (*d*, 0.78C, *J* = 3.5 Hz, **A-C5**), 122.1 (*d*, 0.78C, *J* = 20.0 Hz, **A-C7**), 123.4 (*d*, 0.78C, *J* = 6.0 Hz, **A-C6**), 123.9 (*d*, 0.22C, *J* = 6.0 Hz, **B-C6**), 124.2 (**B-C13**), 127.9 (2×C **ArC**), 128.0 (2×C, **ArC**), 128.8 (2×C, **ArC**), 131.4 (**B-C9**), 133.7 (*d*, 0.78C, *J* = 8.5 Hz, **A-C9**), 136.6 (**ArC**), 136.7 (**ArC**), 140.8 (**B-C14**), 142.9 (**A-C14**), 147.4 (*d*, 0.22C, *J* = 245.0 Hz, **B-C8**), 148.0 (*d*, 0.78C, *J* = 246.0 Hz, **A-C8**), 152.8 (*d*, 0.78C, *J* = 3.0 Hz, **A-C3**), 157.7 (**B-C2**), 158.2 (**A-C11**), 159.2 (**B-C11**), 163.5 (**A-C2**).

Signals for **B-C4** and **B-C3** were not observed by NMR analysis and **B-C9** was observed by HMBC analysis.

^{19}F NMR (470 MHz, $CDCl_3$): -131.6 (*dd*, 0.78F, *J* = 11.0, 4.5 Hz, **A**), -133.8 (*dd*, 0.22F, *J* = 12.0, 4.5 Hz, **B**).

ν_{max} / cm^{-1} (neat): 3069, 1731, 1621, 1498, 1456, 1339, 1240.

m/z (ESI^+): $C_{22}H_{18}N_2O_2F$ $[M+H]^+$ requires 361.1347, found 361.1363.

(*E/Z*)-1-Benzyl-3-((4-methoxyphenyl)imino)-7-(trifluoromethyl)indolin-2-one (10c)

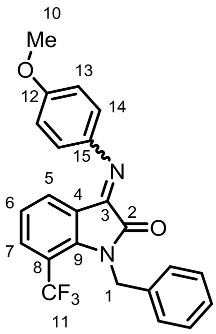

General Procedure 5 – 1-Benzyl-7-(trifluoromethyl)indoline-2,3-dione **1g** (260 mg, 0.851 mmol), *p*-anisidine (127 mg, 1.03 mmol) and acetic acid (0.05 mL, catalytic) were used. Purification by FCC (hexane:EtOAc, 6:1) afforded the title compound **10c** (321 mg, 92%, 1:1 mixture of ketimine isomers ***A***:***B***) as a dark red solid.

^{1}H NMR (500 MHz, $CDCl_3$): 3.84 (*s*, 3H, **B-H10**), 3.88 (*s*, 3H, **A-H10**), 5.19 (*s*, 2H, **A-H1**), 5.32 (*s*, 2H, **B-H1**), 6.88-6.93 (*m*, 3H, **B-H6** and **B-H13**), 6.99-7.02 (*m*, 2H, **A-H14**), 7.03-7.06 (*m*, 2H, **A-H13**), 7.11-7.13 (*m*, 2H, **ArH**), 7.18-7.32 (*m*, 10H, **A-H6**, **B-H5** and **ArH**), 7.45-7.48 (*m*, 2H, **B-H14**), 7.61 (*d*, 1H, *J* = 8.0 Hz, **B-H7**), 7.69 (*d*, 1H, *J* = 8.0 Hz, **A-H7**), 7.99 (*d*, 1H, *J* = 7.5 Hz, **A-H5**).

^{13}C NMR (126 MHz, $CDCl_3$): 45.8 (*q*, 1C, *J* = 5.0 Hz, **A-C1**), 46.2 (*q*, 1C, *J* = 5.0 Hz, **B-C1**), 55.6 (**B-H10**), 55.7 (**A-H10**), 113.4 (*q*, 1C, *J* = 33.0 Hz, **A-C8**), 113.8 (**B-H13**), 114.2 (*q*, 1C, *J* = 33.5 Hz, **B-C8**), 114.9 (**A-C13**), 118.5 (**B-C4**), 120.0 (**A-C14**), 122.3 (**B-C6**), 122.8 (**A-C6**), 123.0 (*q*, 1C, *J* = 272.5 Hz, **B-C11**), 124.4 (*q*, 1C, *J* = 272.0 Hz, **A-C11**), 125.2 (**B-C14**), 125.5 (**A-C4**), 125.9 (**ArC**), 126.0 (**ArC**), 126.2 (**A-C5**), 127.1 (**ArC**), 127.3 (**ArC**), 128.6 (2×C, **ArC**), 128.9 (**B-C5**), 130.5 (*q*, 1C, *J* = 6.0 Hz, **A-C7**), 131.6 (*q*, 1C, *J* = 6.5 Hz, **B-C7**), 135.8 (**ArC**), 136.1 (**ArC**), 140.6 (**B-C15**), 142.8 (2×C, **A-C9** and **A-C15**), 145.4 (*br. q*, 1C, *J* = 1.5 Hz, **B-C9**), 146.9 (**A-C3**), 151.1 (**B-C3**), 158.3 (**A-C12**), 158.8 (**A-C2**), 159.8 (**B-C12**), 164.8 (**B-C2**).

^{19}F NMR (470 MHz, $CDCl_3$): -55.2 (*s*, 3F), -55.3 (*s*, 3F).

ν_{max} / cm^{-1} (neat): 3032, 2938, 1720, 1594, 1499, 1448, 1328, 1246, 1167.

m/z (ESI^+): $C_{23}H_{18}N_2O_2F_3$ $[M+H]^+$ requires 411.1315, found 411.1309.

(*E*/*Z*)-3-((4-Methoxyphenyl)imino)-1-methylindolin-2-one (10d)

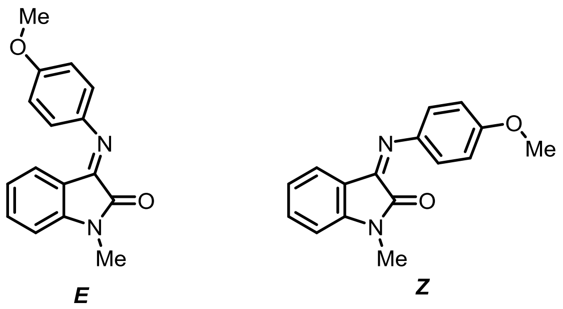

General Procedure 5 – 1-Benzyl-5-methoxyindoline-2,3-dione **1b** (505 mg, 3.13 mmol), *p*-anisidine (476 mg, 3.87 mmol) and acetic acid (0.05 mL, catalytic) were used. Purification by FCC (hexane:EtOAc, 2:1) afforded the title compound **10d** (766 mg, 92%, as a 0.80:0.20 ***E***:***Z*** mixture) as a red solid.

^{1}H NMR (500 MHz, DMSO-d_6): 3.10 (*s*, 0.6H, ***Z***), 3.19 (*s*, 2.4H, ***E***), 3.78 (*s*, 0.6H, ***Z***), 3.80 (*s*, 2.4H, ***E***), 6.69 (*d*, 0.8H, *J* = 7.5 Hz, ***E***), 6.82 (*dd*, 0.8H, *J* = 7.5, 7.5 Hz, ***E***), 6.88-6.92 (*m*, 0.4H, ***Z***), 6.96-7.00 (*m*, 1.6H, ***E***), 7.03-7.12 (*m*, 2.8H, 2×***E*** and 2×***Z***), 7.18-7.22 (*m*, 0.4H, ***Z***), 7.42 (*dd*, 0.8H, *J* = 8.0, 7.5 Hz, ***E***), 7.49 (*dd*, 0.2H, *J* = 8.0, 7.5 Hz, ***Z***), 7.58 (*d*, 0.2H, *J* = 7.5 Hz, ***Z***).

^{13}C NMR (126 MHz, DMSO-*d*): 25.7 (***Z***), 26.0 (***E***), 55.2 (***Z***), 55.3 (***E***), 109.2 (***Z***), 110.0 (***E***), 113.4 (***Z***), 114.7 (***E***), 115.2 (***E***), 119.5 (***E***), 121.4 (***Z***), 121.8 (***Z***), 122.1 (***E***), 122.7 (***Z***), 123.0 (***Z***), 124.5 (***E***), 133.4 (***Z***), 134.0 (***E***), 140.9 (***Z***), 142.9 (***E***), 146.0 (***Z***), 147.7 (***E***), 150.5 (***Z***), 153.7 (***E***), 157.1 (***Z***), 157.2 (***E***), 157.7 (***Z***), 162.3 (***E***).

The data are consistent with the literature values.[24]

(*E/Z*)-1-Benzyl-5-methoxy-3-((4-methoxyphenyl)imino)indolin-2-one (10e)

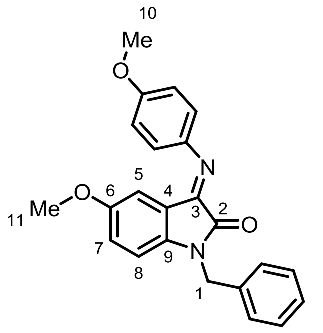

General Procedure 5 – 1-Methylindoline-2,3-dione **1a** (253 mg, 0.948 mmol), *p*-anisidine (142 mg, 1.15 mmol) and acetic acid (0.05 mL, catalytic) were used. Purification by FCC (hexane:EtOAc, 3:1) afforded the title compound **10e** (320 mg, 91%, as a 0.81:0.19 mixture of ketimine isomers **A**:**B**) as a red solid.

^{1}H NMR (500 MHz, $CDCl_3$): 3.52 (*s*, 2.43H, **A-H11**), 3.81 (*s*, 0.57H, **B-H11**), 3.84 (*s*, 0.57H, **B-H10**), 3.85 (*s*, 2.43H, **A-H10**), 4.84 (*s*, 0.38H, **B-H1**), 4.99 (*s*, 1.62H, **A-H1**), 6.55 (*d*, 0.81H, *J* = 2.5 Hz, **A-H5**), 6.61-6.66 (*m*, 1H, **A-H8** and **B-H8**), 6.78 (*dd*, 0.81H, *J* = 8.5, 2.5 Hz, **A-H7**), 6.87 (*dd*, 0.19H, *J* = 8.5, 2.5 Hz, **B-H7**), 6.92-6.95 (*m*, 0.36H, **B-ArH-PMP**), 6.97-6.99 (*m*, 1.62H, **A-ArH-PMP**), 7.05-7.08 (*m*, 1.62H, **A-ArH-PMP**), 7.27-7.37 (*m*, 5.38H, **A-ArH**, **B-ArH**, **B-H5** and **B-ArH-PMP**).

^{13}C NMR (126 MHz, $CDCl_3$): 43.9 (**B-C1**), 44.2 (**A-C1**), 55.6 (**B-C11**), 55.7 (2xC, **A-C11** and **A-C10**), 56.1 (**B-C10**), 107.7 (**B-C5**), 110.5 (**B-C8**), 110.9 (**A-C8**), 112.2 (**A-C5**), 113.8 (**B-ArC-PMP**), 114.7 (**A-ArC-PMP**), 116.6 (**A-C4**), 118.9 (**A-C7**), 119.5 (**B-C7**), 120.4 (**A-ArC-PMP**), 123.2 (**B-C4**), 123.4 (**B-ArC-PMP**), 127.6 (2×C, **A-ArC** and **B-ArC**), 127.9 (**B-ArC**), 128.0 (**A-ArC**), 129.0 (2×C, **A-ArC** and **B-ArC**), 135.4 (**A-ArC**), 135.6 (**B-ArC**), 139.1 (**B-C9**), 140.8 (**A-C9**), 141.2 (**B-ArC-PMP**), 143.0 (**A-ArC-PMP**), 150.7 (**B-C3**), 154.0 (**A-C3**), 155.4 (**A-C6**), 156.4 (**B-C6**), 158.0 (**B-C2**), 158.1 (**A-ArC-PMP**), 158.7 (**B-ArC-PMP**), 163.7 (**A-C2**).

ν_{max} / cm^{-1} (neat): 3022, 2838, 1723, 1590, 1478, 1284, 1242, 1180.

m/z (ESI^+): $C_{23}H_{20}N_2O_3$ $[M+H]^+$ requires 373.1547, found 373.1550.

2-((4-Methoxylphenyl)imino)-1,2-diphenylethan-1-one

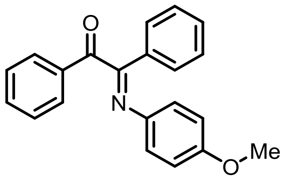

General Procedure 5 – Benzil **4b** (506 mg, 2.41 mmol), *p*-anisidine (357 mg, 2.90 mmol) and acetic acid (0.05 mL, catalytic) were used. Purification by FCC (hexane:EtOAc, 15:1) afforded the title compound (335 mg, 44%) as a yellow solid.

^{1}H NMR (400 MHz, $CDCl_3$): 3.68 (*s*, 3H), 6.68 (*d*, 2H, *J* = 7.0 Hz), 6.91 (*d*, 2H, *J* = 7.0 Hz), 7.32-7.48 (*m*, 6H), 7.78 (*d*, 2H, *J* = 7.5 Hz), 7.87 (*d*, 2H, *J* = 7.5 Hz).

^{13}C NMR (101 MHz, $CDCl_3$): 55.3, 113.9, 122.2, 128.0, 128.7, 128.9, 129.3, 131.4, 134.3, 134.5, 135.4, 142.3, 157.0, 165.4, 198.7.

The data are consistent with the literature values.[25]

Spirolactamization of Ketimines

1-Benzyl-1'-(4-methoxyphenyl)spiro[indoline-3,2'-pyrrolidine]-2,5'-dione (11a)

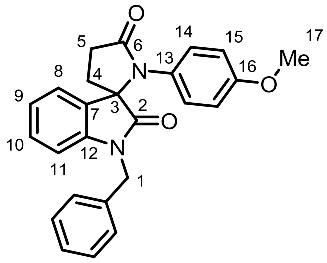

General Procedure 4 – 1-Benzyl-3-((4-methoxyphenyl)imino)indolin-2-one **10a** (57.1 mg, 0.167 mmol), P(*o*-$OMeC_6H_4)_3$ (9.20 mg, 15.0 mol%), Ni(cod)$_2$ (3.50 mg, 7.50 mol%), benzyl alcohol (1.75 μL, 10 mol%), and benzyl acrylate **2b** (78 μL, 0.508 mmol) were used. Purification by FCC (hexane:EtOAc, 1:1) afforded spirolactam **11a** (45.1 mg, 68%) as a colorless solid.

^{1}H NMR (400 MHz, $CDCl_3$): 2.40 (*ddd*, 1H, *J* = 13.0, 9.5, 7.5 Hz, **H4**), 2.66 (*ddd*, 1H, *J* = 13.0, 10.0, 5.5 Hz, **H4**), 2.86 (*ddd*, 1H, *J* = 17.0, 9.5, 5.5 Hz, **H5**), 3.06 (*ddd*¸ 1H, *J* = 17.0, 10.0, 7.5 Hz, **H5**), 3.72 (*s*, 3H, **H17**), 4.51 (*d*, 1H, *J* = 16.0 Hz, **H1**), 5.09 (*d*, 1H, *J* = 16.0 Hz,

H1), 6.53-6.58 (*m*, 1H, **H11**), 6.67-6.73 (*m*, 2H, **H15**), 6.75-6.80 (*m*, 2H, **ArH**), 6.86-6.93 (*m*, 2H, **H14**), 7.03-7.23 (*m*, 5H, **ArH**, **H10** and **H9**), 7.42-7.47 (*m*, 1H, **H8**).

^{13}C NMR (100 MHz, $CDCl_3$): 30.0 (**C5**), 30.5 (**C4**), 43.7 (**C1**), 55.4 (**C17**), 70.8 (**C3**), 110.0 (**C11**), 114.5 (**C15**), 123.5 (**C9**), 124.1 (**C8**), 126.8 (**ArC**), 127.7 (**ArC**), 128.7 (2×C, **C13** and **ArC**), 129.1 (**C7**), 129.5 (**C14**), 130.2 (**C10**), 134.7 (**ArC**), 142.4 (**C12**), 159.2 (**C16**), 176.0 (**C2**), 176.3 (**C6**).

ν_{max} / cm^{-1} (neat): 3059, 2938, 1705, 1612, 1510, 1467, 1358.

m/z (ESI$^+$): $C_{25}H_{23}N_2O_3$ [M+H]$^+$ requires 399.1703, found 399.1719.

M.P.: 172-175 °C (CH_2Cl_2).

1-Benzyl-7-fluoro-1'-(4-methoxyphenyl)spiro[indoline-3,2'-pyrrolidine]-2,5'-dione (11b)

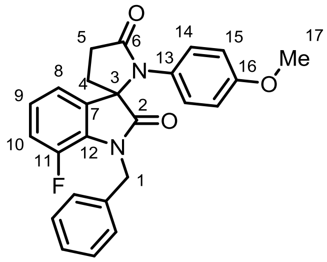

General Procedure 4 – 1-Benzyl-7-fluoro-3-((4-methoxyphenyl)imino)indolin-2-one **10b** (60.0 mg, 0.166 mmol), P(*o*-OMeC_6H_4)$_3$ (9.20 mg, 15.0 mol%), Ni(cod)$_2$ (3.50 mg, 7.50 mol%), benzyl alcohol (1.75 μL, 10 mol%), and benzyl acrylate **2b** (78 μL, 0.508 mmol) were used. Purification by FCC (hexane:EtOAc, 1:1) afforded spirolactam **11b** (48.8 mg, 71%) as a colorless solid.

^{1}H NMR (400 MHz, $CDCl_3$): 2.35 (*ddd*, 1H, *J* = 13.0, 9.5, 7.5 Hz, **H4**), 2.64 (*ddd*, 1H, *J* = 13.0, 10.0, 5.5 Hz, **H4**), 2.83 (*ddd*, 1H, *J* = 17.0, 9.5, 5.5 Hz, **H5**), 3.04 (*ddd*, 1H, *J* = 17.0, 10.0, 7.5 Hz, **H5**), 3.72 (*s*, 3H, **H17**), 4.77 (*d*, 1H, *J* = 15.5 Hz, **H1**), 5.12 (*d*, 1H, *J* = 15.5 Hz, **H1**), 6.66-6.70 (*m*, 2H, **H15**), 6.85-6.89 (*m*, 4H, **H14** and **ArH**), 6.95 (*ddd*, 1H, *J* = 11.0, 8.5, 1.0 Hz, **H10**), 7.03-7.08 (*m*, 1H, **H9**), 7.12-7.23 (*m*, 4H, **H8**, **ArH**).

^{13}C NMR (100 MHz, $CDCl_3$): 29.8 (**C5**), 31.0 (**C4**), 45.5 (*d*, 1C, *J* = 5.0 Hz, **C1**), 55.4 (**C17**), 70.8 (*d*, 1C, *J* = 2.5 Hz, **C3**), 114.6 (**C15**), 118.3 (*d*, 1C, *J* = 19.5 Hz, **C10**), 120.0 (*d*, 1C, *J* = 3.5 Hz, **C8**), 124.4 (*d*, 1C, *J* = 6.5 Hz, **C9**), 127.0 (*d*, 1C, *J* = 1.5 Hz, **ArC**), 127.6 (**ArC**), 128.5 (**C13**), 128.6 (**ArC**), 129.2 (*d*, 1C, *J* = 9.0 Hz, **C12**), 129.3 (**C14**), 132.3 (*d*, 1C, *J* =

3.0 Hz, **C7**), 136.0 (*d*, 1C, *J* = 0.5 Hz, **ArC**), 147.6 (*d*, 1C, *J* = 246.0 Hz, **C11**), 159.2 (**C16**), 175.9 (**C2**), 176.0 (**C6**).

^{19}F NMR (377 MHz, $CDCl_3$): -132.6 (*dd*, 1F, *J* = 11.0, 4.0 Hz).

ν_{max} / cm^{-1} (neat): 3064, 2933, 1717, 1703, 1630, 1510, 1346, 1246.

m/z (ESI$^+$): $C_{25}H_{22}N_2O_3F$ [M+H]$^+$ requires 417.1609, found 417.1606.

M.P.: 179-182 °C (CH_2Cl_2).

1-Benzyl-1'-(4-methoxyphenyl)-7-(trifluoromethyl)spiro[indoline-3,2'-pyrrolidine]-2,5'-dione (11c)

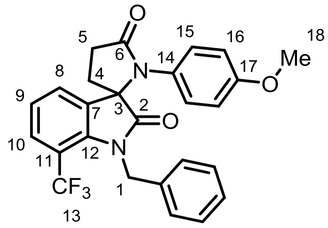

General Procedure 4 – 1-Benzyl-3-((4-methoxyphenyl)imino)indolin-2-one **10c** (68.6 mg, 0.167 mmol), P(*o*-OMeC_6H_4)$_3$ (9.20 mg, 15.0 mol%), Ni(cod)$_2$ (3.50 mg, 7.50 mol%), benzyl alcohol (1.75 μL, 10 mol%), and benzyl acrylate **2b** (78 μL, 0.508 mmol) were used. Purification by FCC (EtOAc:hexane, 2:1) afforded spirolactam **11c** (66.3 mg, 85%) as a colorless oil.

^{1}H NMR (400 MHz, $CDCl_3$): 2.36 (*ddd*, 1H, *J* = 13.0, 9.5, 8.0 Hz, **H5**), 2.64 (*ddd*, 1H, *J* = 13.0, 10.0, 5.0 Hz, **H5**), 2.85 (*ddd*, 1H, *J* = 17.0, 9.5, 5.0 Hz, **H4**), 3.04 (*ddd*, 1H, *J* = 17.0, 10.0, 8.0 Hz, **H4**), 3.75 (*s*, 3H, **H18**), 4.96 (*d*, 1H, *J* = 17.0 Hz, **H1**), 5.25 (*d*, 1H, *J* = 17.0 Hz, **H1**), 6.60 (*d*, 2H, **ArH**), 6.72-6.74 (*m*, 2H, **H16**), 6.87-6.89 (*m*, 2H, **H15**), 7.07-7.10 (*m*, 2H, **ArH**), 7.13-7.17 (*m*, 1H, **ArH**), 7.25 (*dd*, 1H, *J* = 8.0, 7.5 Hz, **H9**), 7.58 (*dd*, 1H, *J* = 8.0, 1.5 Hz, **H10**), 7.69 (*dd*, 1H, *J* = 7.5, 1.5 Hz, **H8**).

^{13}C NMR (100 MHz, $CDCl_3$): 29.6 (**C4**), 31.5 (**C5**), 45.5 (*q*, 1C, *J* = 5.0 Hz, **C1**), 55.4 (**C18**), 69.1 (**C3**), 113.7 (*q*, 1C, *J* = 33.0 Hz, **C11**), 114.8 (**C16**), 123.0 (*q*, 1C, *J* = 272.0 Hz, **C13**), 123.1 (**C9**), 125.4 (**ArC**), 126.9 (**ArC**), 127.9 (**C8**), 128.4 (3×C, **C10**, **C14** and **ArC**), 129.7 (**C15**), 132.2 (**C7**), 135.3 (**ArC**), 140.7 (**C12**), 159.4 (**C17**), 176.2 (**C6**), 177.3 (**C2**).

^{19}F NMR (377 MHz, $CDCl_3$): -55.0 (*s*, 1F).

ν_{max} / cm^{-1} (neat): 3035, 2940, 1729, 1705, 1597, 1510, 1452, 1332

m/z (ESI^+): $C_{26}H_{22}N_2O_3F_3$ $[M+H]^+$ requires 467.1577, found 467.1571.

1'-(4-Methoxyphenyl)-1-methylspiro[indoline-3,2'-pyrrolidine]-2,5'-dione (11d)

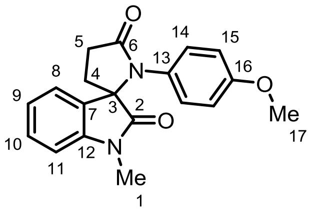

General Procedure 4 – A standard solution of 3-((4-methoxyphenyl)imino)-1-methylindolin-2-one **10d** (0.34 mL, 0.5 M in PhMe, 0.170 mmol), P(*o*-$OMeC_6H_4)_3$ (9.00 mg, 15.0 mol%), $Ni(cod)_2$ (3.50 mg, 7.50 mol%), benzyl alcohol (1.75 µL, 10 mol%), and benzyl acrylate **2b** (78 µL, 0.508 mmol) were used. Purification by FCC (EtOAc:hexane, 1:1) afforded spirolactam **11d** (33.4 mg, 61%) as a colorless solid.

^{1}H NMR (400 MHz, $CDCl_3$): 2.32 (*ddd*, 1H, *J* = 13.0, 9.5, 8.0 Hz, **H4**), 2.56 (*ddd*, 1H, *J* = 13.0, 9.5, 5.0 Hz, **H4**), 2.79 (*ddd*, 1H, *J* = 17.0, 9.5, 5.0 Hz, **H5**), 3.03 (*ddd*, 1H, *J* = 17.0, 9.5, 8.0 Hz, **H5**), 3.10 (*s*, 3H, **H1**), 3.67 (*s*, 3H, **H17**), 6.66-6.70 (*m*, 2H, **H15**), 6.73 (*d*, 1H, *J* = 8.0 Hz, **H11**), 6.87-6.91 (*m*, 2H, **H14**), 7.10 (*dd*, 1H, *J* = 7.5, 7.5 Hz, **H9**), 7.29 (*dd*, 1H, *J* = 8.0, 7.5 Hz, **H10**), 7.37 (*d*, 1H, *J* = 7.5 Hz, **H8**).

^{13}C NMR (100 MHz, $CDCl_3$): 26.5 (**C1**), 29.9 (**C5**), 30.9 (**C4**), 55.4 (**C17**), 70.7 (**C3**), 108.9 (**C11**), 114.4 (**C15**), 123.5 (**C9**), 123.9 (**C8**), 128.8 (2×C, **C7** and **C13**), 128.9 (**C14**), 130.1 (**C10**), 143.3 (**C12**), 158.9 (**C16**), 176.2 (2×C, **C2** and **C6**).

ν_{max} / cm^{-1} (neat): 3064, 2924, 1710, 1698, 1613, 1508, 1472, 1364.

m/z (ESI^+): $C_{19}H_{19}N_2O_5$ $[M+H]^+$ requires 323.1390, found 323.1406.

M.P.: 176-177 °C (CH_2Cl_2).

1-Benzyl-5-methoxy-1'-(4-methoxyphenyl)spiro[indoline-3,2'-pyrrolidine]-2,5'dione (11e)

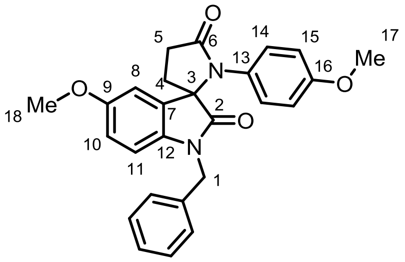

General Procedure 4 – 1-Benzyl-5-methoxy-3-((4-methoxyphenyl)imino)indolin-2-one **10e** (62.5 mg, 0.168 mmol), P(*o*-OMeC_6H_4)$_3$ (9.20 mg, 15.0 mol%), Ni(cod)$_2$ (3.50 mg, 7.50 mol%), benzyl alcohol (1.75 µL, 10 mol%), and benzyl acrylate **2b** (78 µL, 0.508 mmol) were used. Purification by FCC (EtOAc:hexane, 2:1) afforded spirolactam **11e** (36.1 mg, 67%) as a colorless solid.

^{1}H NMR (400 MHz, $CDCl_3$): 2.37 (*ddd*, 1H, *J* = 13.0, 9.5, 7.5 Hz, **H4**), 2.66 (*ddd*, 1H, *J* = 13.0, 10.0, 5.5 Hz, **H4**), 2.85 (*ddd*, 1H, *J* = 16.5, 9.5, 5.5 Hz, **H5**), 3.05 (*ddd*, 1H, *J* = 17.0, 10.0, 7.5 Hz, **H5**), 3.73 (*s*, 3H, **H17**), 3.77 (*s*, 3H, **H18**), 4.48 (*d*, 1H, *J* = 16.0 Hz, **H1**), 5.07 (*d*, 1H, *J* = 16.0 Hz, **H1**), 6.45 (*d*, 1H, *J* = 8.5 Hz, **H11**), 6.67-6.76 (*m*, 5H, **ArH**, **H15** and **H10**), 6.92 (*d*, 2H, *J* = 8.5 Hz, **H14**), 7.03 (*d*, 1H, *J* = 2.5 Hz, **H8**), 7.12-7.20 (*m*, 3H, **ArH**).

^{13}C NMR (100 MHz, $CDCl_3$): 30.0 (**C5**), 30.6 (**C4**), 43.8 (**C1**), 55.4 (**C17**), 55.9 (**C18**), 71.1 (**C3**), 110.5 (**C11**), 111.2 (**C8**), 114.4 (**C10**), 114.6 (**C15**), 126.8 (**ArC**), 127.6 (**ArC**), 128.7 (**ArC**), 128.8 (**C13**), 129.4 (**C14**), 130.5 (**C7**), 134.8 (**ArC**), 135.7 (**C12**), 156.6 (**C9**), 159.2 (**C16**), 175.7 (**C2**), 176.2 (**C6**).

ν_{max} / cm^{-1} (neat): 3040, 2967, 1719, 1694, 1604, 1509, 1497, 1354.

m/z (ESI$^+$): $C_{26}H_{25}N_2O_4$ [M+H]$^+$ requires 429.1809, found 429.1998.

M.P.: 162-163 °C (CH_2Cl_2/Et_2O).

The structure of this compound was determined unambiguously by X-ray crystallography.

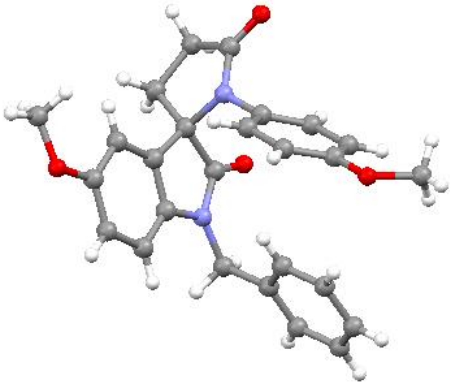

5-Benzoyl-1-(4-methoxyphenyl)-5-phenylpyrrolidin-2-one (12)

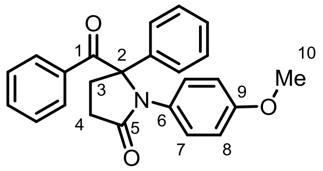

General Procedure 4 – 2-((4-Methoxylphenyl)imino)-1,2-diphenylethan-1-one (54.5 mg, 0.173 mmol), P(*o*-OMeC_6H_4)$_3$ (9.20 mg, 15.0 mol%), Ni(cod)$_2$ (3.50 mg, 7.50 mol%), benzyl alcohol (1.75 μL, 10 mol%), and benzyl acrylate **2b** (78 μL, 0.508 mmol) were used. Purification by FCC (hexane:EtOAc, 10:1) afforded lactam **12** (43.5 mg, 68%) as a colorless oil.

^{1}H NMR (400 MHz, $CDCl_3$): 2.48 (*ddd*, 1H, *J* = 12.5, 9.0, 7.5 Hz, **H3**), 2.58 (*ddd*, 1H, *J* = 16.5, 9.0, 6.0 Hz, **H4**), 2.76 (*ddd*, 1H, *J* = 16.5, 9.0, 7.5 Hz, **H4**), 3.69 (*s*, 3H, **H10**), 3.76 (*ddd*, 1H, *J* = 12.5, 9.0, 6.0 Hz, **H3**), 6.59-6.69 (*m*, 6H, **H7**, **H8**, and **ArH**), 7.03-7.09 (*m*, 2H, **ArH**), 7.10-7.14 (*m*, 1H, **ArH**), 7.30-7.39 (*m*, 3H, **ArH**), 7.45-7.47 (*m*, 2H, **ArH**).

^{13}C NMR (100 MHz, $CDCl_3$): 29.0 (**C4**), 34.3 (**C3**), 55.4 (**C10**), 91.8 (**C2**), 113.9 (**C8**), 122.6 (**ArC**), 125.0 (**ArC**), 127.8 (**ArC**), 128.4 (**ArC**), 128.8 (**ArC**), 128.7 (2×C, **C7** and **ArC**), 134.6 (**ArC**), 140.6 (**ArC**), 142.1 (**C6**), 156.6 (**C9**), 169.0 (**C1**), 176.3 (**C5**).

ν_{max} / cm^{-1} (neat): 3058, 2932, 1779, 1502, 1447, 1240.

m/z (ESI$^+$): $C_{24}H_{22}NO_3$ [M+H]$^+$ requires 372.1594, found 372.1614.

One-Pot Imine Formation-Lactamization

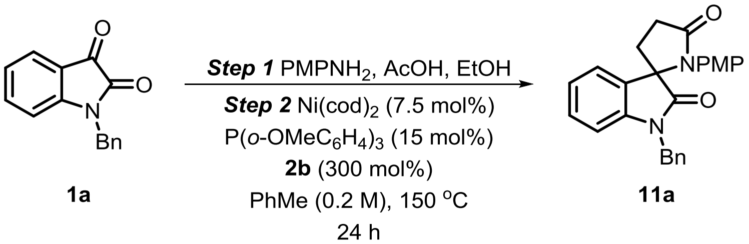

Isatin **1a** (40.2 mg, 0.169 mmol) and *p*-anisidine (25.4 mg, 0.206 mmol) were dissolved in an ethanoloic solution of AcOH (0.08 M, 0.85 mL) in a re-sealable tube fitted with a Young's tap. The vessel was sealed and heated at reflux for 2 hours. After this time, the mixture was cooled to room temperature and the volatiles were removed. The vessel was then charged with P(*o*-OMeC_6H_4)$_3$ (9.1 mg, 15 mol%), Ni(cod)$_2$ (3.50 mg, 7.5 mol), benzyl alcohol (1.75 µL, 10 mol%), benzyl acrylate **2b** (78 µL, 0.508 mmol) and PhMe (0.85 mL). The mixture was heated at 150 °C for 24 hours. The reaction was cooled to room temperature and concentrated *in vacuo*. The residue was purified by FCC (EtOAc:hex, 1:1) to afford spirolactone **3a** (33.5 mg, 50%) as a colorless solid.

NMR Spectra for Novel Compounds

Benzyl 1-benzyl-2,3-dioxoindoline-7-carboxylate (1h)

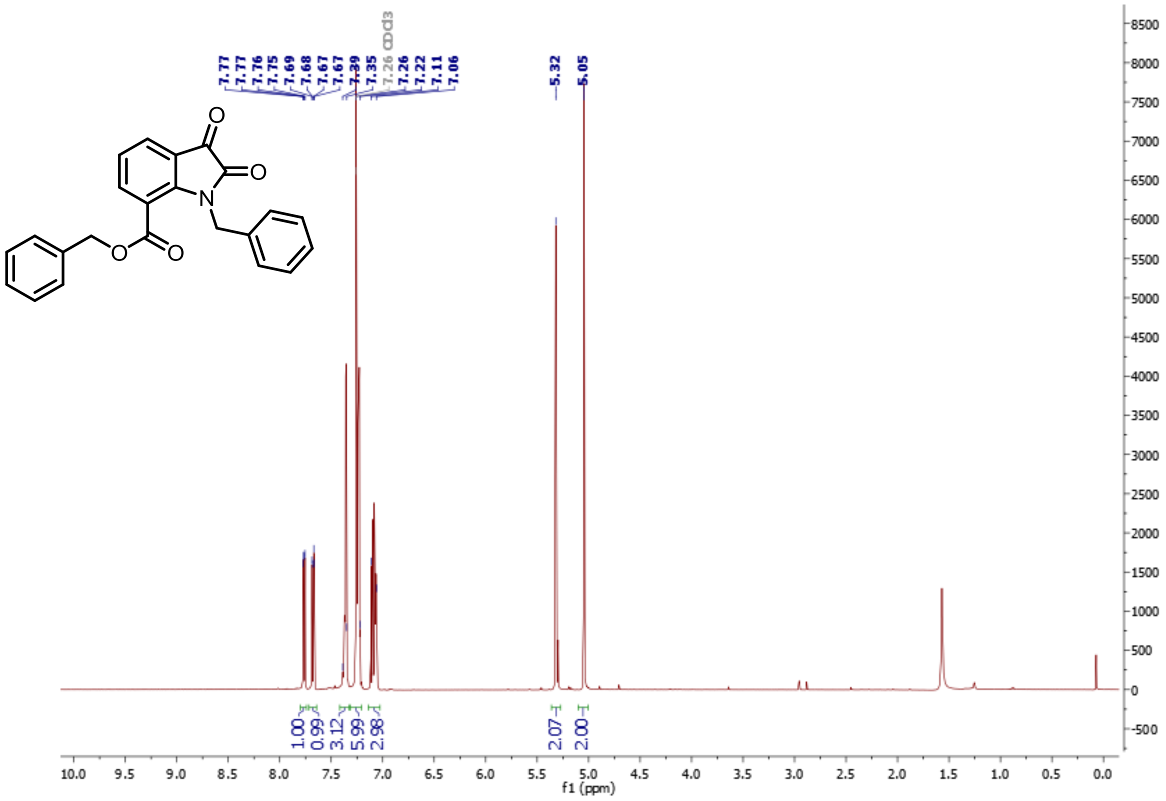

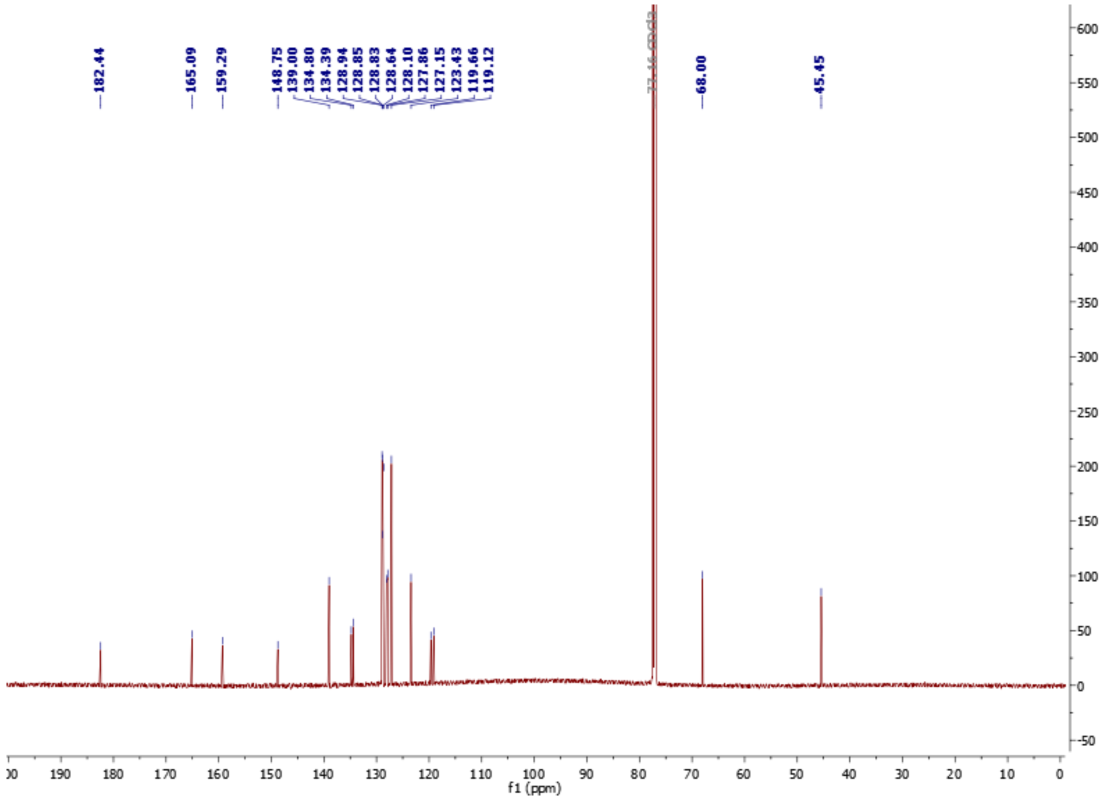

1-Benzyl-4,7-dimethylindoline-2,3-dione (1j)

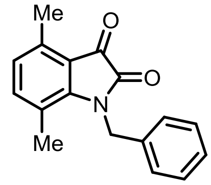

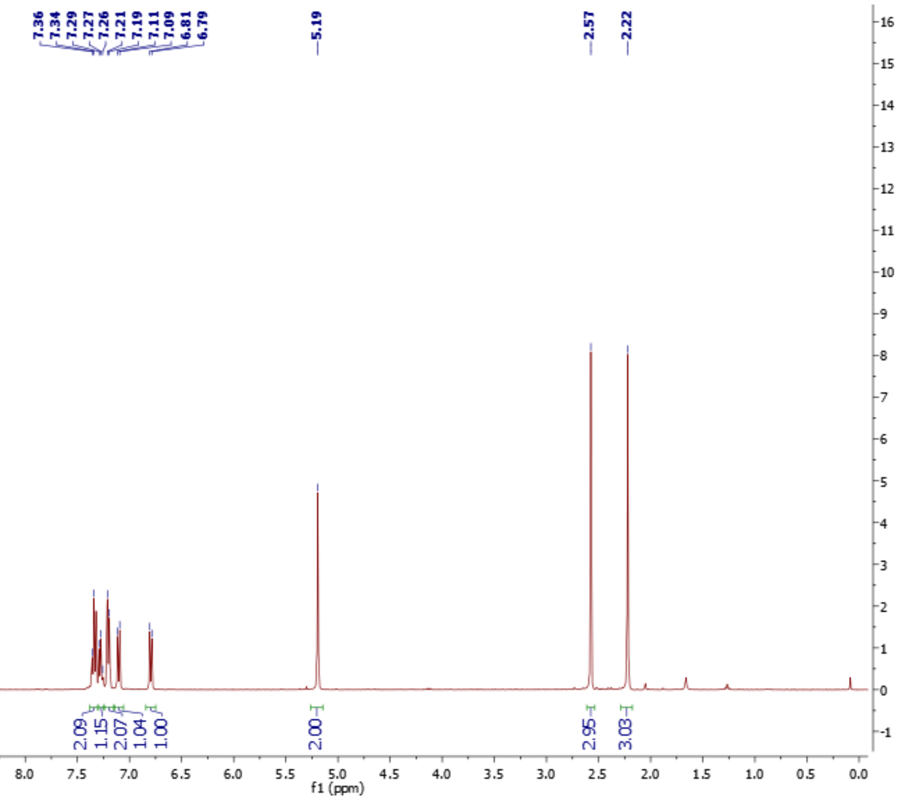

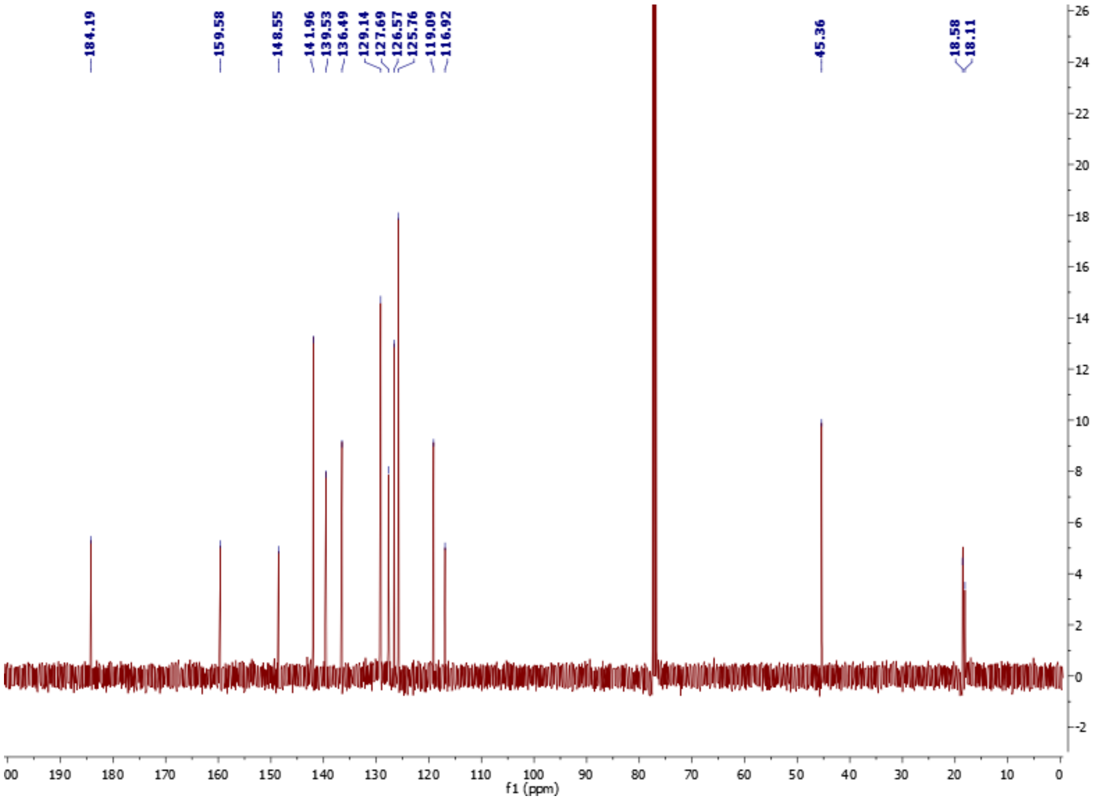

1’-Methyl-3,4-dihydro-*5H*-spiro[furan-2,3-indoline]-2’,5-dione (3b)

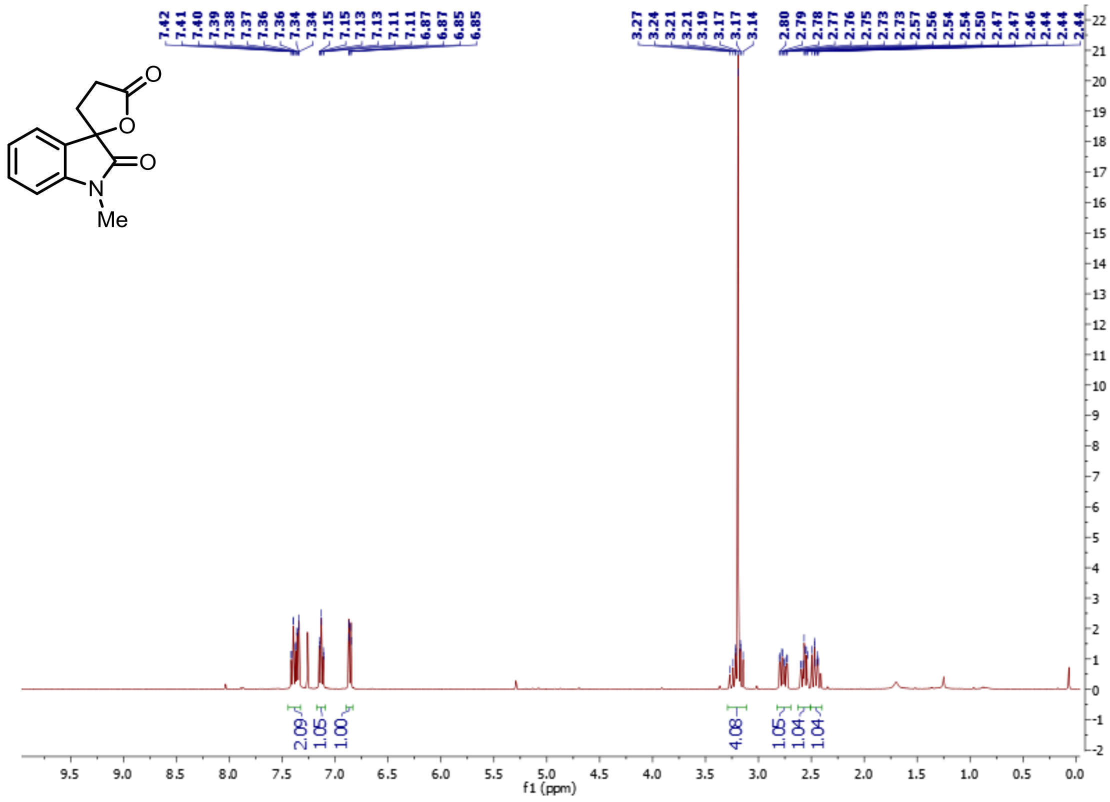

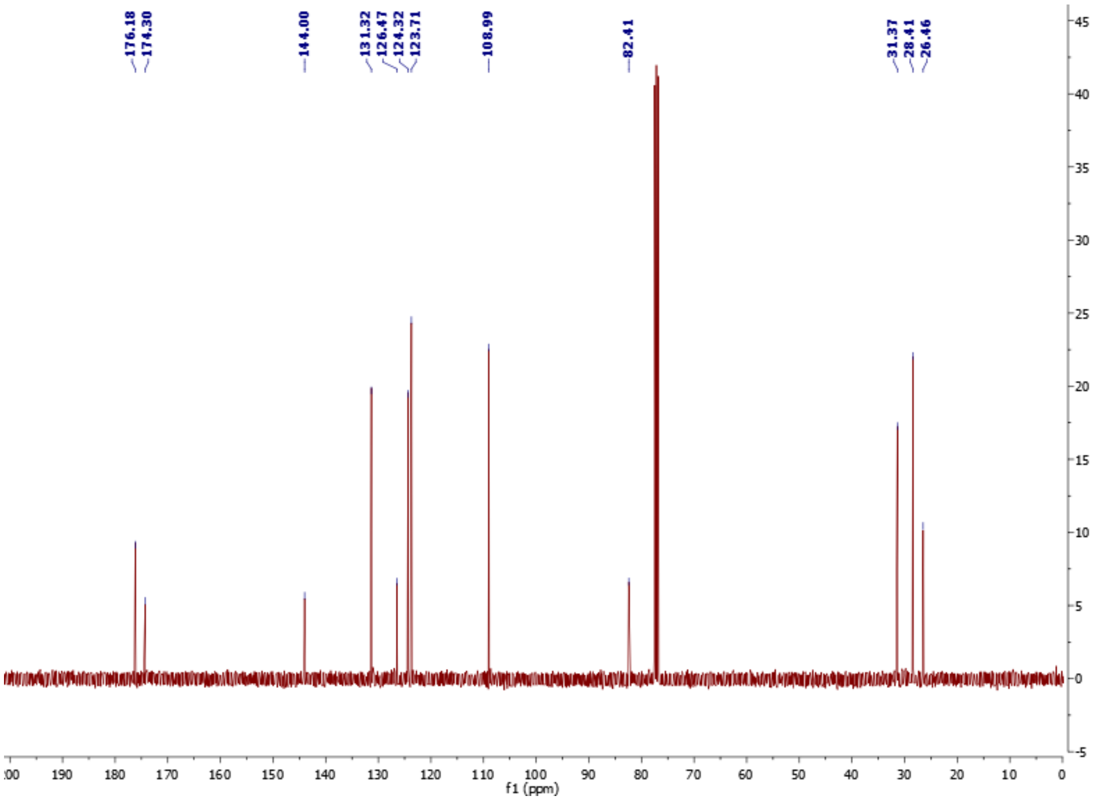

1’-Phenyl-3,4-dihydro-*5H*-spiro[furan-2,3-indoline]-2’,5-dione (3c)

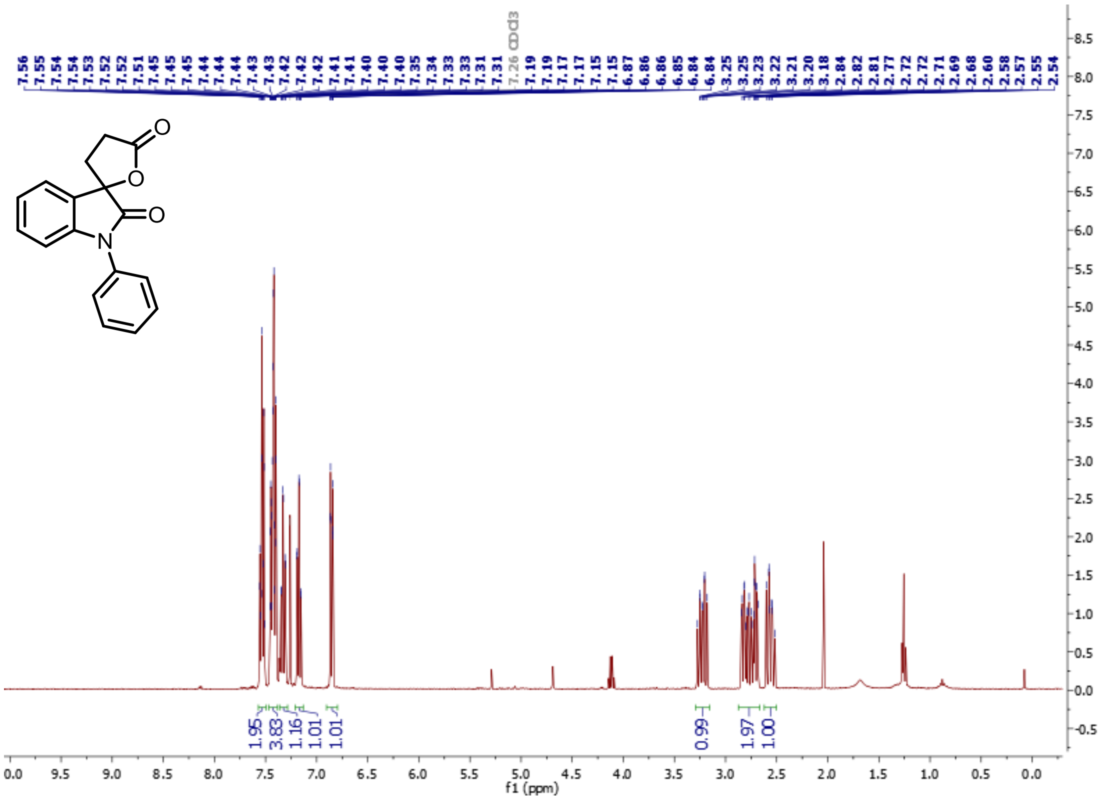

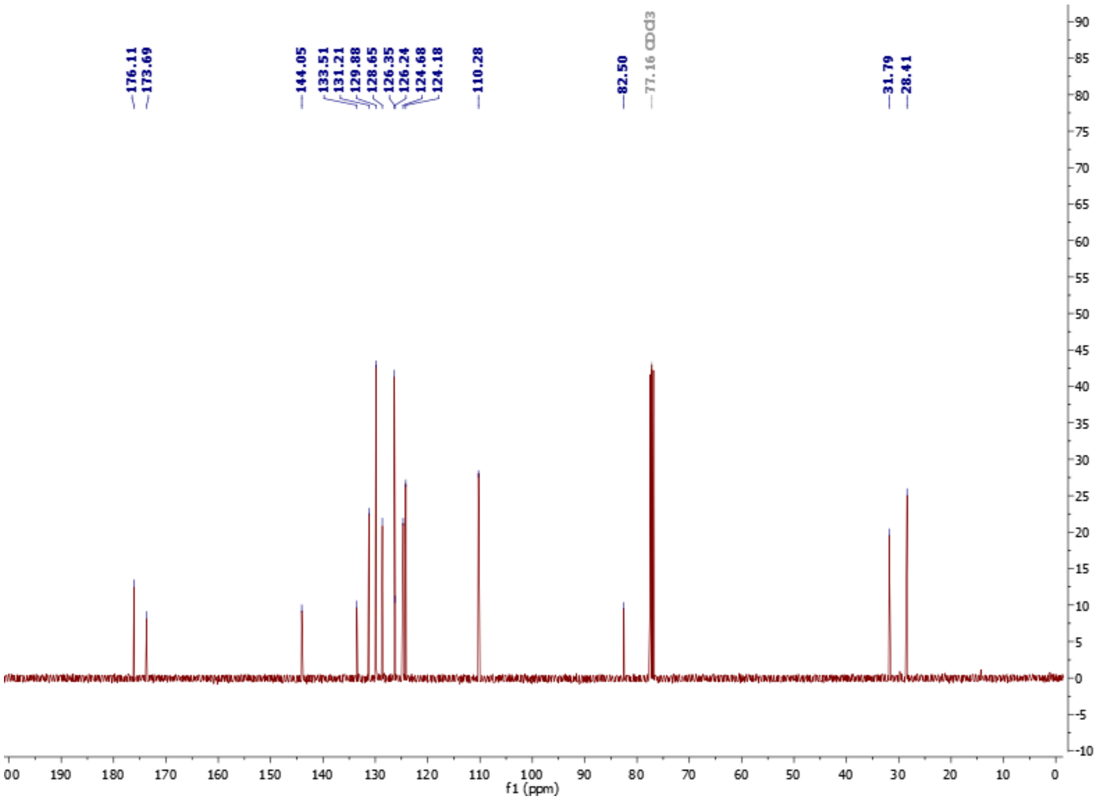

1’-Benzyl-5’-methoxy-3,4-dihydro-5*H*-spiro[furan-2,3’-indoline]-2’,5-dione (3d)

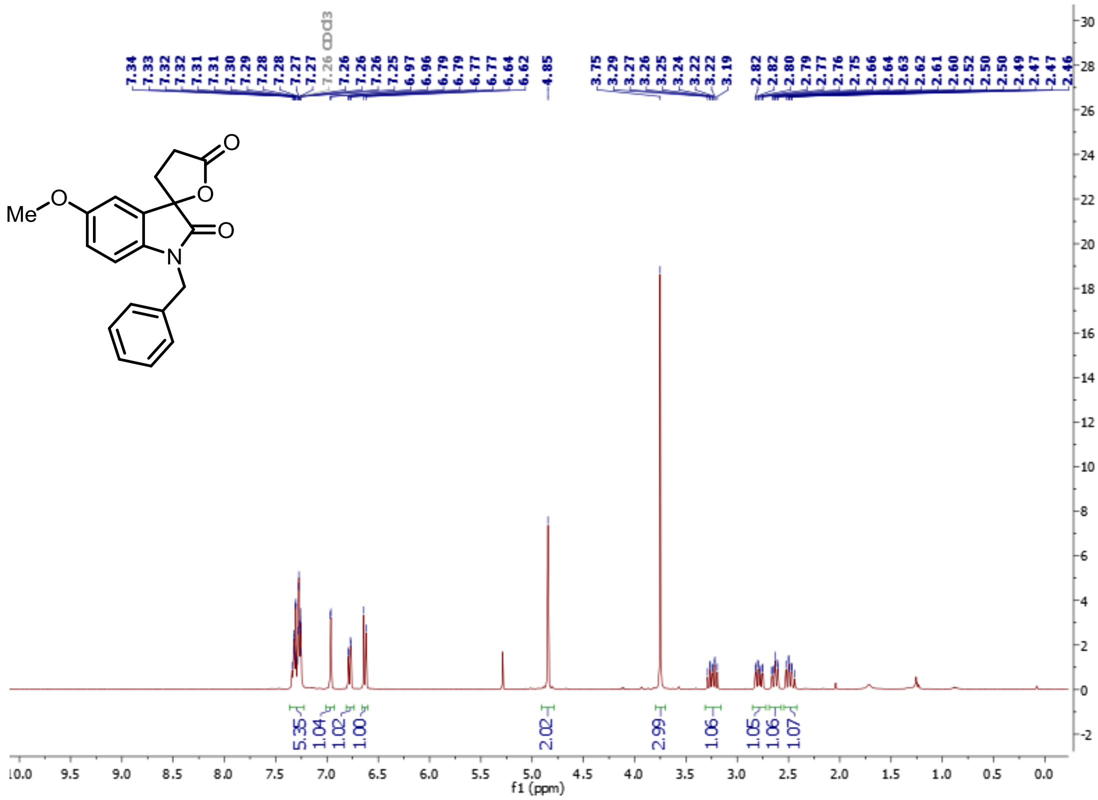

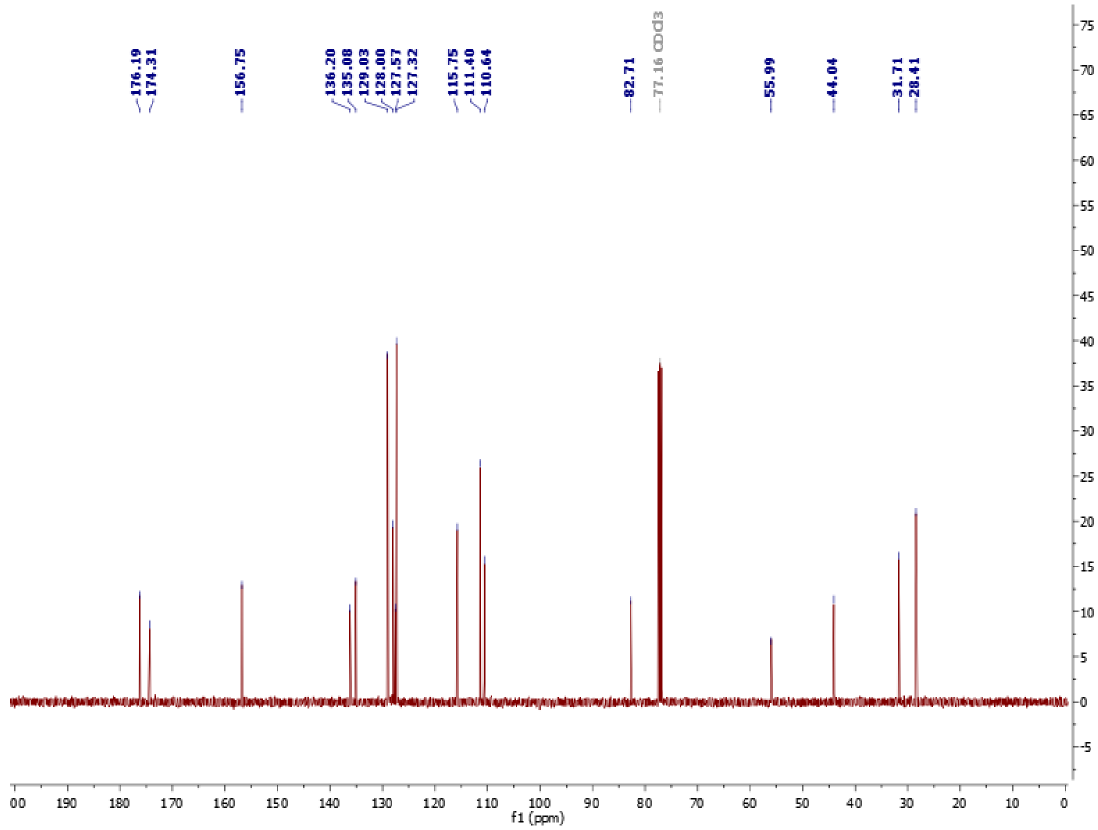

1’-Benzyl-5’-(trifluoromethoxy)-3,4-dihydro-5*H*-spiro[furan-2,3’-indoline]-2’,5-dione (3e)

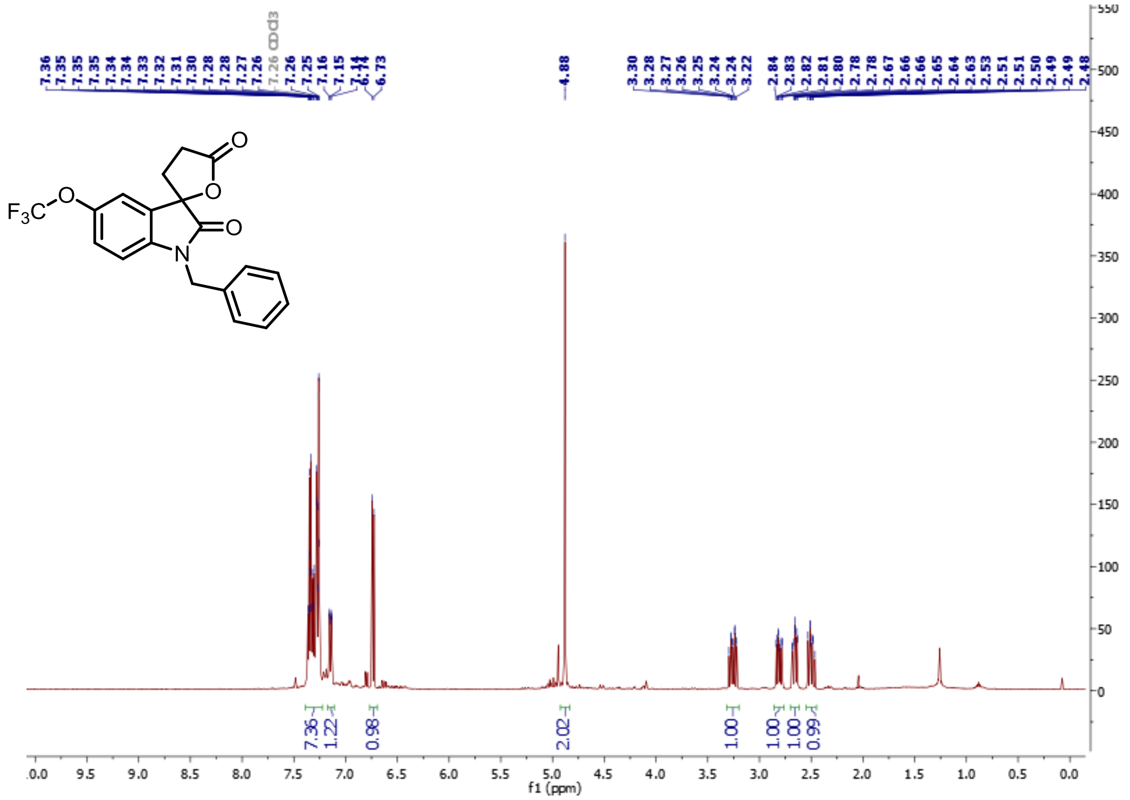

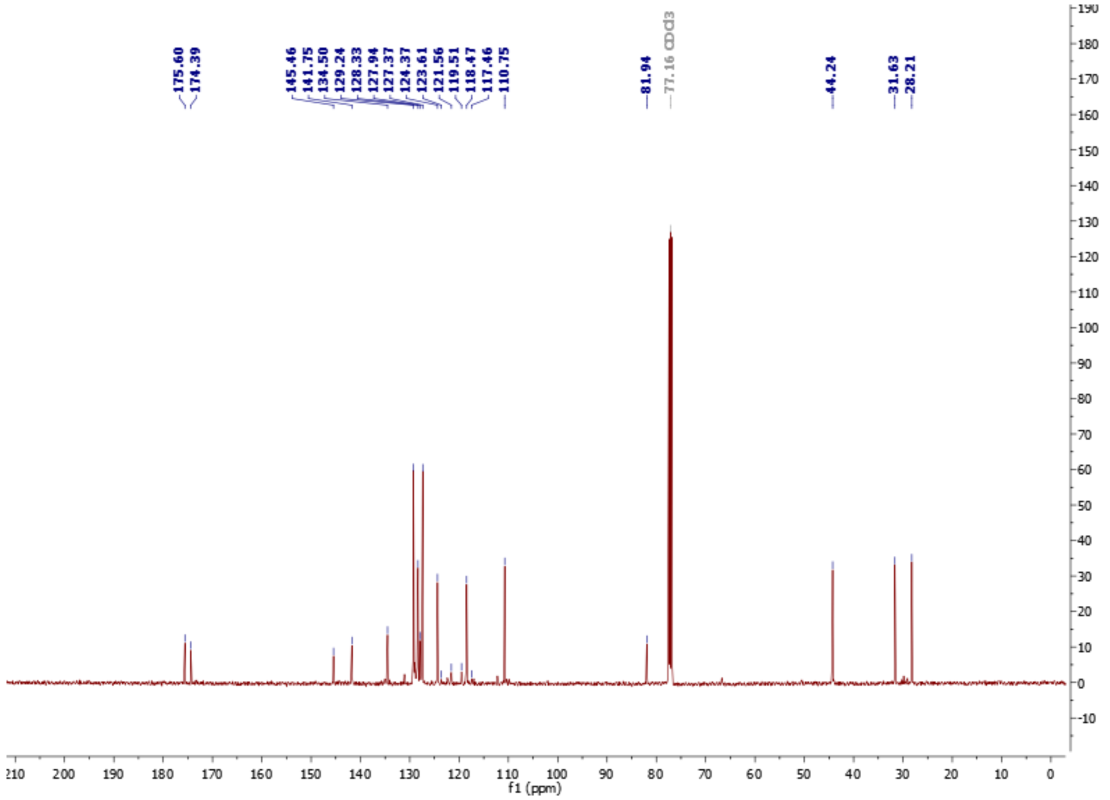

1’-Benzyl-7’-fluoro-3,4-dihydro-5*H*-spiro[furan-2,3’-indoline]-2’,5-dione (3f)

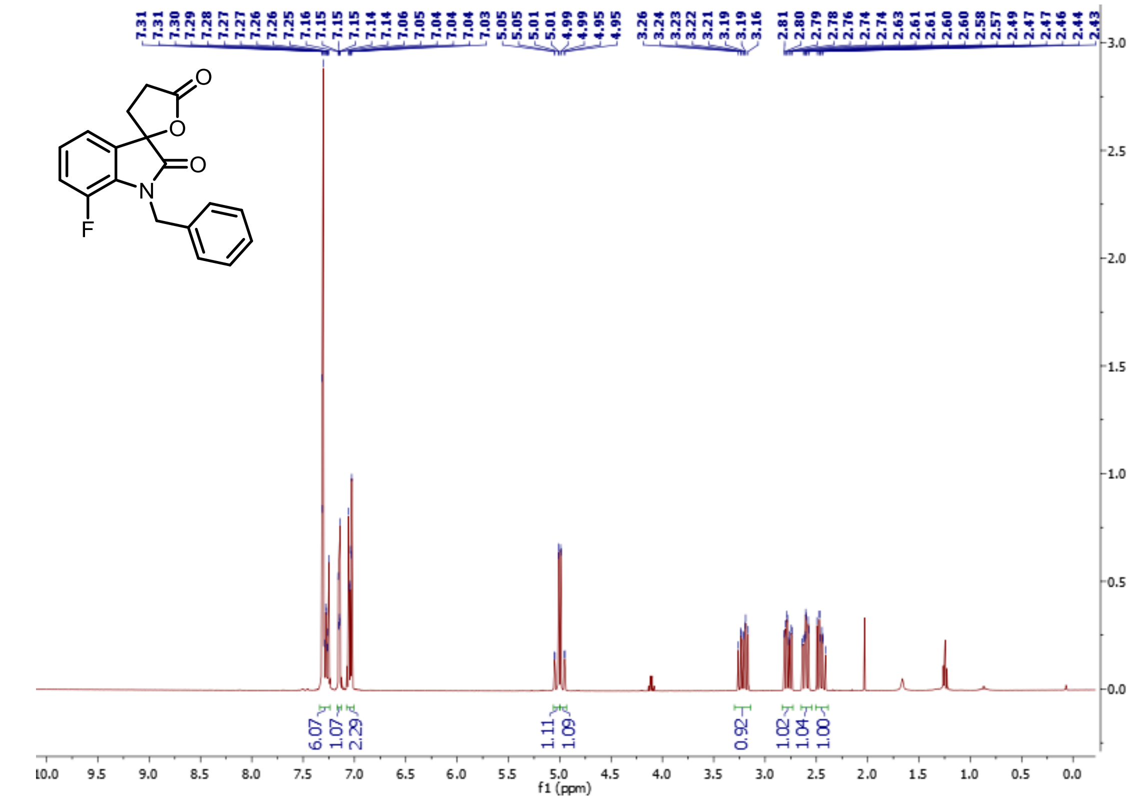

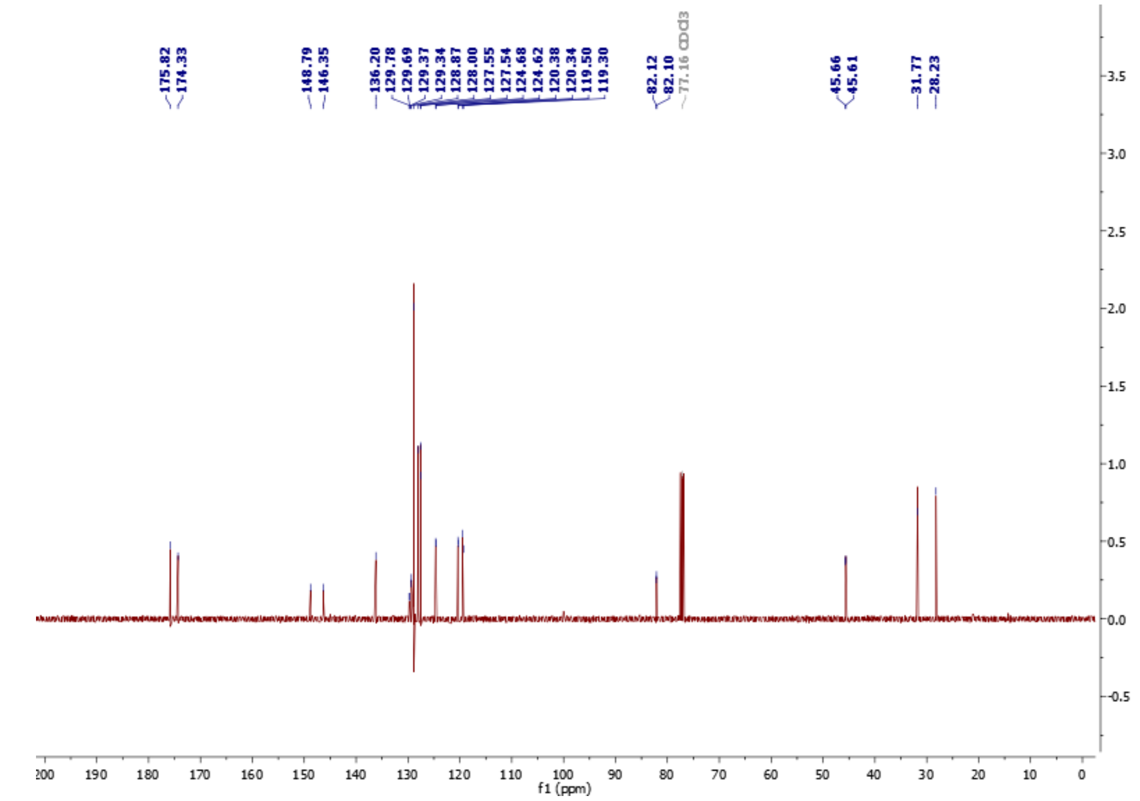

1'-Benzyl-7'-(trifluoromethyl)-3,4-dihydro-5*H*-spiro[furan-2,3'-indoline]-2',5-dione (3g)

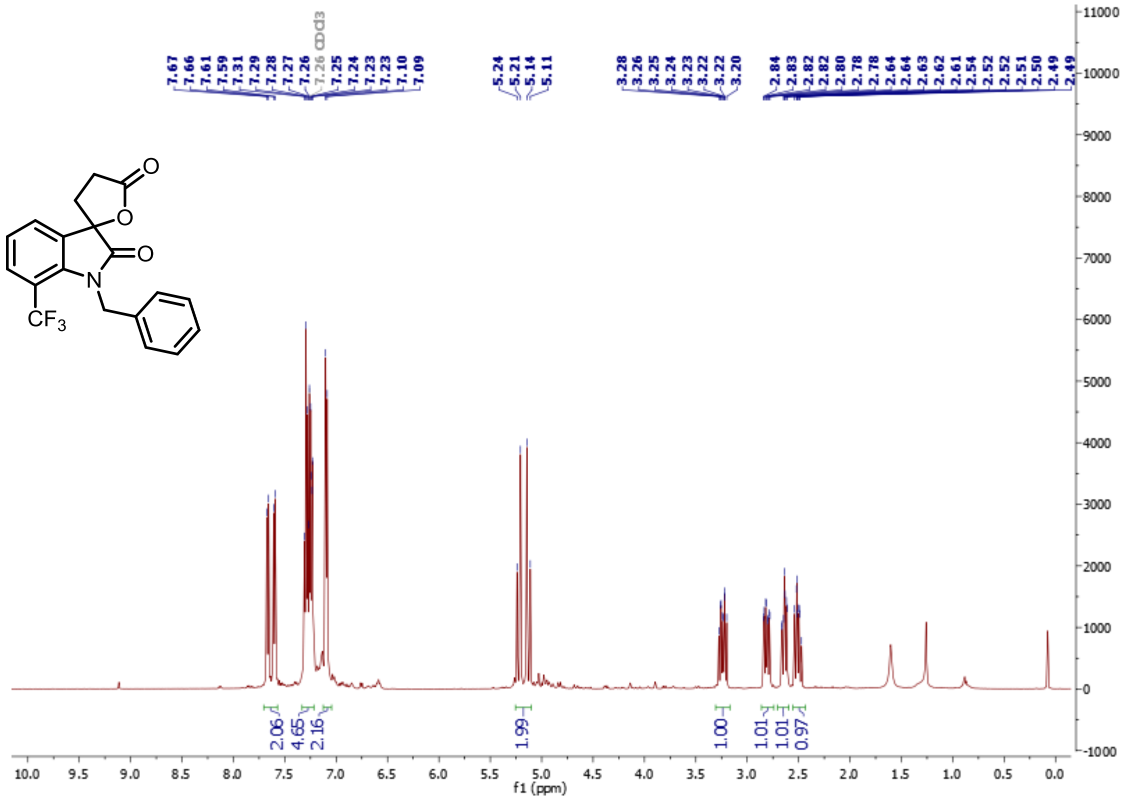

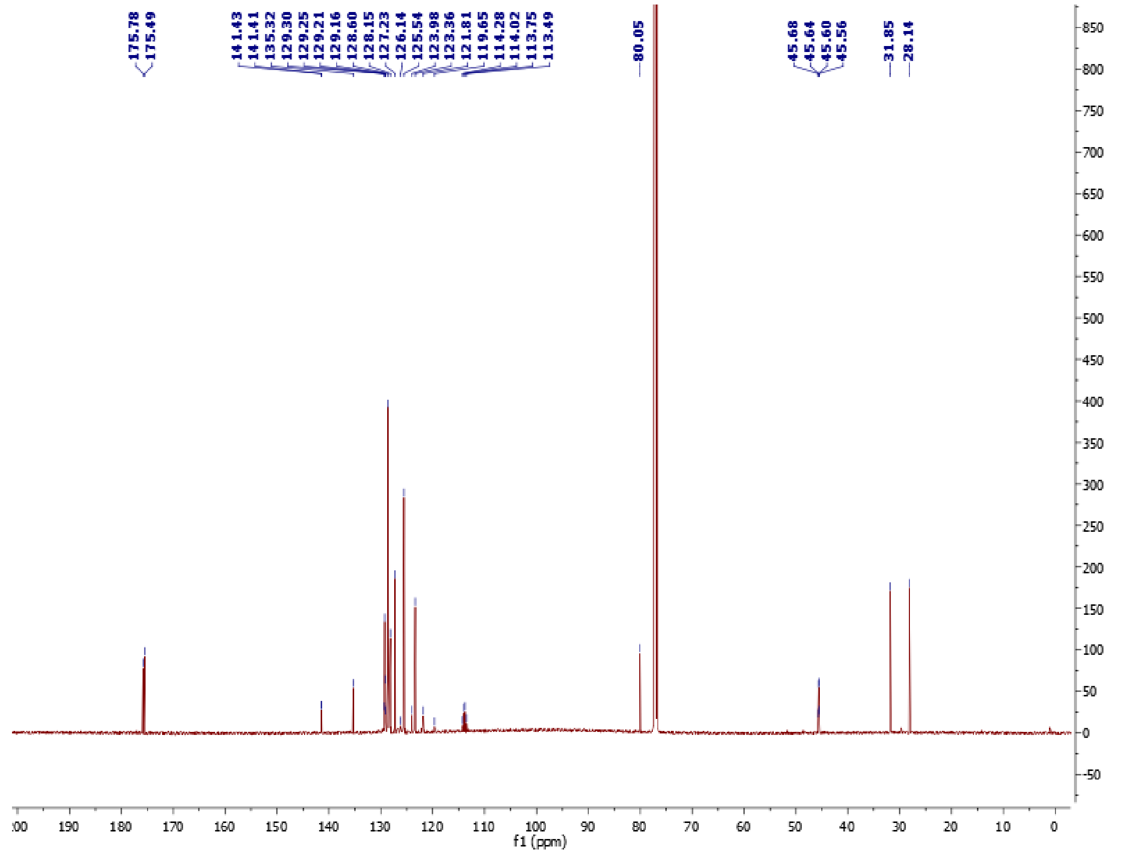

Benzyl 1'-benzyl-2',5-dioxo-4,5-dihydro-3*H*-spiro[furan-2,3'-indoline]-7'carboxylate (3h)

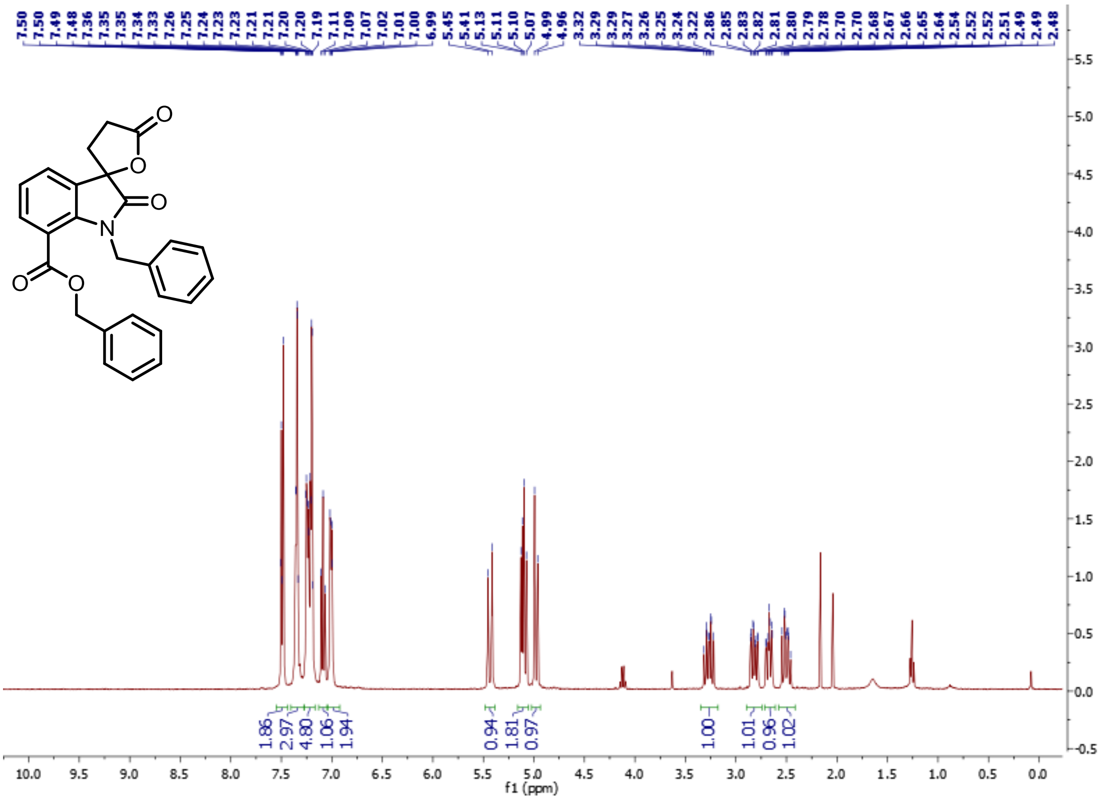

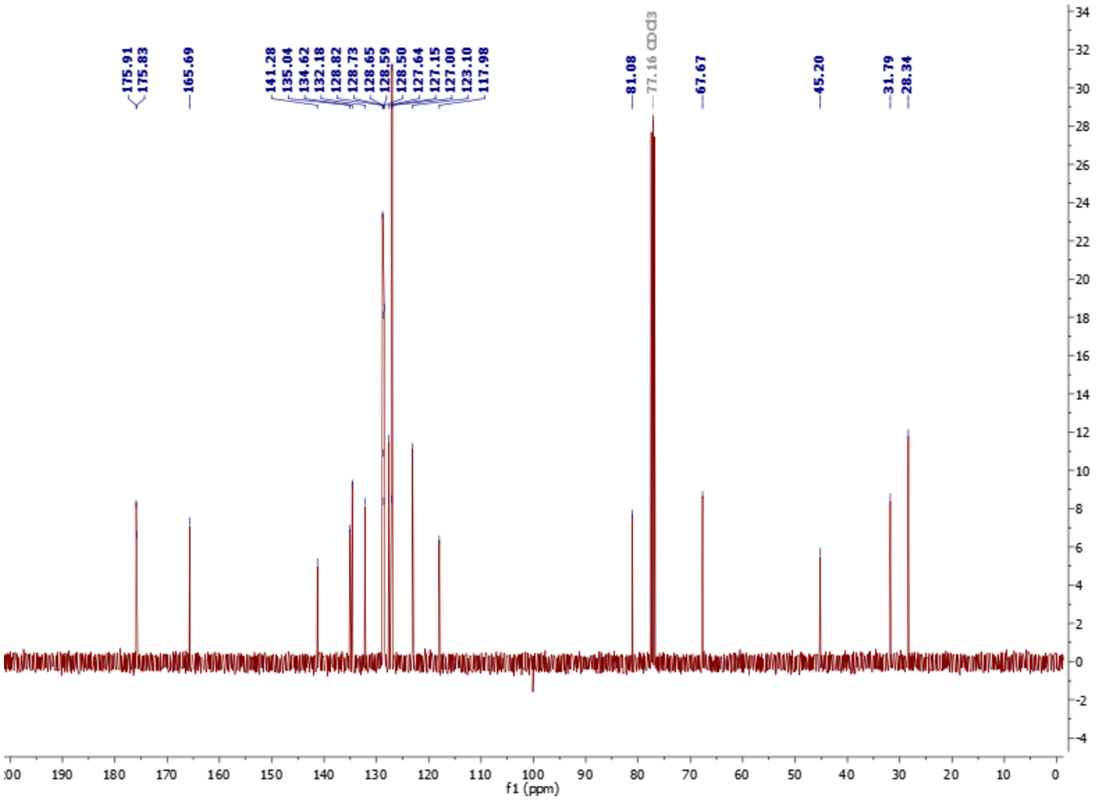

1’-Benzyl-7’-methyl-3,4-dihydro-5*H*-spiro[furan-2,3’-indoline]2’,5-dione (3i)

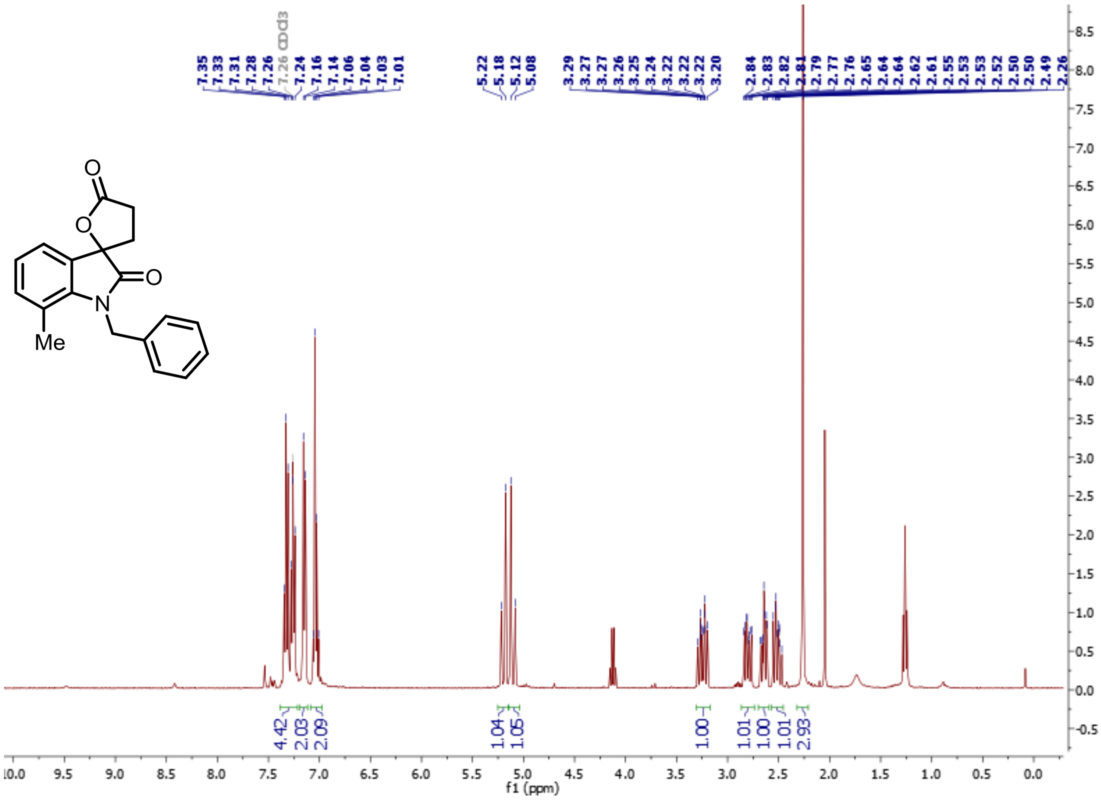

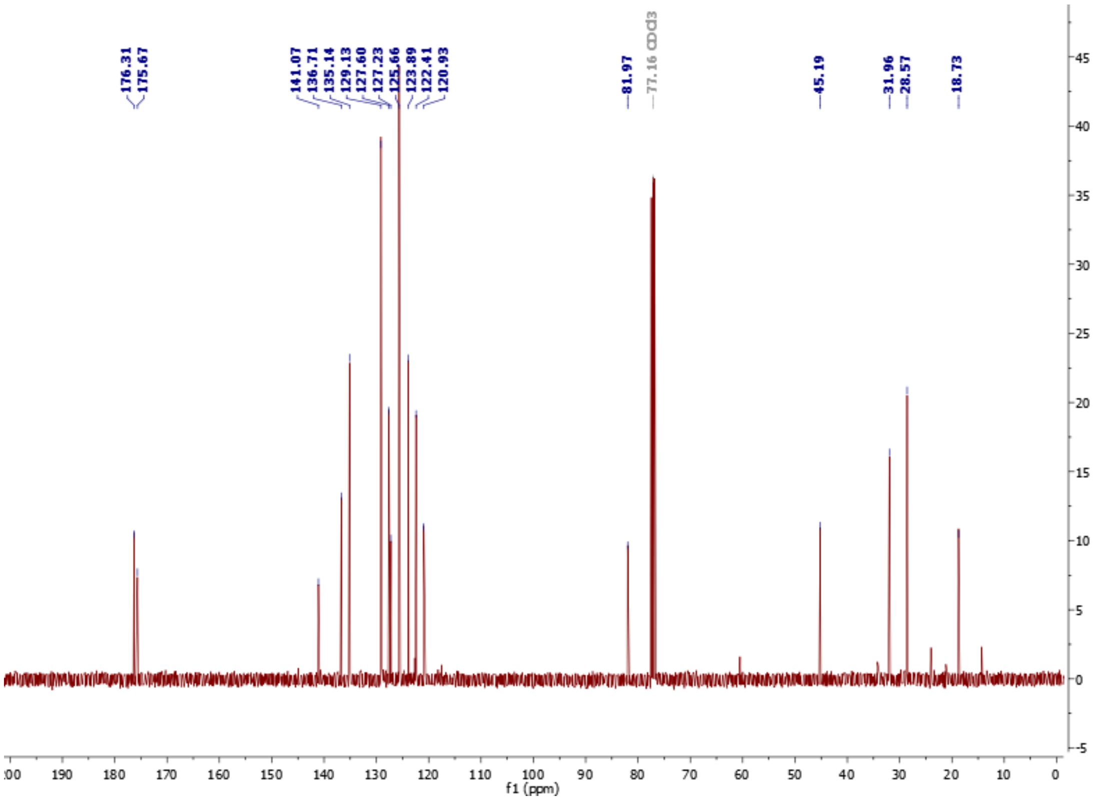

1’-Benzyl-4’,7’-dimethyl-3,4-dihydro-5*H*-spiro[furan-2,3’-indoline]-2’,5-dione (3j)

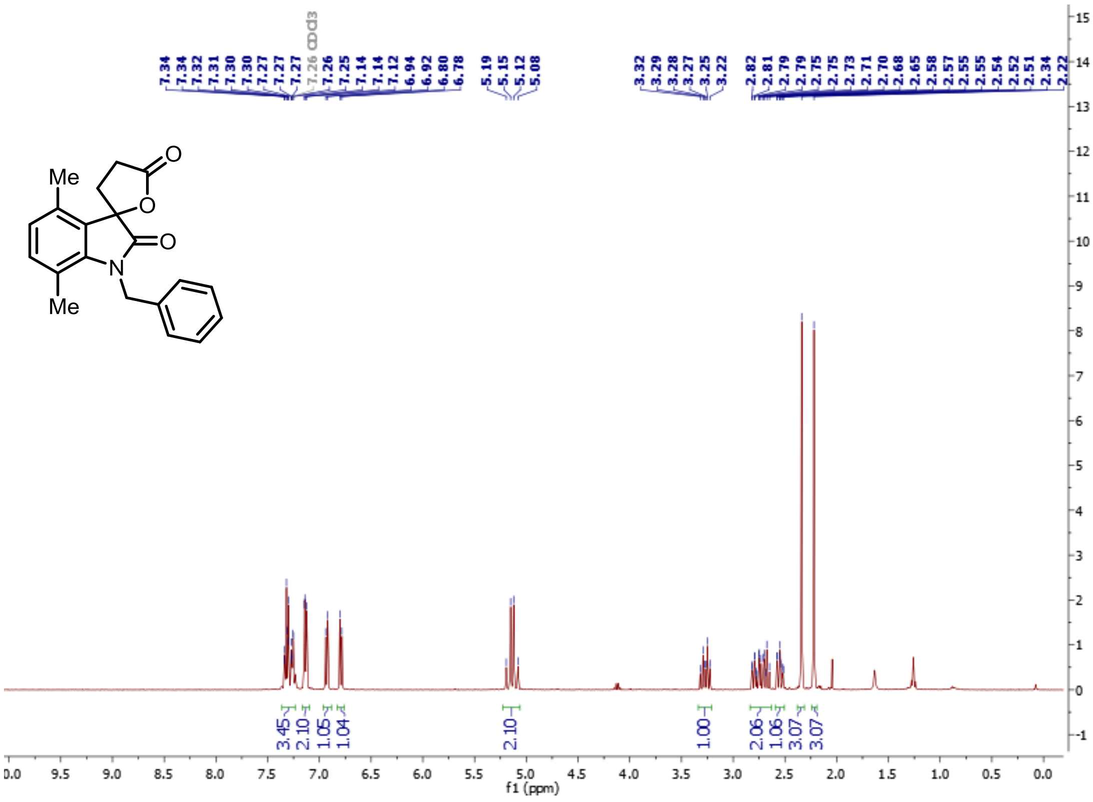

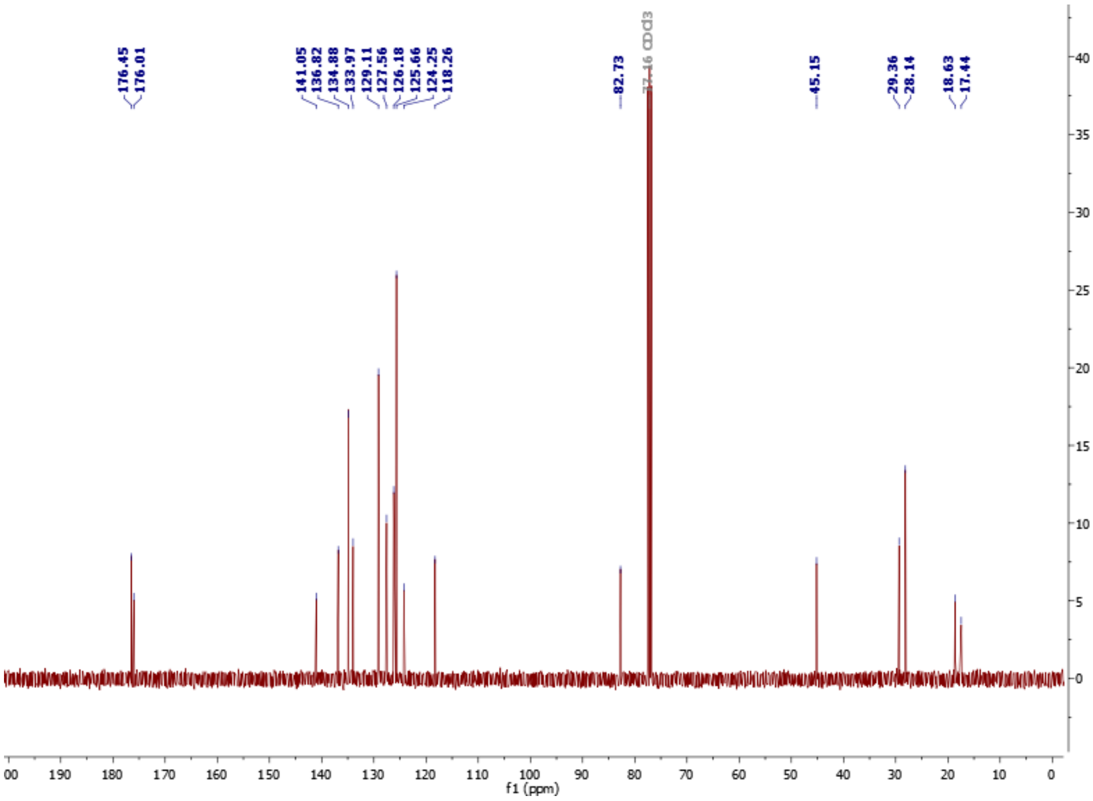

1’-Benzyl-4-phenyl-3,4-dihydro-5*H*-spiro[furan-2,3’-indoline]-2’,5-dione (3l)

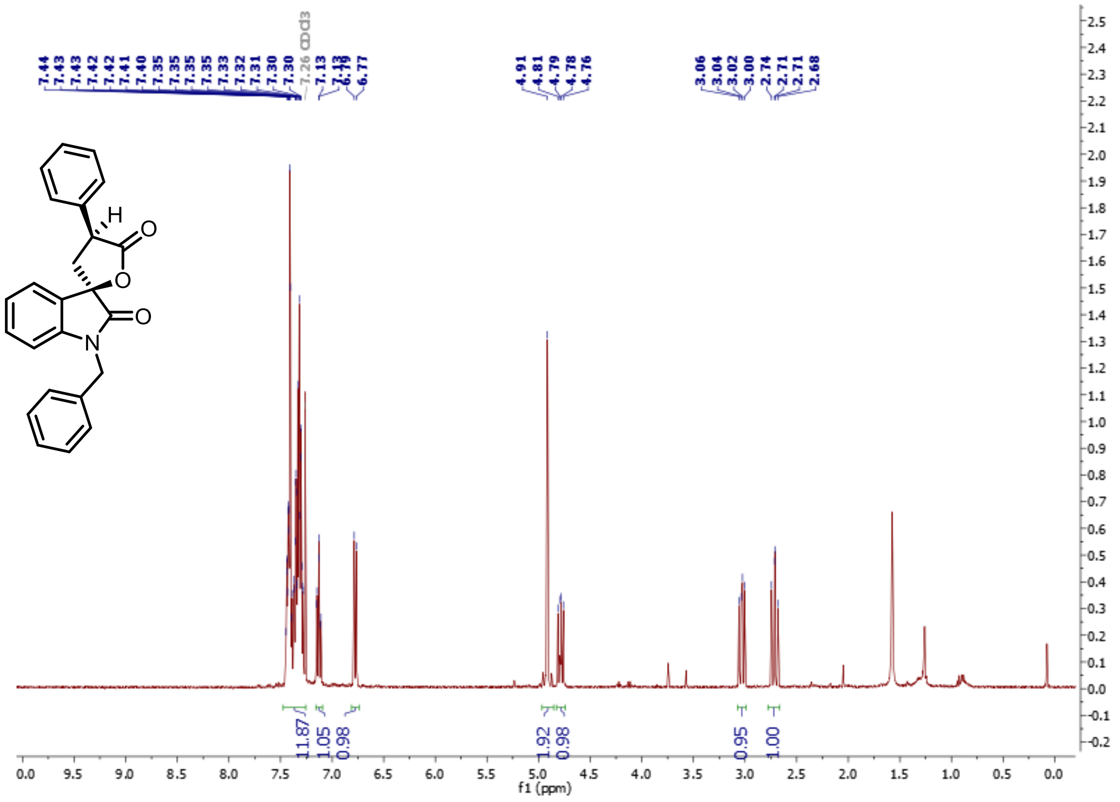

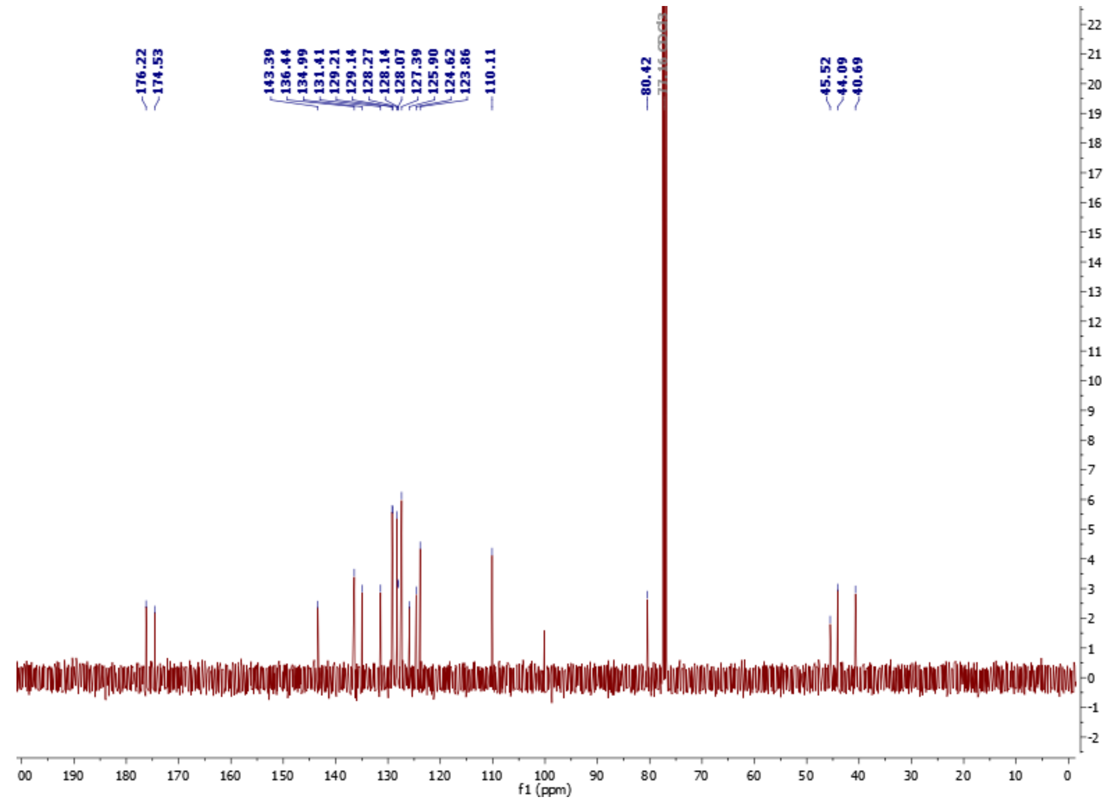

Benzyl 1'-Benzyl-2',5-dioxo-4,5-dihydro-3*H*-spiro[furan-2,3'-indoline]-3-carboxylate (3n)

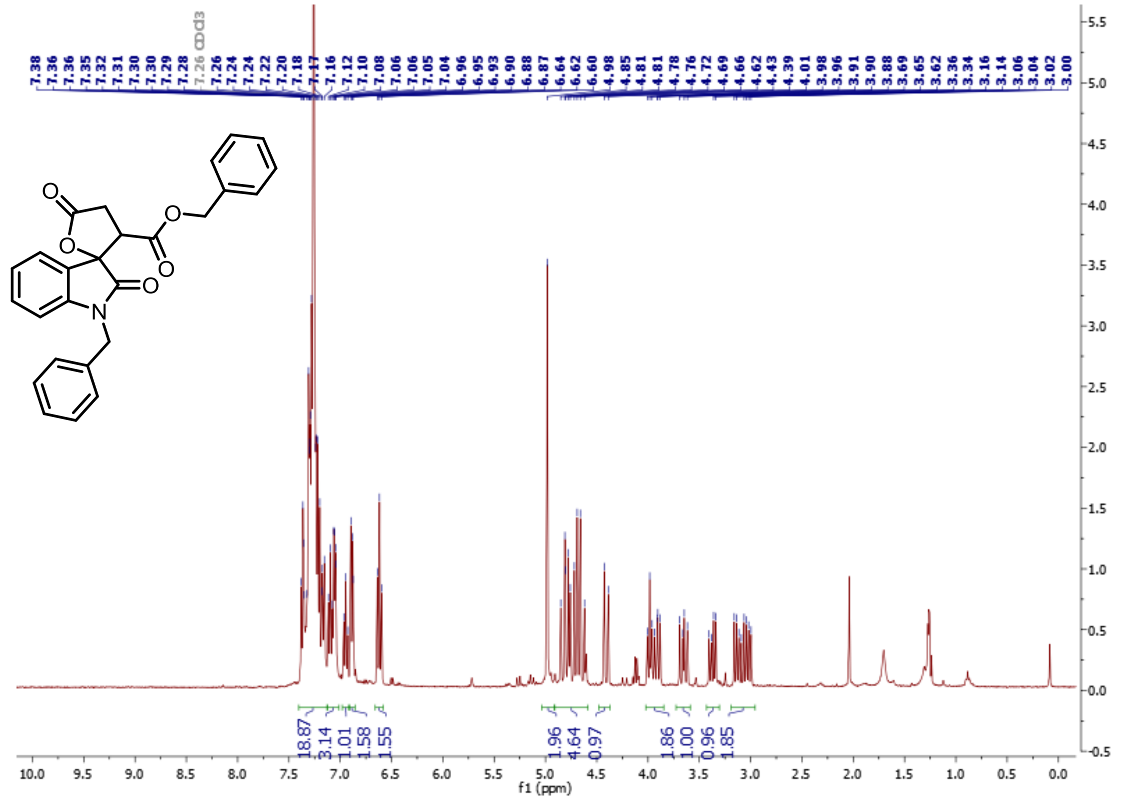

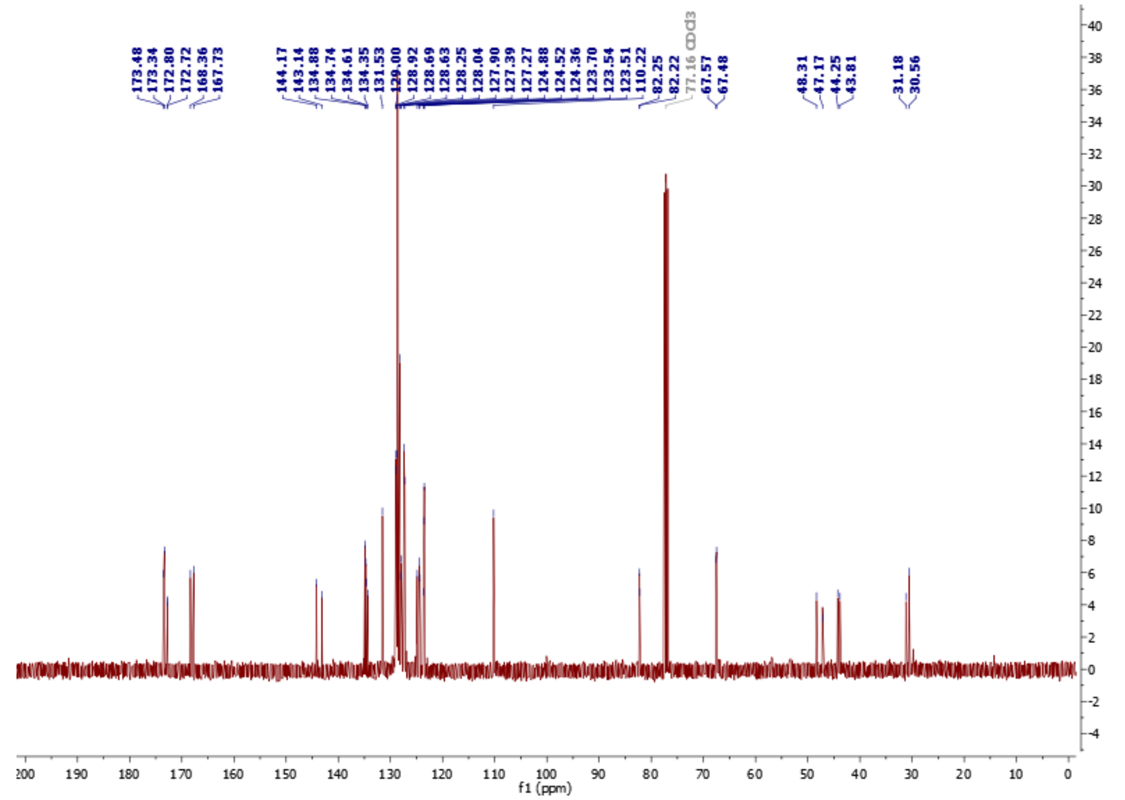

1’-Benzyl-3,4-dihydro-5*H*-spiro[furan-2,3’-indoline]2’,5-dione-4-*d* (*deuterio*-3a)

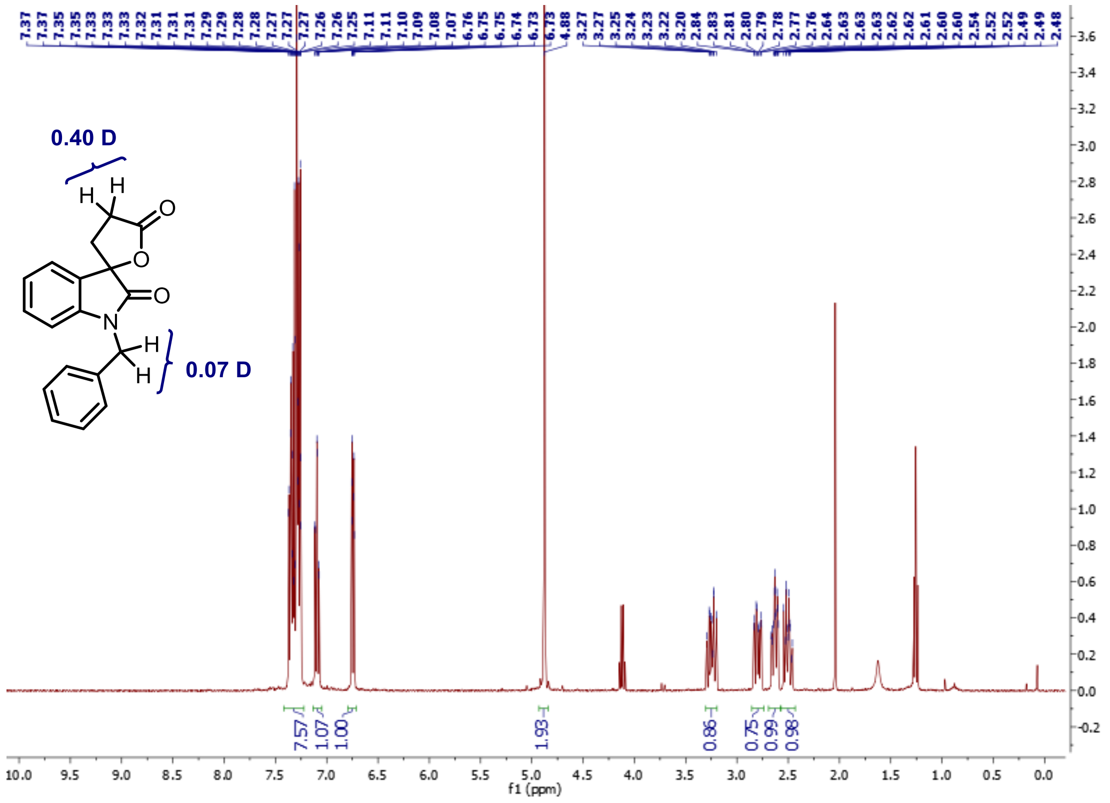

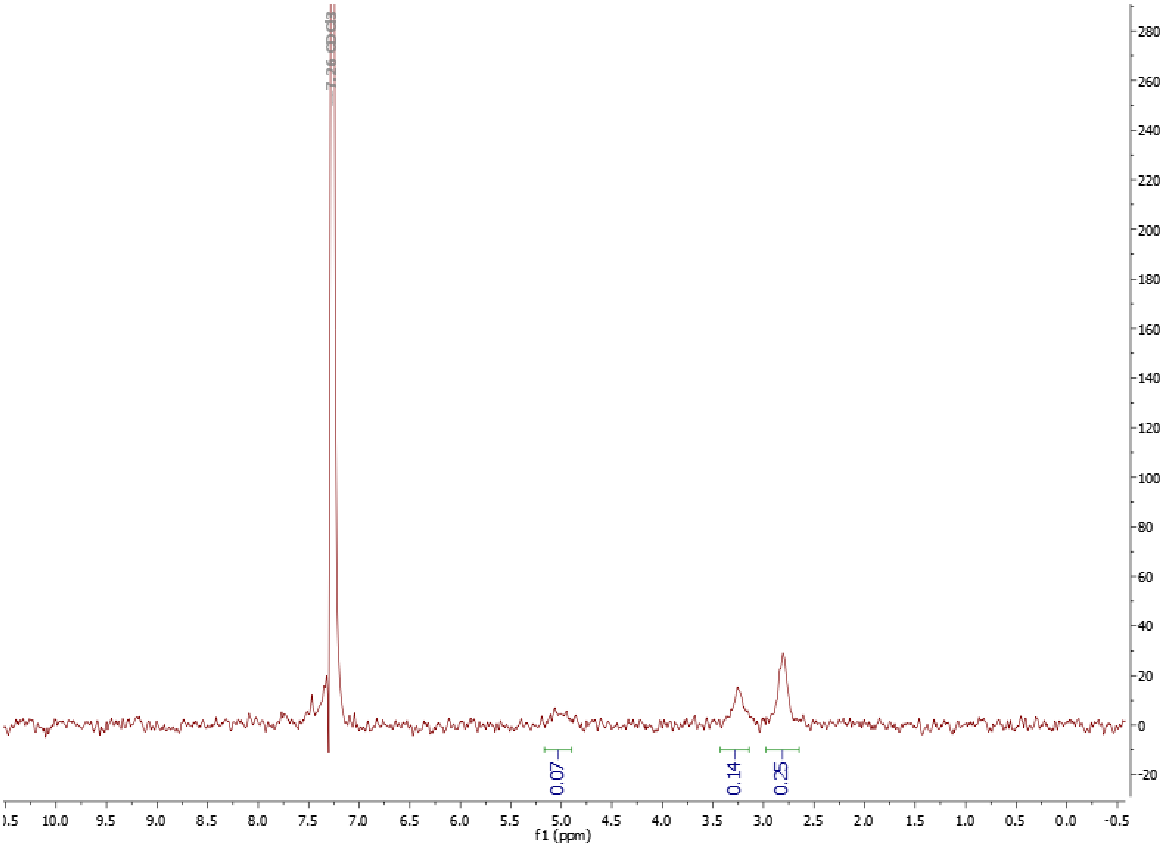

(*E/Z*)-1-Benzyl-7-fluoro-((4-methoxyphenyl)imino)indolin-2-one (10b)

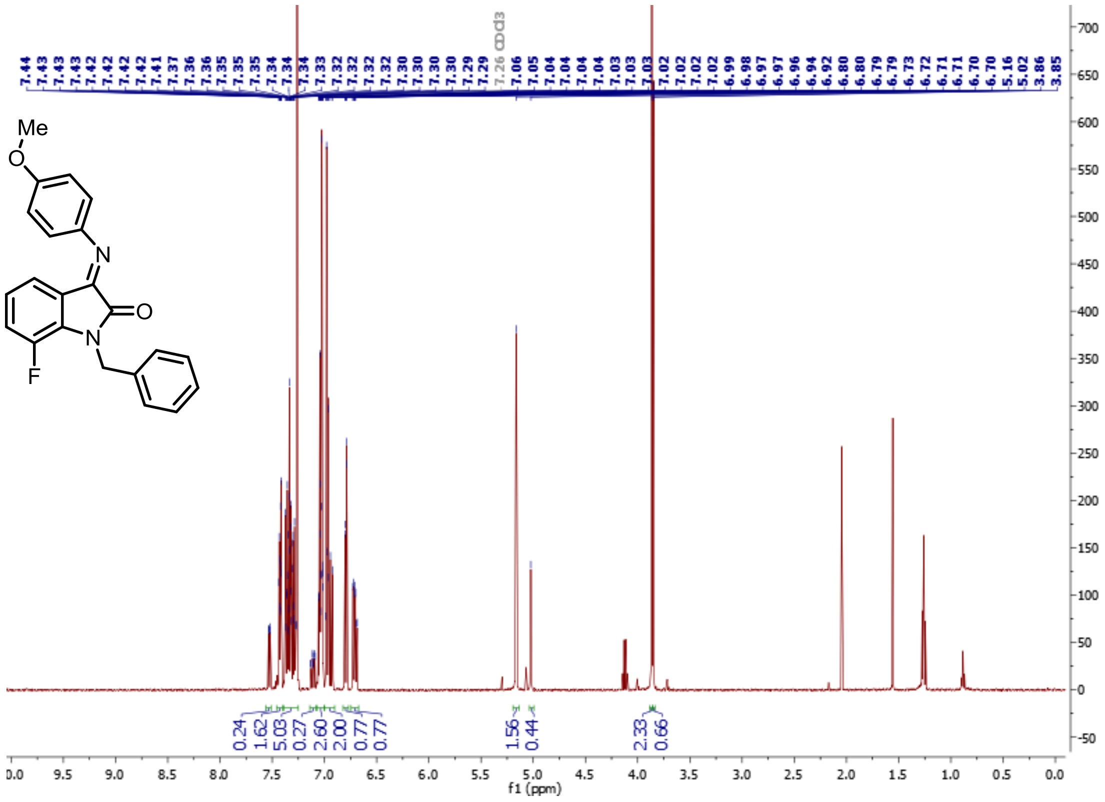

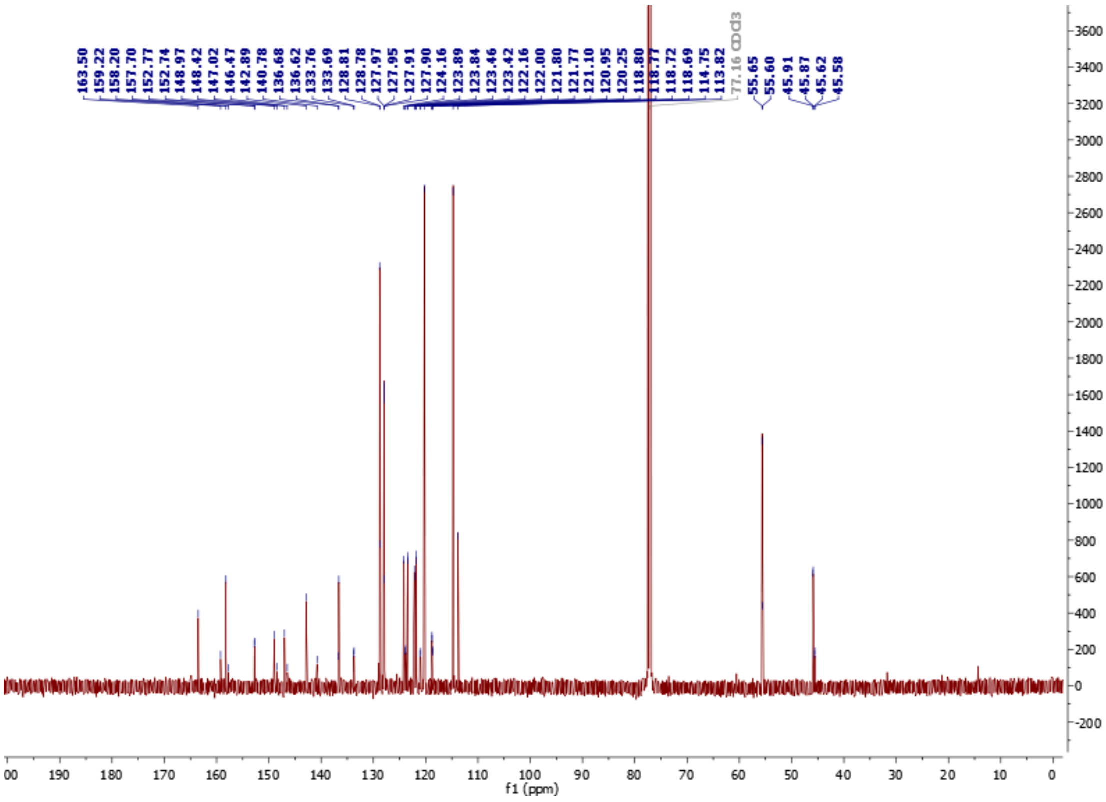

(*E/Z*)-1-Benzyl-3-((4-methoxyphenyl)imino)-7-(trifluoromethyl)indolin-2-one (10c)

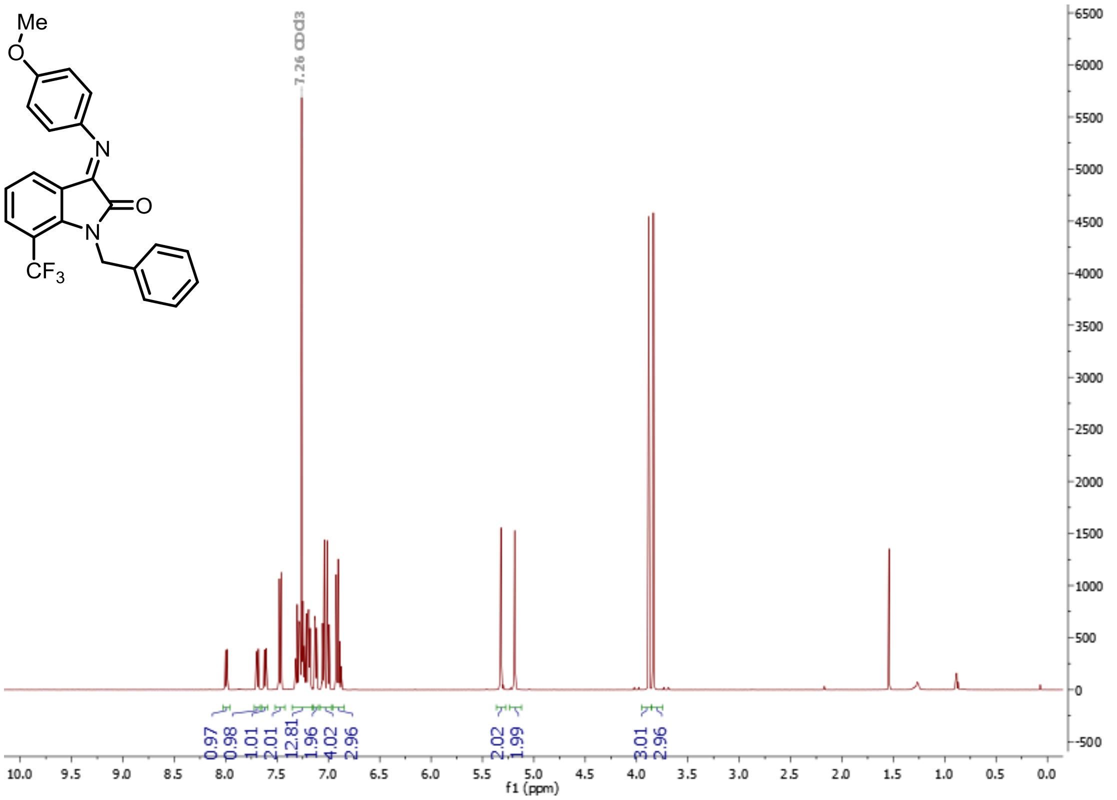

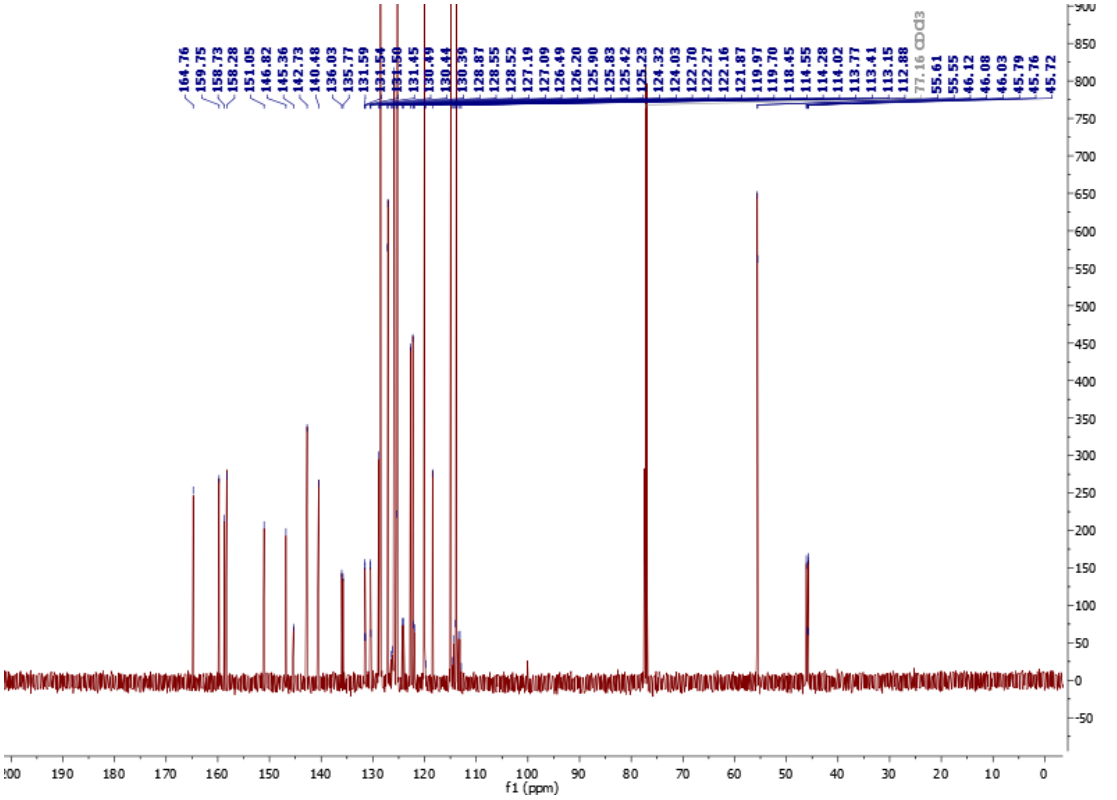

(*E*/*Z*)-1-Benzyl-5-methoxy-3-((4-methoxyphenyl)imino)indolin-2-one (10e)

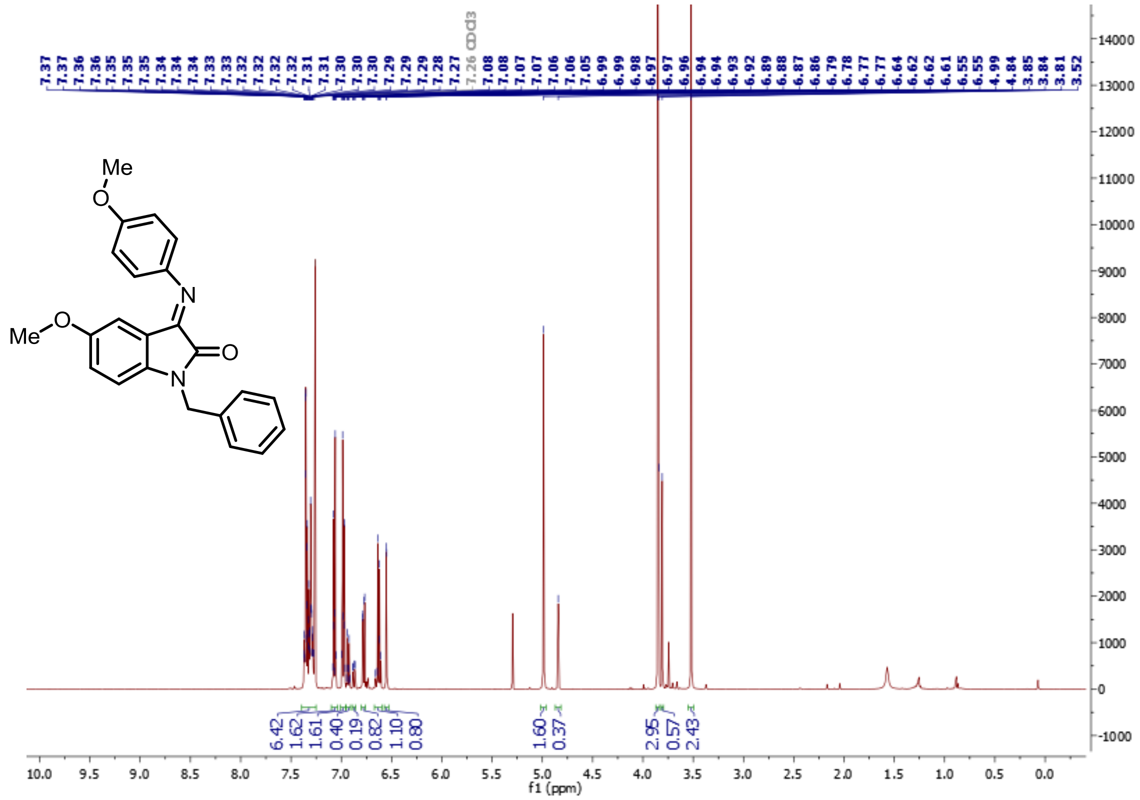

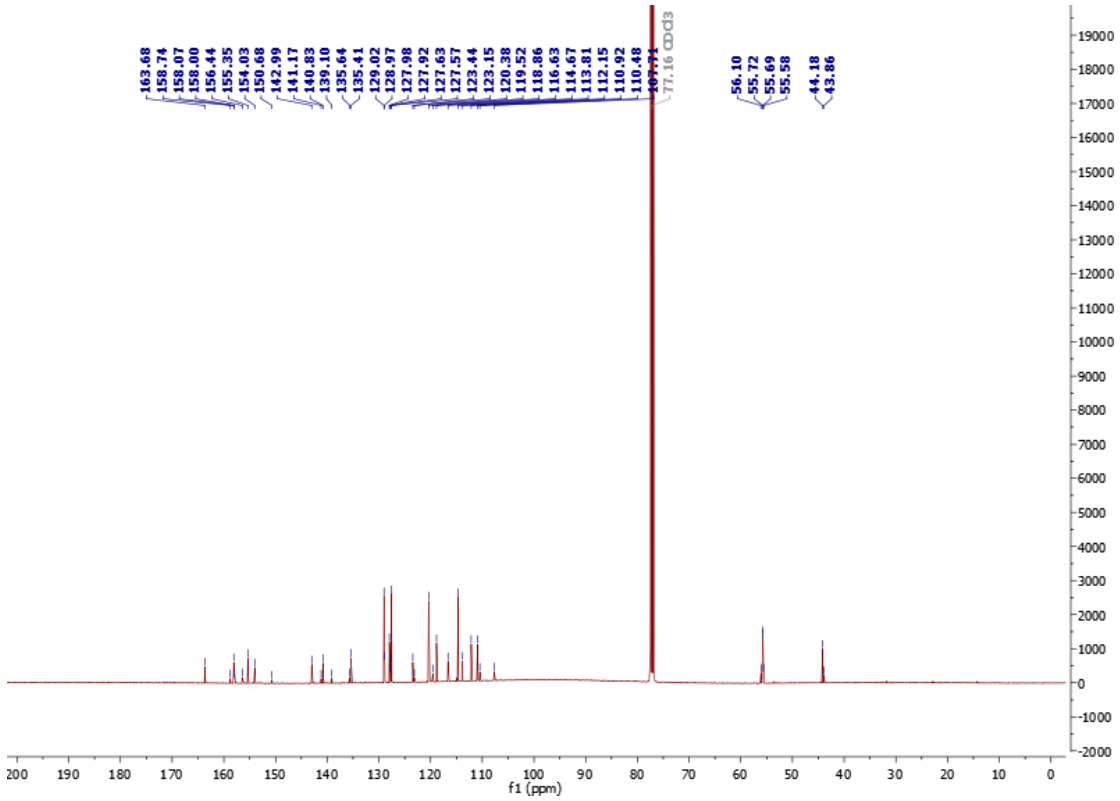

1-Benzyl-1’-(4-methoxyphenyl)spiro[indoline-3,2’-pyrrolidine]-2,5’-dione (11a)

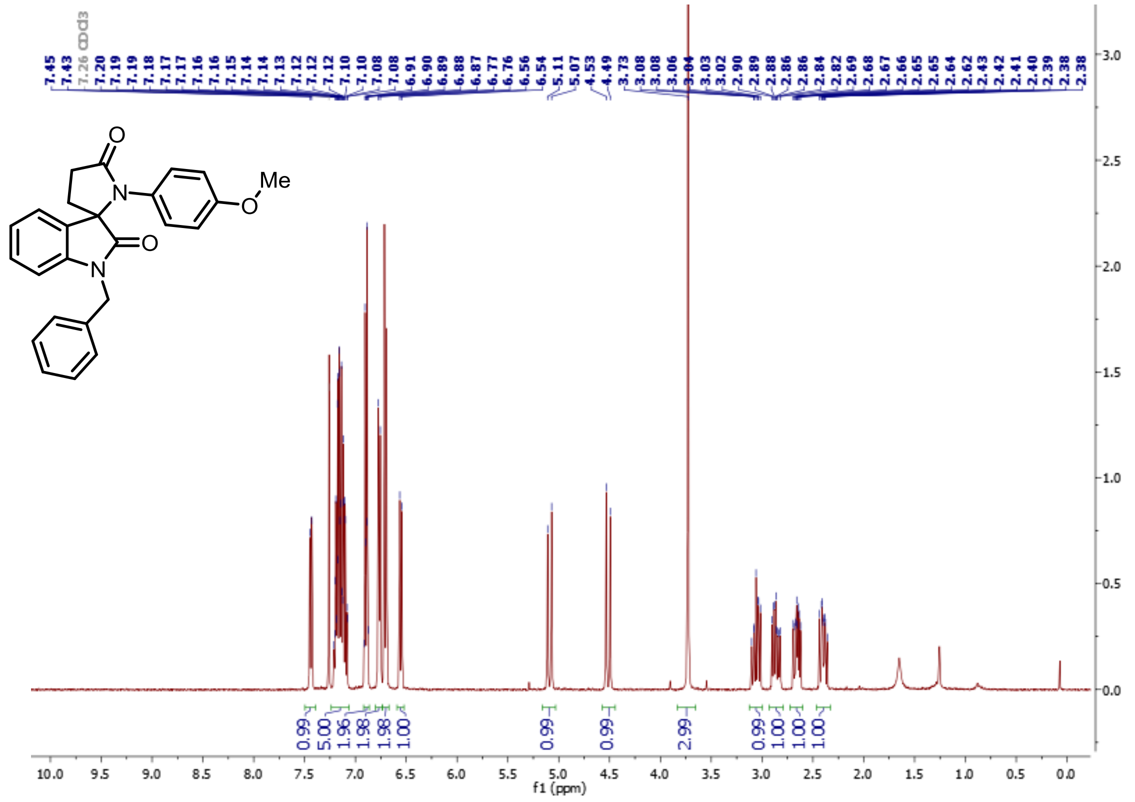

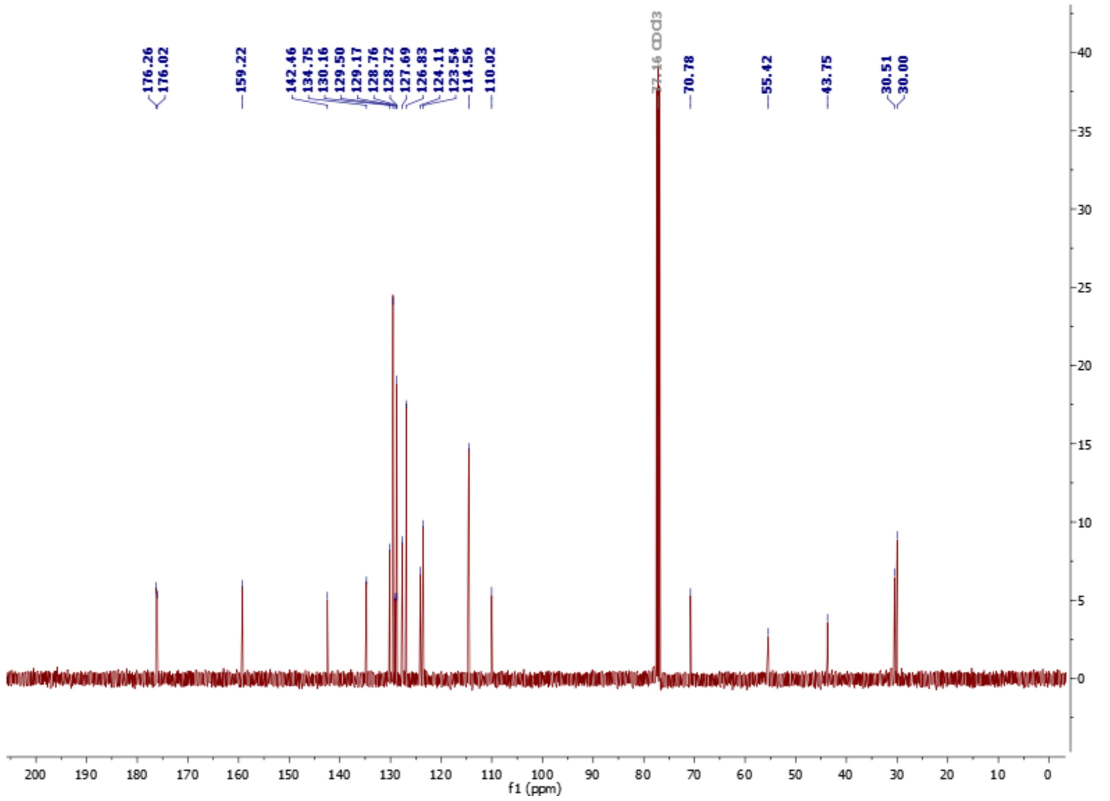

1-Benzyl-7-fluoro-1’-(4-methoxyphenyl)spiro[indoline-3,2’-pyrrolidine]-2,5’-dione (11b)

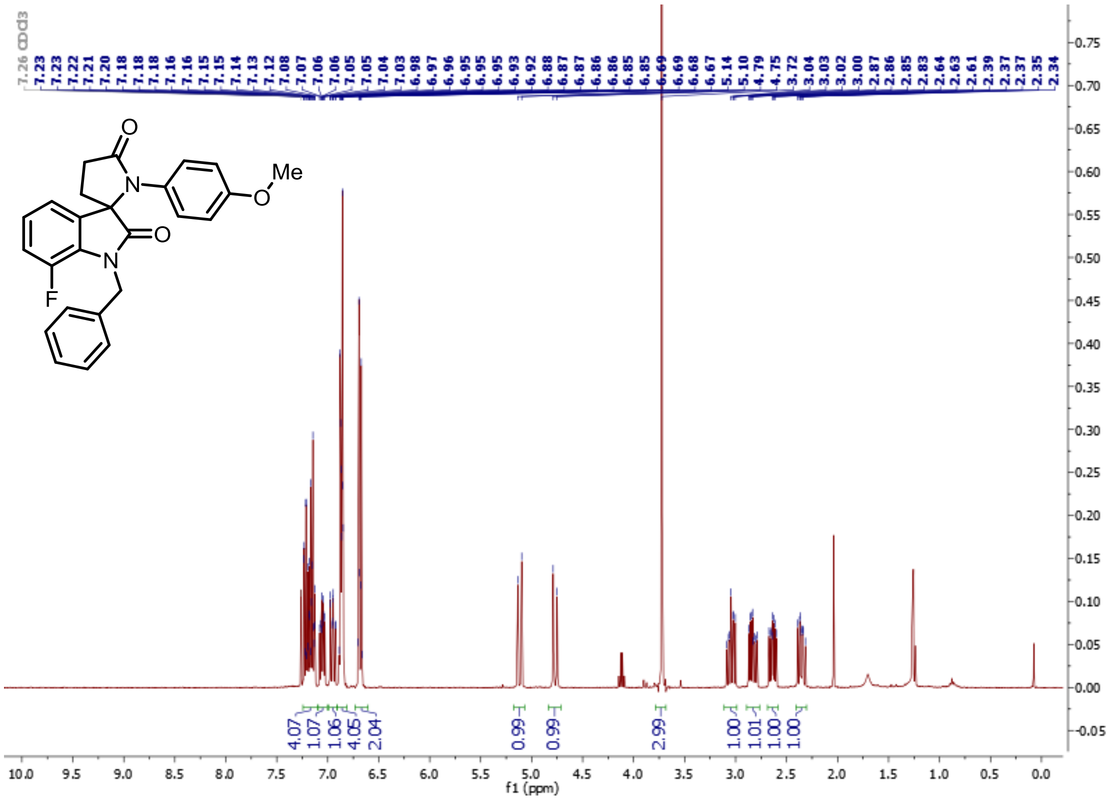

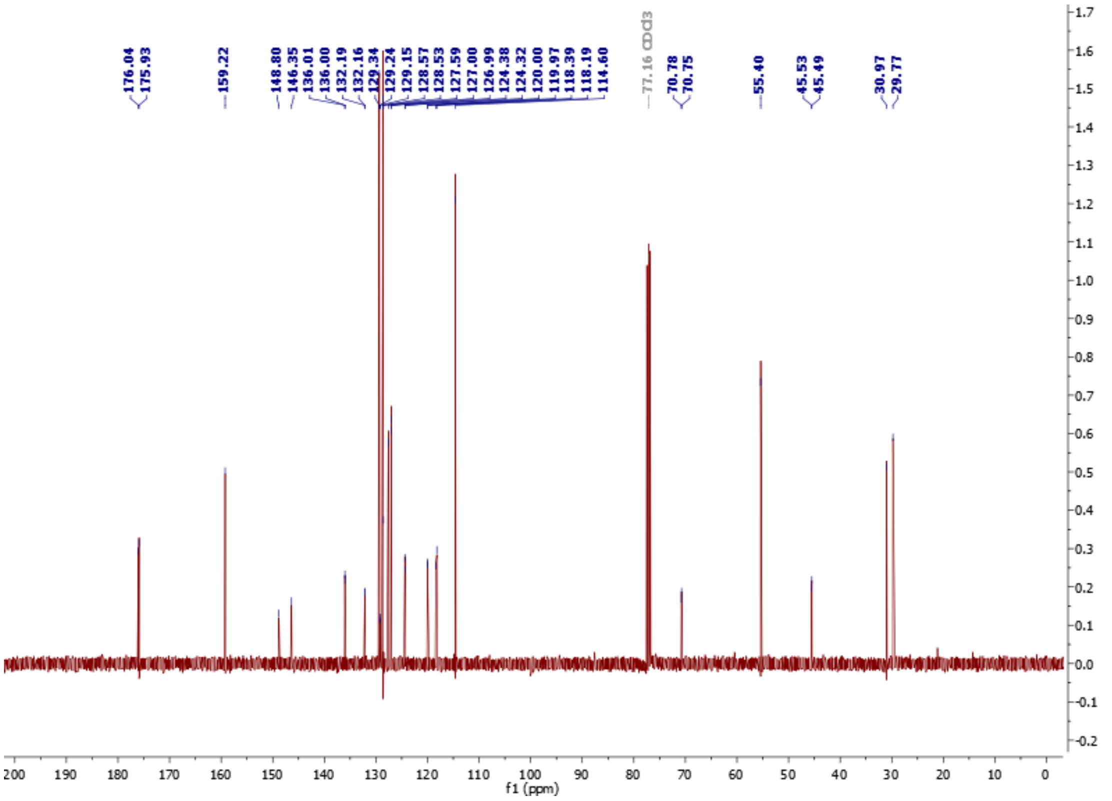

1-Benzyl-1’-(4-methoxyphenyl)-7-(trifluoromethyl)spiro[indoline-3,2’-pyrrolidine]-2,5’-dione (11c)

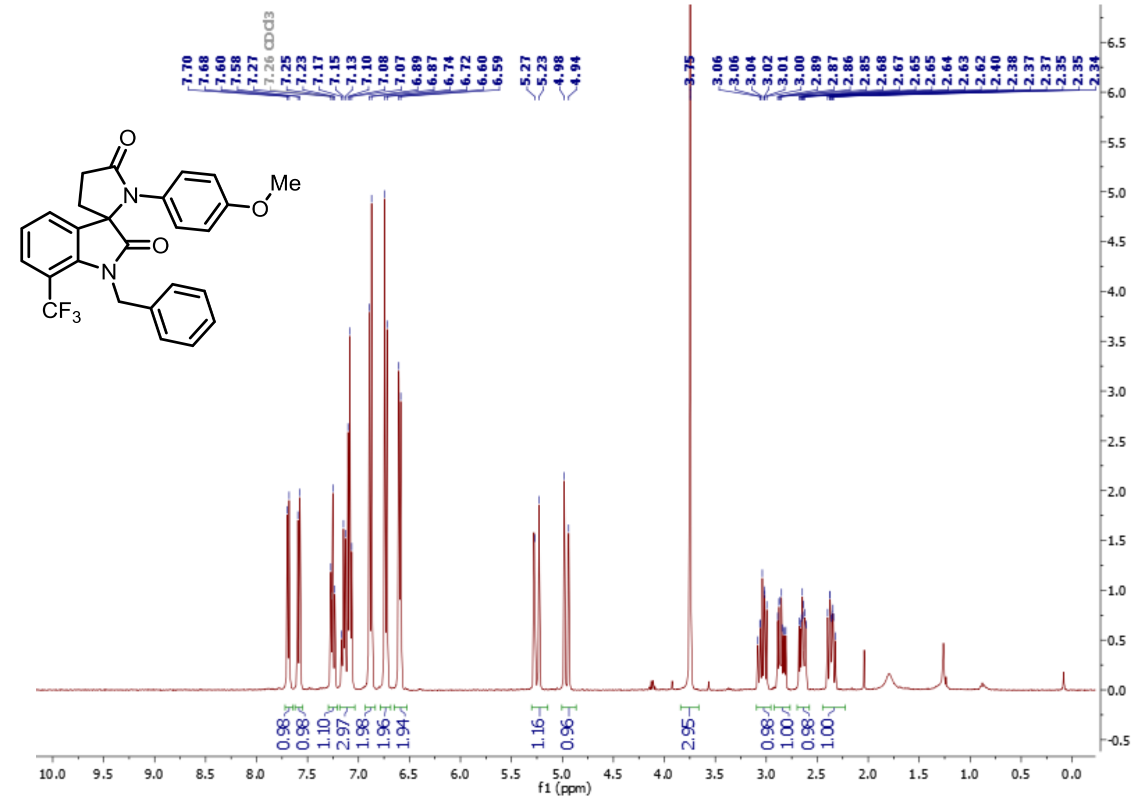

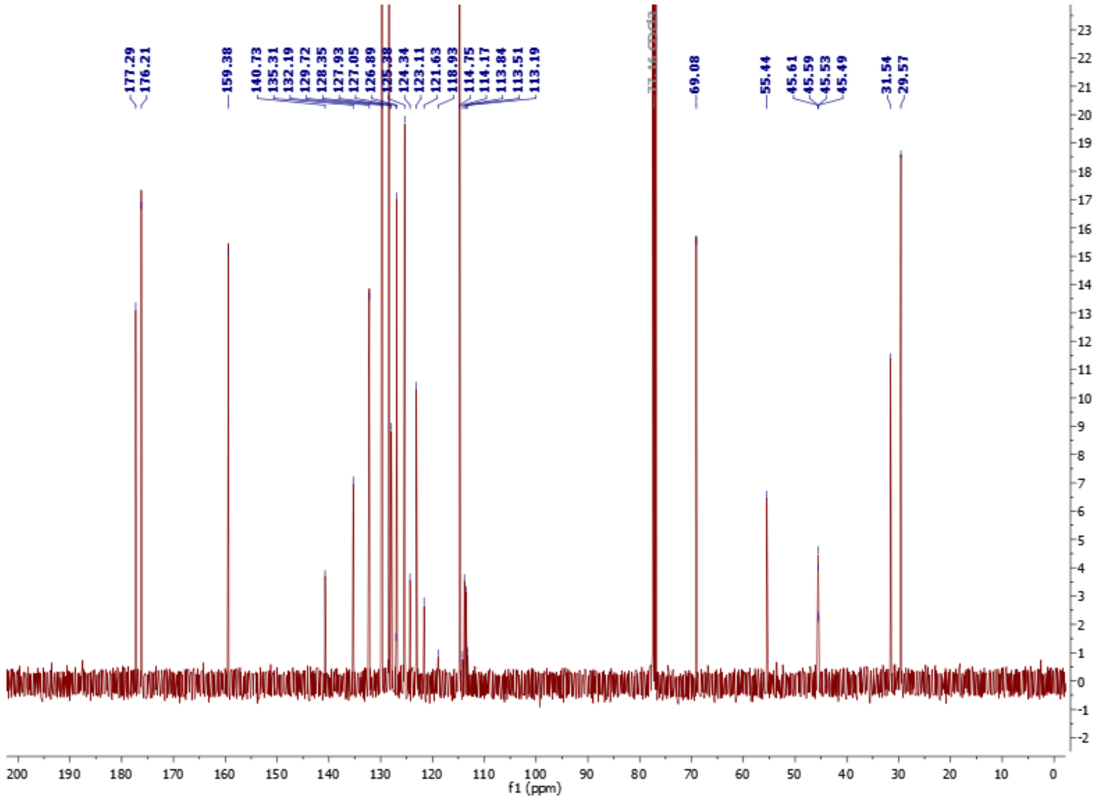

1'-(4-Methoxyphenyl)-1-methylspiro[indoline-3,2'-pyrrolidine]-2,5'-dione (11d)

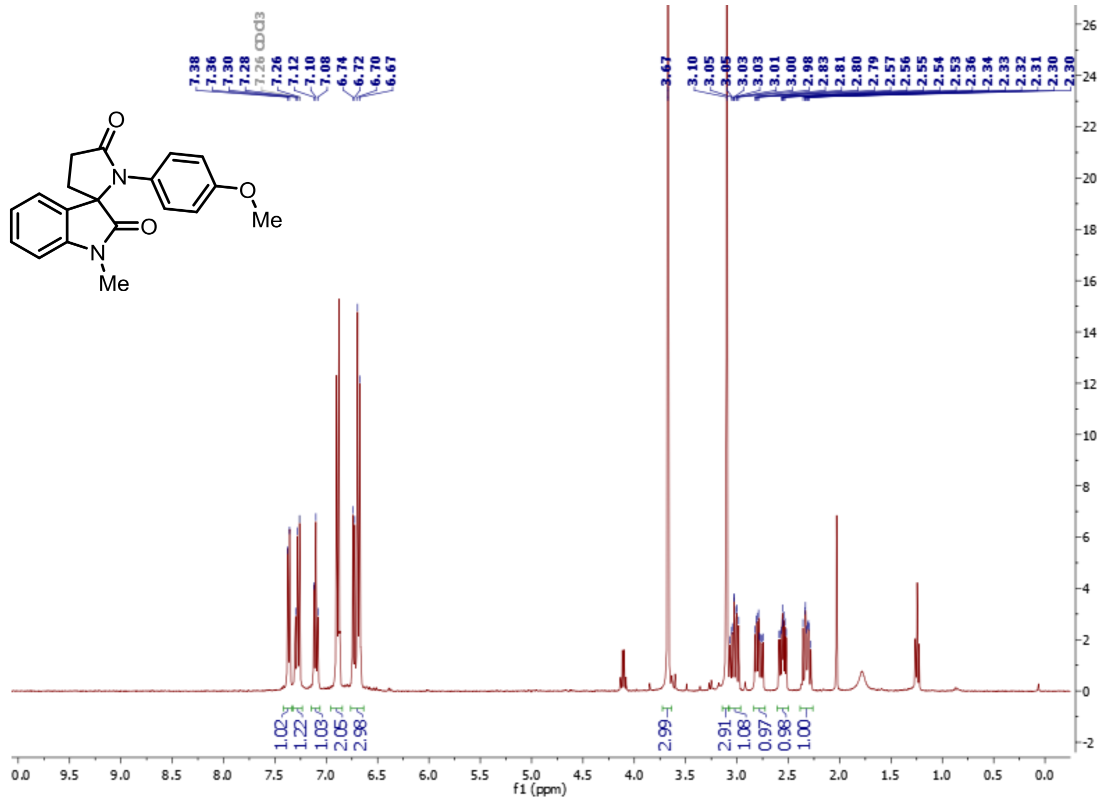

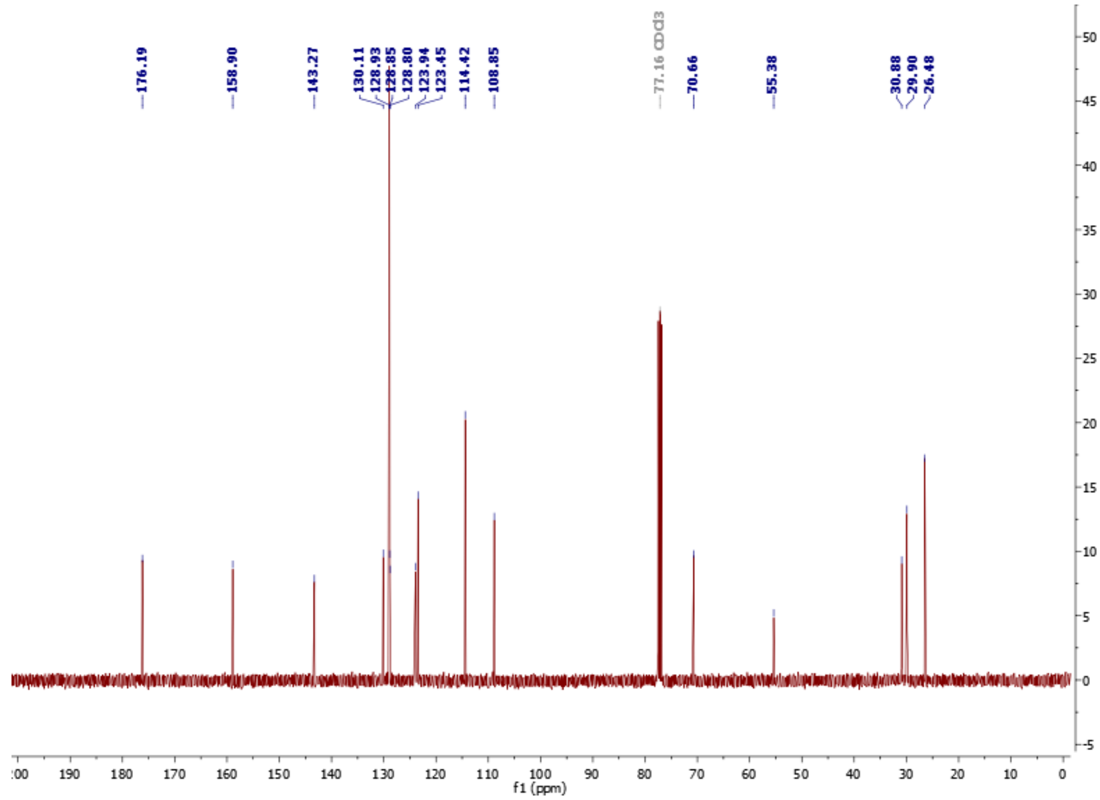

1-Benzyl-5-methoxy-1’-(4-methoxyphenyl)spiro[indoline-3,2’-pyrrolidine]-2,5’dione (11e)

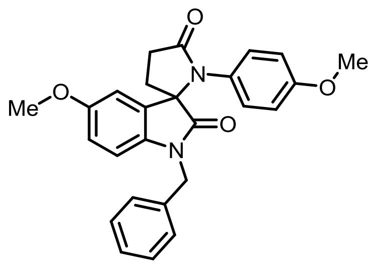

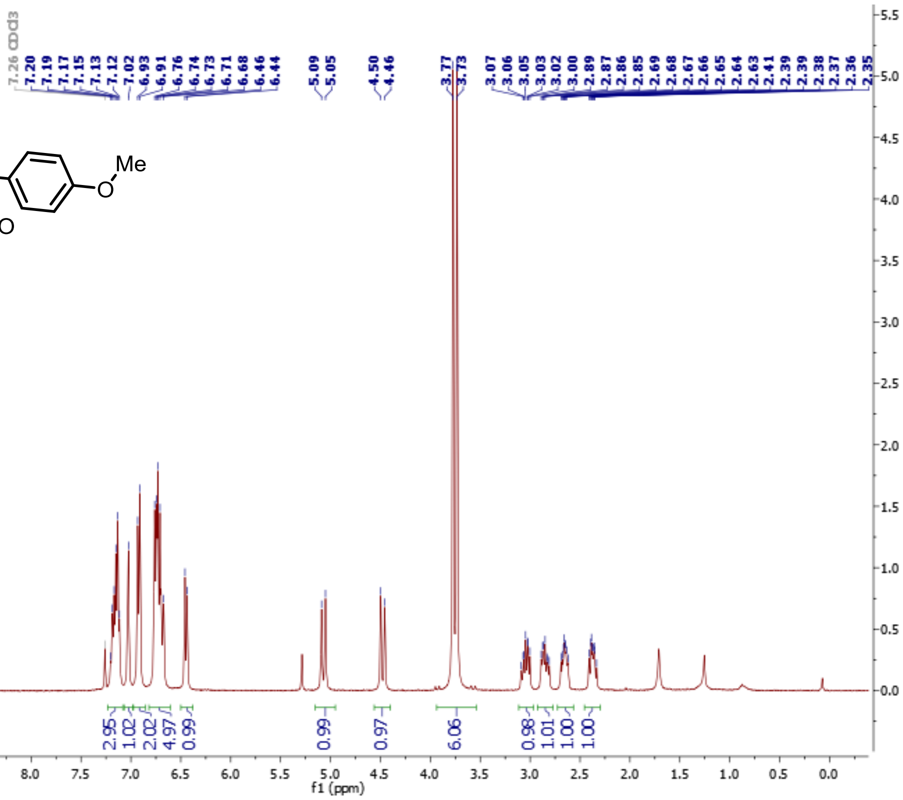

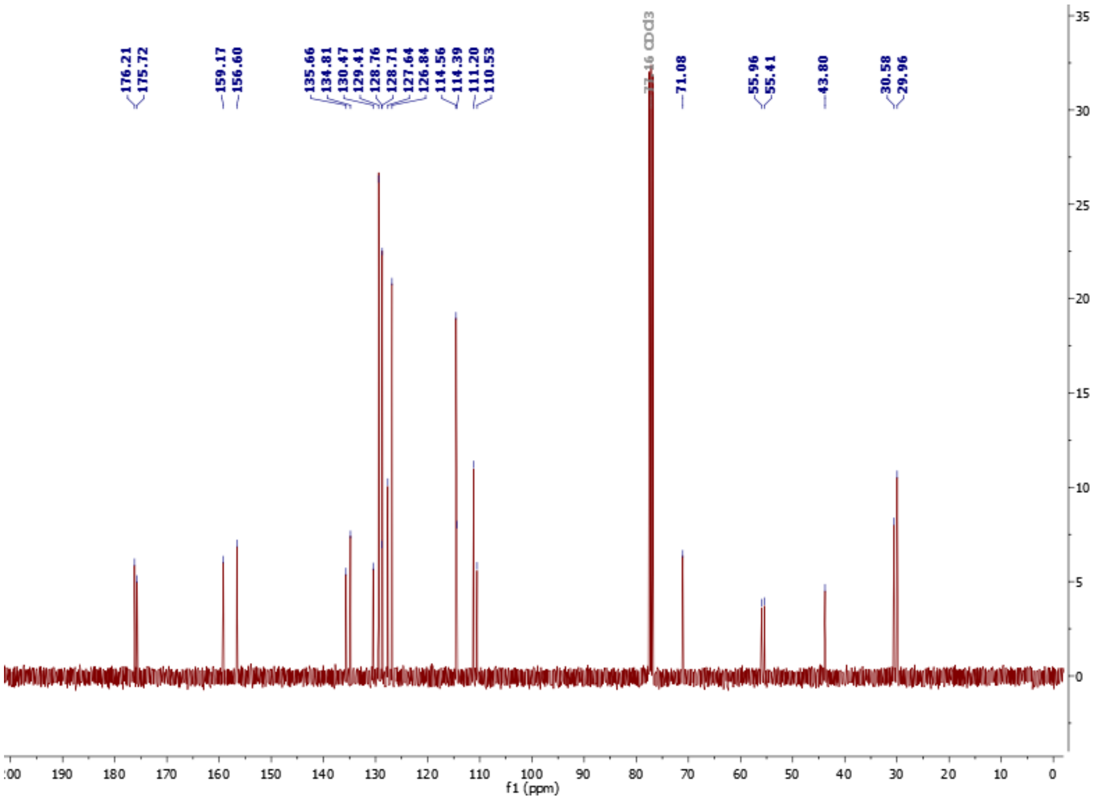

5-Benzoyl-1-(4-methoxyphenyl)-5-phenylpyrrolidin-2-one (12)

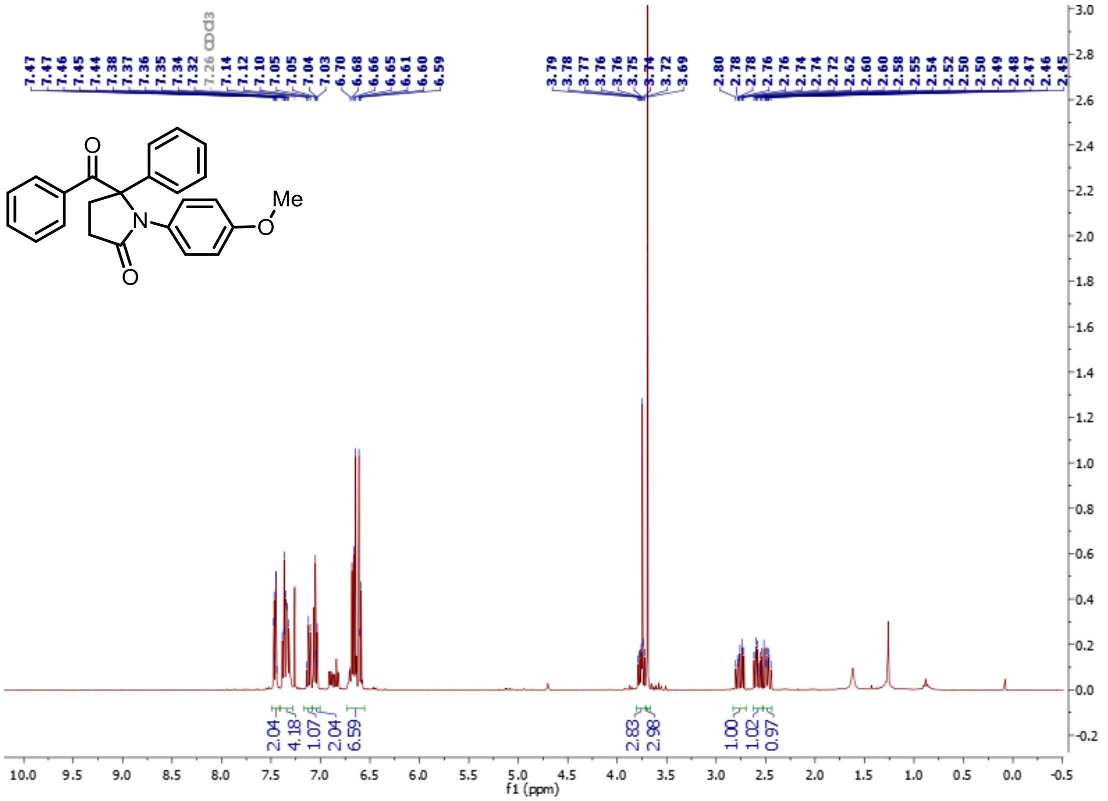

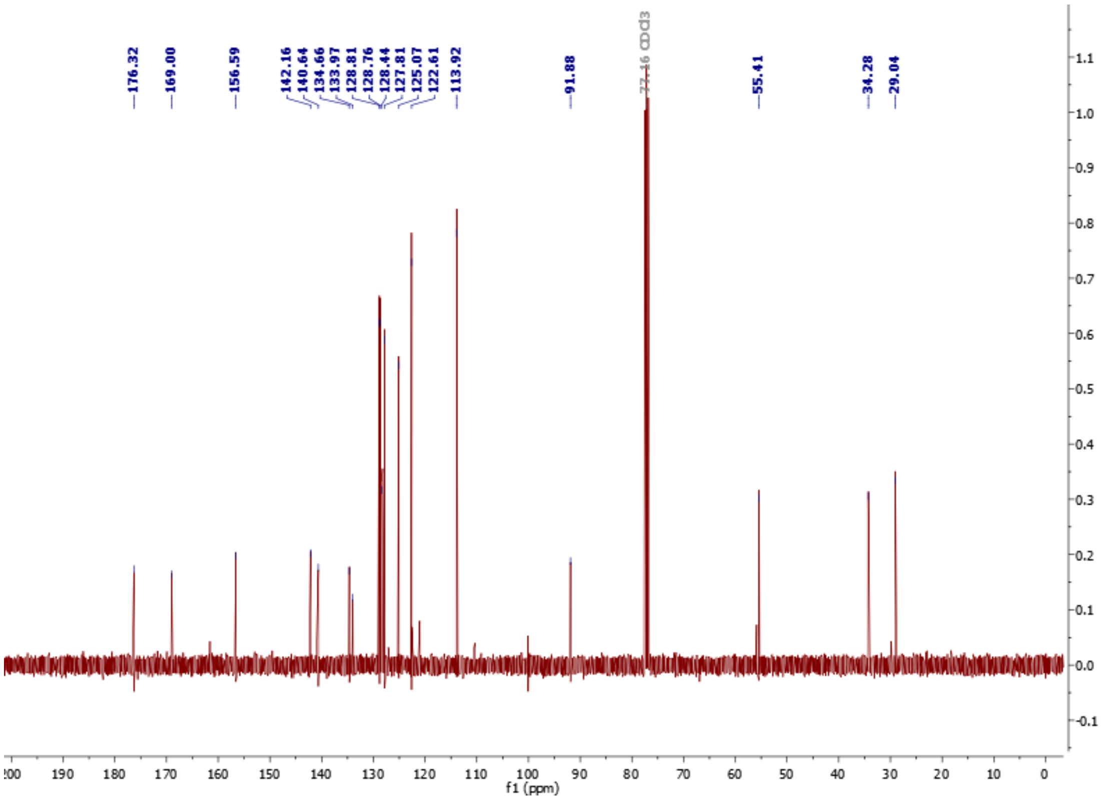

References

1. Thanh, N. D.; Giang, N. T. K.; Quyen, T. H.; Huong, D. T.; Toan, V. N. *Eur. J. Med. Chem.* **2016**, *123*, 532-543.
2. Ogura, Y.; Akakura, M.; Sakakura, A.; Ishihara, K. *Angew. Chem. Int. Ed.* **2013**, *52*, 8299-8303.
3. Satish, G.; Polu, A.; Ramar, T.; Ilangovan, A. *J. Org. Chem.* **2015**, *80*, 5167-5175.
4. Vyas, D. J.; Fröhlich, R.; Oestreich, M. *J. Org. Chem.* **2010**, *75*, 6720-6723.
5. Dhara, K.; Mandal, T.; Das, J.; Dash, J. *Angew. Chem. Int. Ed.* **2015**, *54*, 15831-15835.
6. Itoh, J.; Han, S. B.; Krische, M. J. *Angew. Chem. Int. Ed.* **2009**, *48*, 6313-6316.
7. Shi, F.; Tao, Z.-L.; Luo, S.-W.; Tu, S.-J.; Gong, L.-Z. *Chem. Eur. J.* **2012**, *18*, 6885-6894.
8. Cao, S.-H.; Zhang, X.-C.; Wei, Y.; Shi, M. *Eur. J. Org. Chem.* **2011**, *2011*, 2668-2672.
9. Zhang, H. *J. Chem. Res.* **2014**, *38*, 705-709.
10. Klásek, A. n.; Kořistek, K.; Polis, J.; Košmrlj, J. *Tetrahedron* **2000**, *56*, 1551-1560.
11. Sugahara, K.; Satake, N.; Kamata, K.; Nakajima, T.; Mizuno, N. *Angew. Chem. Int. Ed.* **2014**, *53*, 13248-13252.
12. Gu, P.; Su, Y.; Wu, X.-P.; Sun, J.; Liu, W.; Xue, P.; Li, R. *Org. Lett.* **2012**, *14*, 2246-2249.
13. Feng, X.; Sun, A.; Zhang, S.; Yu, X.; Bao, M. *Org. Lett.* **2013**, *15*, 108-111.
14. Volwiler, E. H.; Vliet, E. B. *J. Am. Chem. Soc.* **1921**, *43*, 1672-1676.
15. (a) Fan, B.; Trant, J. F.; Wong, A. D.; Gillies, E. R. *J. Am. Chem. Soc.* **2014**, *136*, 10116-10123; (b) Mocci, F.; Usai, M.; Cerioni, G. *Magn. Reson. Chem.* **2009**, *47*, 31-37.
16. Hallmann, G. *Chem. Ber.* **1962**, *95*, 1138-1143.
17. McInturff, E. L.; Mowat, J.; Waldeck, A. R.; Krische, M. J. *J. Am. Chem. Soc.* **2013**, *135*, 17230-17235.
18. Xie, Y.; Yu, C.; Li, T.; Tu, S.; Yao, C. *Chem. Eur. J.* **2015**, *21*, 5355-5359.
19. Tobisu, M.; Chatani, N.; Asaumi, T.; Amako, K.; Ie, Y.; Fukumoto, Y.; Murai, S. *J. Am. Chem. Soc.* **2000**, *122*, 12663-12674.
20. Zhang, H.; Lin, X.; Chin, S.; Grinstaff, M. W. *J. Am. Chem. Soc.* **2015**, *137*, 12660-12666.
21. Trost, B. M.; Hirano, K. *Org. Lett.* **2012**, *14*, 2446-2449.
22. Silvi, M.; Chatterjee, I.; Liu, Y.; Melchiorre, P. *Angew. Chem. Int. Ed.* **2013**, *52*, 10780-10783.
23. Shi, Y.-H.; Wang, Z.; Shi, Y.; Deng, W.-P. *Tetrahedron* **2012**, *68*, 3649-3653.
24. González, A.; Quirante, J.; Nieto, J.; Almeida, M. R.; Saraiva, M. J.; Planas, A.; Arsequell, G.; Valencia, G. *Bioorg. Med. Chem. Lett.* **2009**, *19*, 5270-5273.
25. Cabrera, A.; Sharma, P.; Ayala, M.; Rubio-Perez, L.; Amézquita-Valencia, M. *Tetrahedron Lett.* **2011**, *52*, 6758-6762.
